# New Rigid Polycyclic Bis(phosphane) for Asymmetric Catalysis

**DOI:** 10.3390/molecules24030571

**Published:** 2019-02-05

**Authors:** K. Michał Pietrusiewicz, Katarzyna Szwaczko, Barbara Mirosław, Izabela Dybała, Radomir Jasiński, Oleg M. Demchuk

**Affiliations:** 1 Faculty of Chemistry, Maria Curie-Sklodowska University, 33-Gliniana St., 20-031 Lublin, Poland; Kazimierz.Pietrusiewicz@poczta.umcs.lublin.pl (K.M.P.); O.Demchuk@IFarm.eu (O.M.D.); 2 Faculty of Pharmacy, Medical University of Lublin, 4^A^-Chodźki St., 20-093 Lublin, Poland; izalis1@gazeta.pl; 3 Department of Organic Chemistry, Cracow University of Technology, 24-Warszawska St., 31-155 Cracow, Poland; radomir@chemia.pk.edu.pl; 4 Pharmaceutical Research Institute, 8-Rydygiera St., 01-793 Warsaw, Poland

**Keywords:** bis(phosphane) palladium complex, metallacycle, NORPHOS, allylic alkylation, asymmetric catalysis, chiral phosphines, C-H bond activation, polycyclic compounds, stereoselective synthesis, DFT calculations

## Abstract

A simple, highly efficient synthesis of a series of novel chiral non-racemic rigid tetracyclic phosphorus ligands, applicable in important chemical asymmetric transformations, was performed. In a tandem cross-coupling/C-H bond activation reaction, a well-recognised and readily available ligand *(R,R)-*NORPHOS was used as the starting material. The palladium complexes of new ligands were obtained and characterised on the example of a crystalline dichloropalladium complex of [(1*R*,2*R*,9*S*,10*S*,11*R*,12*R*)-4-phenyltetracyclo[8.2.1.0^2,9^.0^3,8^]trideca-3,5,7-triene-11,12-diyl]bis(diphenylphosphane). A notably high activity and stereoselectivity of the palladium catalysts based on the new ligands were confirmed in a model asymmetric allylic substitution reaction. Herein, we discuss the geometry of the palladium complexes formed and its impact on the efficiency of the catalysts. A comparison of their geometric features with other bis(phosphane) ligand complexes found in the Cambridge Structural Database and built density functional theory (DFT) commutated models is also presented and rationalised.

## 1. Introduction

One of the most challenging approaches in contemporary synthetic organic chemistry is the selective activation of C-H bonds leading to the creation of new carbon-carbon bonds in the structure of fine organic compounds of commercial value. A palladium-catalysed Heck coupling reaction is one of the major tools used for the C-C bond formation. The reaction is widely used to prepare drugs, fragrances and agrochemicals [1,2,3]. An extension of the regular alkenes arylation according to the Heck coupling mechanism is the palladium-catalysed Heck–Catellani reaction, reported in 1985, which involves an intramolecular C-H bond activation and functionalisation [4]. This discovery provided the foundation for the majority of recent developments of direct C-H activation/C-C coupling reactions [5,6,7,8,9] that are conducted to improve the efficiency and atom economy of traditional couplings.

**Scheme 1:**
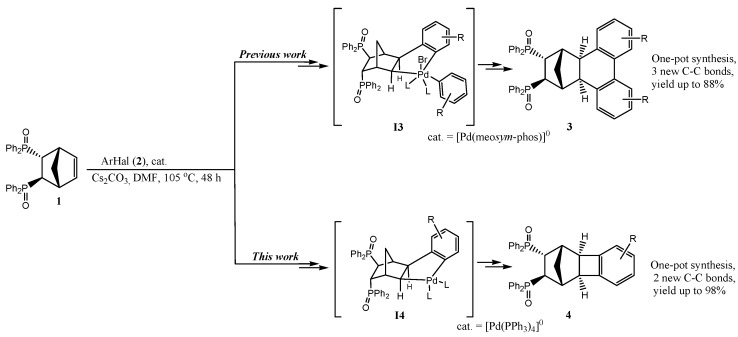
Synthesis of polycyclic NORPHOS derivatives.

Chiral biphosphine ligands are a powerful synthetic tool for generating complex and diverse chemical compounds. Recently, we have reported a new methodology for a highly regio- and stereoselective reaction of the NORPHOS oxide (**1**) [10,11] with substituted aryl bromides (**2**), which furnished various pentacyclic NORPHOS oxide derivatives **3** (Scheme 1) [12]. This new type of the Heck–Catellani reaction proceeded through tandem cross-coupling/cross-dehydrogenative-coupling stages, mediated by highly active palladium complexes of bulky electron-rich bidentate mono phosphines (S-Phos, *Sym*-Phos and MeO*Sym*-Phos) [12,13]. Inspired by these achievements, we have been prompted to explore some new opportunities that a reaction of the Heck–Catellani type could provide. It had been mentioned in [12] that in the reaction leading to pentacyclic NORPHOS oxide derivatives (**3**), some by-products with molecular weights that formally satisfy chemical formulae of classical mono-arylated Heck products were formed in trace amounts. Since the formation of the Heck reaction product is rather non-typical for norbornene substrates, we supposed that the observed minor by-product could have the structure of a tetracyclic compound (**4**). Interestingly, the best chiral ligands used in the transition-metal-mediated asymmetric synthesis display chirality in the backbones of the ligand and a rigid structure weakly influenced by random conformational changes [14]. This phenomenon has its roots in the fact that the catalysts with restricted freedom of conformational movements more significantly differentiate the competitive transition states leading to enantiomeric reaction products. Thus, C-chiral chelating phosphanes whose stereometry is stabilised by polycyclic systems, especially those based on five- and four-membered rings, could be considered as promising candidates for efficient ligands to be used in catalytic transition-metal-mediated asymmetric transformations. A new, efficient, highly stereo- and regioselective approach to the synthesis of tetracyclic compound (**4**), using readily available substrates and inexpensive commercial palladium catalysts, should be a welcome addition to our previously reported methods for the synthesis of valuable polycyclic bis(phosphane) ligands.

Herein, we disclose a transition-metal-catalysed, one-pot stereoselective synthesis of [tetracyclo[8.2.1.0^2,9^.0^3,8^]trideca-3,5,7-triene-11,12-diyl]bis(diphenylphosphane) dioxides: novel NORPHOS oxide derivatives bearing a benzocyclobutene motif. After deoxygenation, the obtained phosphanes could be used as ligands in transition-metal-mediated asymmetric transformations. We also present a short summary of the geometry in the pentametallacyclic complexes of bis(phosphones) with transition metals found in the Cambridge Structural Database, which were compared with the dichloropalladium complex of phosphane derived from **4**.

## 2. Results and Discussion

Utilisation of most active ligands, such as MeO*Sym-*Phos: Electron-rich and bulky, according to the basic concept [15,16], should accelerate the oxidative addition of aromatic halides to Pd(0) species and, therefore, a double addition of aromatic halides should predominate in the reaction [12]. Thus, in order to direct the reaction to an alternative path of mono-arylation, an electron-deficient ligand of a relatively small steric hindrance, which stabilises intermediate **I4** and facilitates the reductive elimination to product **4,** should be used (Scheme 1). Tetrakis(triphenylphosphine)palladium(0) was therefore selected to be used as the catalyst. The two-component reactions of racemic NORPHOS oxide (*rac-***1**) and aryl halides (bromide or iodide) used in an equimolar amount or in a 3-fold excess were carried out in the presence of Pd(PPh_3_)_4_, bases and solvents at 105 °C for 12–48 h. We found that a proper choice of the base and solvent was crucial to achieving high yields. Among the tested bases, including K_3_PO_4_, KOAc, KO*^t^*Bu, K_2_CO_3_ and others (see electronic supplementary information (ESI)), Cs_2_CO_3_ was selected as the most promising, which significantly increased the yield of desirable product **4**. Polar aprotic solvents are often used in the coupling reaction as they allow for the best solubility of the substrates and the catalysts. In the model reaction between **1** and *p*-bromotoluene (1.2 equiv.) run in dimethylformamide (DMF) in the presence of 5 mol% of [Pd(PPh_3_)_4_] and caesium carbonate (3.0 equiv.) at 105 °C for 48 h, the isolated yield of product **4b** was 83%. A study of the solvent effects on the yield of the coupling reaction revealed that while the reaction proceeds well in toluene (yield 79%), in THF (yields 72%) and in acetonitrile (yield 65%), the catalytic process is greatly affected in DMF (see ESI). Compared to other combination of used bases and solvents, Cs_2_CO_3_ solubilised in DMF is less likely to coordinate to the palladium centre and, therefore, less likely to interfere with the catalyst activity. To obtain a satisfactory conversion in DMF, 5 mol% of [Pd(PPh_3_)_4_] was required. The presence of 1 mol% and 0.1 mol% of the phosphine palladium complex resulted in the formation of 45% and 21% yields of the product, respectively (see ESI).

Having optimised the conditions, we prepared a series of phosphine oxides **4**. Table 1 presents the isolated yields of products **3** and **4** bearing diversified substituents in the introduced arenes.

The coupling reaction of ortho-substituted aryl halides with **1** gave cyclobutene derivatives **4** with a very good yield. A higher yield of **4** was observed when the proportion of the aryl halide was increased from 1.2 to 3.0 equiv. Thus, in the reaction of **1** with 1.2 equiv. or with 3.0 equiv. of *o*-bromotoluene (**2f**), the desired product was isolated in 78% and 85% of the yield, respectively (Table 1, entry 6). Under similar conditions, a tri-substituted aryl halide (Table 1, entry 8) allows us to achieve a similar 86% yield. Using [Pd(PPh_3_)_4_], a generally low-activity catalyst, allows us to conduct the reaction between **1** and 2-bromochlorobenzene (**2g**) chemoselectively, and only product **4g** was isolated in a 54% yield. In the case of 2-bromobiphenyl (**2q**), the reaction furnished tetracyclic product [tetracyclo[8.2.1.0^2,9^.0^3,8^]trideca-3,5,7-triene-11,12-diyl]bis(diphenylphosphane) dioxides (**4q**) in an excellent 98% yield.

**Scheme 2:**
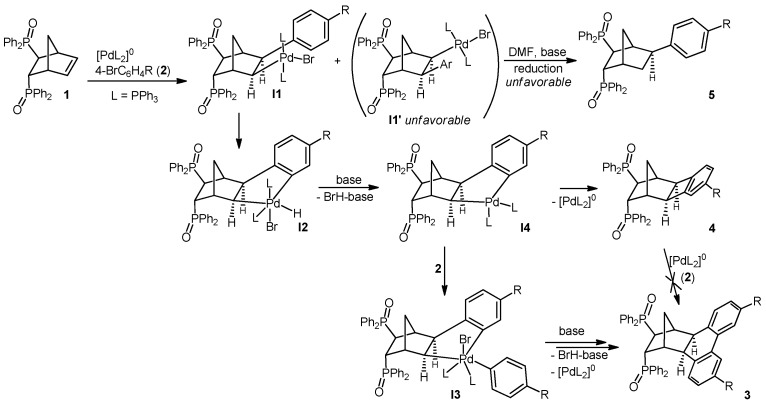
A tentative suggestion for the molecular mechanism of the coupling reaction.

Surprisingly, we have found that the direction of the reaction depends not only on the catalyst used, but also on the nature of the aryl halide. The NORPHOS oxide underwent the reaction with highly active aromatic halides, e.g., possessing an electron-withdrawing groups (EWG) (**2j**, **2k**) or a trimethylsilyl (TMS) group (**2l**) and with unsubstituted phenyl bromide (**2a**) furnishing the mixture of products **3** and **4** (Table 1, entries 1, 10, and 11) with the ratio of **3** to **4** increasing in line with the increasing excess of substrates **2** in the reaction mixture. This observation supports the suggestion that the selection of the reaction path depends on the relation of the rates of conversion from intermediates **I4** to **4** and **I4** to **I3** (Scheme 2). Thus, using “fast”, electron-rich and bulky catalysts and active substrates, due to rapid halo-arylation of the palladium atom in **I4**, facilitates the formation of **I4** and the next product **3**. In contrast to that, utilisation of less-active, electron-deficient and ligand-based catalysts and electron-donating group (EDG) substituted substrates resulted in the formation of product **4** in higher yields.

Our methodology can also be extended to non-benzenoid aromatic bromides (including naphthalenes and phenanthrenes). Both 1-bromonaphthalene (**2m**) and 2-bromo-6-methoxynaphthalene (**2n**) smoothly underwent the reaction with **1** to give the corresponding carbocyclisation products **4m** and **4n** in 53% and 68% yields, respectively (Table 1, entry 13, 14). A similar 54% yield of **4p** was obtained in the case of 9-bromophenanthrene (**2p**) (Table 1, entry 16). As could be expected, the halides that do not possess any hydrogen atoms in position ortho- to the aryl–halogen bond did not undergo the studied reaction (Table 1, entry 15). Moreover, our attempts to obtain heterocyclic products **3e** or **4e** failed, perhaps due to the strong coordination of the nitrogen atom of pyridine to palladium (Table 1, entry 5).

### 2.1. General Mechanism and Selectivity of the Reaction

**Figure 1:**
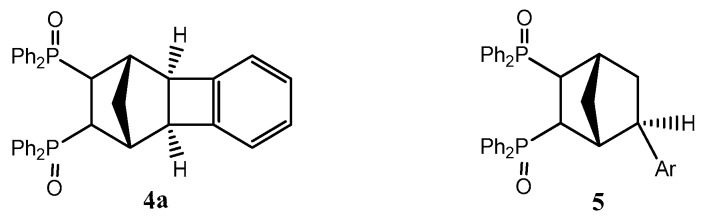
Side reaction products **4a** and **5**.

The catalytic carbocyclisation reaction between the NOPHROS oxide and aryl halides that resulted in cyclobutene derivatives **4** is highly regioselective, giving the cis-exo isomer as the exclusive product. An unexpected side reaction product **4a** (2–10%) was detected in the reaction mixtures by the ^1^H-NMR and HPLC-HR-MS spectral analyses in almost all cases when differently substituted aromatic halides were used (Figure 1). We could assume that, as an exclusive source of phenyls, the phenylidene fragment originated from triphenylphosphane. The migration of the phenyl substituent from the phosphorus atom to palladium had already been observed in the course of other studies [17]. The molecular mechanism of this rare transformation is not clear and its elucidation requires additional theoretical as well as experimental investigations.

A tentative mechanism of the reaction leading to products **3**–**5** includes several stages (Scheme 2). The first obvious step of the reaction is an oxidative addition of **2** to the palladium catalysts; then, an insertion of the obtained arylpalladium intermediate into the double bond of the NORPHOS oxide furnished intermediate **I1**. Two possible isomeric intermediates **I1** and **I1’** could be formed. Nevertheless, only a single type of isomeric product was found in the reaction mixtures, and was identified as **3** and **4**. This could be rationalised by the steric hindrance created by P(O)Ph_2_ groups, which forces large aryl substituents to occupy a less-hindered position. Obviously, both isomeric products of the alternative endo insertion of arylpalladium halides to **1** are significantly unfavorable and were not formed. The second reaction step is an intramolecular activation of the ortho C-H bond of the introduced arene by palladium, which resulted in the formation of intermediate **I2**. A base facilitated elimination of the hydrogen halide at the next step, which led to the reduction of the oxidation stage of palladium in intermediate **I4**. Next, when using electron-rich ligands of the *C,P-*type transition metal binding [12,13] or highly active aromatic halides, the oxidative addition of the second molecule of **2** occurs and the reaction runs via intermediate **I3** towards product **3**. Thus, in the case of less-active catalysts and non-activated substrates, intermediate **I4** undergoes reductive elimination to form product **4**.

We have also examined the possibility of product **4**’s conversion to **3,** which theoretically may occur if an excess of the aromatic halide is present in the reaction mixture by the activation of a strained cyclobutane ring with reactive palladium species. For this purpose, in the presence of the palladium complex of the S-Phos ligand and caesium carbonate (3.0 equiv.), compound **4** was subjected to the reactions with 3.0 equiv. of aryl bromides (**2a** and **2b**). After 48 h in DMF at 105 °C, we did not observe the formation of compound **3** and did not find any evidence that such a reaction could be conducted under the studied conditions.

Since both products **3** and **4** are formed from the same intermediate **I4,** it could be anticipated that a lower rate of the oxidative addition reaction, inherent to the electron-poor, ligand-based catalyst, will facilitate the formation of product **4**.

Additionally, in the cases of Cs_2_CO_3_ (due to its notably strong basic character) and polycyclic aromatic substrates **2m** and **2p**, trace amounts of reductive Heck reaction products **5,** formed by reducing intermediate **I1**, presumably by a solvent [18,19], were detected in the postreaction mixtures (Figure 1).

### 2.2. Preparation of Chiral Non-Racemic Ligand ***6q***

**Scheme 3:**
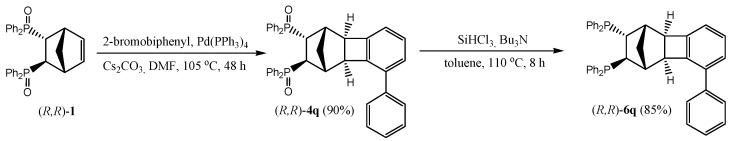
Synthesis of the phosphine ligand.

Having developed such a high-yielding procedure for the synthesis of desirable highly rigid bis(phosphane) dioxides, we aimed to conduct the synthesis of new chiral ligands to be used in asymmetric transition metal complex-catalysed reactions. We decided to use our new methodology to synthesise representative chiral non-racemic phosphane **6q**, since the corresponding bis(phosphane) dioxide **4q** was obtained in the highest yield. First of all, the scaleup of the synthesis of **4q** was performed. Notably, the synthesis conducted in a scale that increased up to one gram of *(R,R)*-**1** furnished the product in a 90% yield without the need of further optimisation of the reaction conditions. The obtained bis(phosphane) dioxide underwent a deoxygenation reaction in the presence of trichlorosilane and tributylamine, affording ligand *(R,R)*-**6q** in an 85% yield (Scheme 3). Ligand **6q** appeared to be sufficiently resistant to exposure to air and could be purified chromatographically. Using the enantiomerically pure, readily available substrate *(R,R)*-**1** in the synthesis allows us to obtain an enantiomerically pure product with no need of practical-scale enantiopurification.

### 2.3. Synthesis of the Complex [Pd(6q)Cl_2_]

Since the validation of the proper geometry of the formed ligand by means of NMR spectroscopy is not a trivial matter, we have decided to perform it by analysing the single crystal X-ray diffraction data of the derived dichloropalladium complex [Pd(**6q**)Cl_2_]. The complex was prepared from [Pd(*^t-^*BuCN)_2_Cl_2_] and ligand **6q** in tetrahydrofuran (THF), and precipitated after a partial replacement of the solvent with C_6_H_6_. A single crystal has been grown from the hot ethanol/benzene mixture.

### 2.4. Molecular and Crystal Structure of the Complex [Pd(6q)Cl_2_]

The palladium coordination compound [Pd(**6q**)Cl_2_] has been characterised by a single crystal X-ray diffraction analysis (CCDC No.: 1885894). [Pd(**6q**)Cl_2_] crystallises in a tetragonal crystal system in the chiral *P*4_3_2_1_2 space group. The absolute structure assignment of the complex [Pd(**6q**)Cl_2_] has been determined through anomalous scattering with the value of the Flack parameter refined to -0.007(3), using 2963 quotients (for more details, crystal data and structure refinements see (ESI).

**Figure 2:**
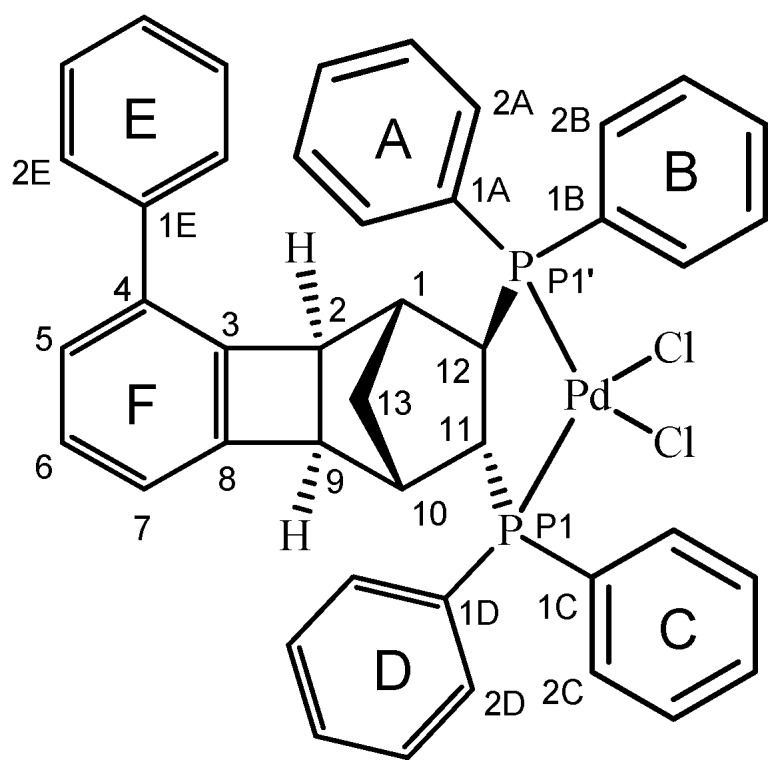
The molecular structure of the complex [Pd(**6q**)Cl_2_].

**Figure 3:**
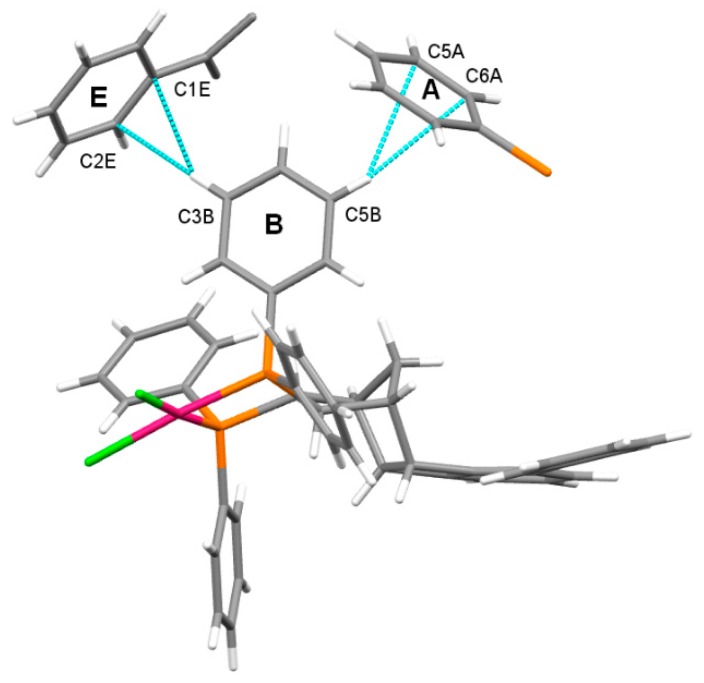
T-shaped π…π interactions in the complex [Pd(**6q**)Cl_2_].

The chiral centers are localised at the C1 (*R*), C2 (*R*), C9 (*S*), C10 (*S*), C11 (*R*) and C12 (*R*) atoms (Figure 2). The bond lengths and selected angles are given in Table 2 and Table 3. An X-ray analysis confirmed the cis, exo configuration of the hydrocarbon skeleton at the C2–C9 joint atoms. The hydrogens attached to the C11 and C12 atoms are located in a trans configuration, which is in accordance with the synthesis route (Table 2 and Table 3, Scheme 3, Figure 2). In the four-membered ring, the valence angles show strains resulting in a distortion of the *sp*^3^- and *sp*^2^- hybridised carbon atom centers from the ideal 109.5 and 120° to 86 and 94°, respectively, with higher values being observed at the side of the aromatic ring (Table 2 and Table 3). The phenyl ring E is nearly coplanar with the ring F (torsion C2E–C1E–C4–C3 14.6(6)°). The complex [Pd(**6q**)Cl_2_] is an example of metallacycles, which are known to be reactive intermediates in a catalysis. This five-membered ring is formed by one Pd, two P and two C atoms and has the shape of an envelope with the C12 atom located at the flap, which deviates by 0.835(4) Å from the mean plane of Pd1–P1–C11–P1′. The puckering amplitude (maximum out-of-plane deviation) q2 calculated for this metallacycle was 1.142(5) Å and the phase angle describing the puckering (phase shift) Φ2 was 153.8(1)° [20]. The Pd metal center has a planar square coordination with two cis-located Cl atoms. Ligand **6q** acts as a chelating bidentate agent forming two Pd–P coordination bonds of lengths equal to 2.2463(8) and 2.2722(9) Å. There are no classic hydrogen bonds in the crystal structure. The molecules interact through C–H…Cl contacts and T-shaped π…π interactions (Table 4, Figure 3).

### 2.5. Geometry of Five-Membered Metallacycles of the Transition Metals and Bis(phosphine) Ligands Found in the Cambridge Structural Database (CSD)

In order to better understand the reactivity and stereochemistry of the bis(phosphane) metalorganic catalysts, a search of the CSD [21] for the structures of five-membered metallacycles with a transition metal and the P–C(HC)–C(HC)–P fragment of bis(phosphane) has been performed. It gave a result of 120 hits, 27 of which were the structures of complexes with Pd (CSD Refcodes: DEPXAX, GEPQUN, HOGBUA, MIDPIY, MIDPOE, NEXHOP, NIDYIK, NURKAM, OFAQET, OFAQIX, OFAQUJ, OKOHAX, PAQBIS, PAQBOY, POPZEA, QELSAB, QUBGID, QUBGOJ, RIYGUB, RONPOZ, RUDJAC, RUNVUR, TESBEZ, TIYPAS, VASXUI, WORDIQ and YOBWUH).

**Figure 4:**
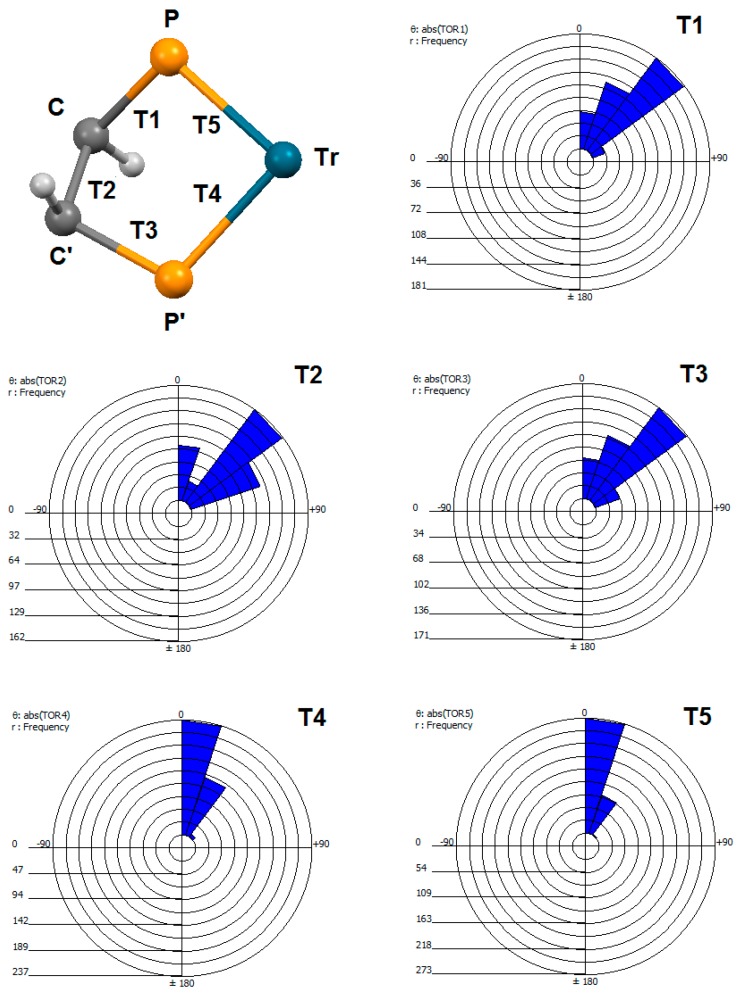
Distribution of the torsion values T1–T5 in the structures of five-membered metallacycles in the complexes of transition metals (Tr) with bis(phosphane) ligands found in the CSD.

The endocyclic torsion angles characterising this system are summarised in Table 5, and the distribution of their absolute values is presented in Figure 4. There is a clear difference in the most frequently occurring values. The dihedral angles T4 and the corresponding one T5 (C–P–Tr–P) were most frequently synperiplanar, whereas torsions T1–T3 were usually synclinal. In most of the structures, the metallacycle had a conformation of an envelope, as in the presented complex [Pd(**6q**)Cl_2_], with the exception of five entries where the ring was almost flat (CSD Refcodes: EQUDEA (Ru), HAYREE (Pt), HAYRII (Pt), VISRAR (Os) and ZOVCIY (Fe)) [22,23]. Usually, one of the carbon atoms was located at the envelope flap (the same as in [Pd(**6q**)Cl_2_]), providing a favourable tetragonal flat coordination environment near the metal atom and the asymmetric structure of the metallacycle. Only in three palladium complexes was the metal atom positioned at the flap instead of the carbon atom (in the structures where the carbon atoms belonged to the four-membered hydrocarbon ring and the exocyclic H–C–C–H torsion indicated a cis configuration; CSD Refcodes: NIDYIK, QUBGID and QUBGOJ) [24,25]. However, in analogous Pd complexes with the four-membered hydrocarbon ring and the trans configuration at the same torsion, the metallacycle ring had, as usual, a carbon atom at the envelope flap (CSD Refcodes: OFAQET, OFAQIX and OFAQUJ) [26]. This asymmetry observed in the metallacycle structure is probably beneficial for transition-metal-promoted asymmetric transformations.

The analysis of the orientation of the H atoms within the metallacycle showed that cis stereoisomers were nearly half of the hits (54 of 120). In the presented complex [Pd(**6q**)Cl_2_], the H–C–C–H torsion showed the trans configuration. To illustrate the differences within this group of crystal structures, they were divided into four subsets according to the value of the H–C–C–H torsion. There were 17 hits with synperiplanar torsions, 37 synclinal, 6 anticlinal and 60 antiperiplanar as in the presented complex [Pd(**6q**)Cl_2_] (Table 2, Table 3, Table 4 and Table 5). The results showed that the most frequent conformation for the five-membered metallacycles of transition metals and bis(phosphane) ligands was the envelope type conformation, regardless of the transition metal and phosphine substituent types.

### 2.6. Quantum Chemical Considerations

The quantum chemical calculation performed at the M062x/LANL2DZ theoretical level [27,28], which is specially dedicated to precise energetic considerations and has recently been applied in the simulation of energetic and structural analyses of molecules with similar structural moieties [29,30,31,32,33], allows us to compare the geometry of the molecules of phosphines **6**, their dioxides and complexes that were not crystallised in crystals, well-measurable by a single crystal X-ray diffraction. The geometry optimisation for the structure [Pd(**6q**)Cl_2_] was therefore performed and the obtained geometry parameters of [Pd(**6q**)Cl_2_]*^calc^* were compared with those received from the X-ray diffraction experiments for [Pd(**6q**)Cl_2_]*^xray^*. It was found that all key interatomic distances (Table 6) are characterised by the values that are typical for the respective bonds in similar organic moieties [13,33,34,35]. The comparison of the obtained geometries with the respective X-ray structures confirms that the density functional theory (DFT) calculations at the M062x/LANL2DZ theoretical level illustrate well the key structural aspects of the considered complexes.

**Figure 5:**
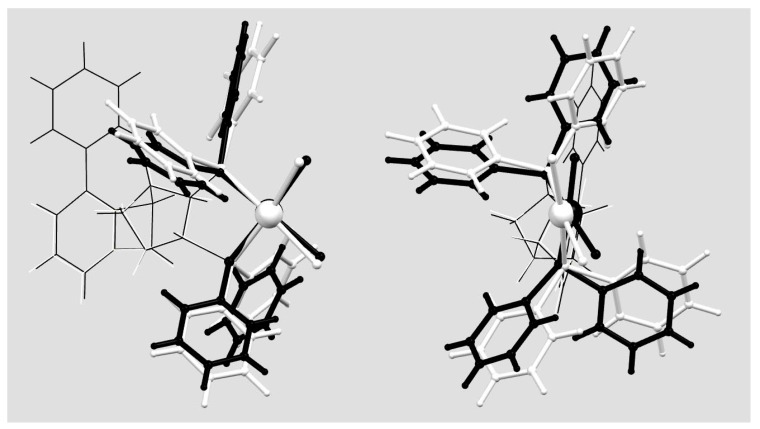
The structural overlay of the molecules of the coordination compounds [Pd(**6q**)Cl_2_]*^xray^* and [Pd(**6q**)Cl_2_]*^calc^*: front and side views. Black: measured X-ray structure [Pd(**6q**)Cl_2_]*^xray^*, white: calculated geometry of [Pd(**6q**)Cl_2_]*^calc^*. The fragments that match are marked with thin lines.

The fitting of molecular structures [Pd(**6q**)Cl_2_]*^xray^* and [Pd(**6q**)Cl_2_]*^calc^* clearly shows in which fragments the differences are observed (Figure 5). The molecular overlay indicates that, due to its rigidity, the polycyclic skeleton is identical both in the experimental and theoretical models. Thus, some minor differentiation is observed in the position of the phenyl substituted at the phosphorus atom. This could be explained by the fact that the calculation of a single molecule in vacuum, also with a consideration of a solvent, cannot correctly reflect all crystal net forces influencing the geometry of a molecule in a solid state. The structural comparison is summarised in Table 6. The comparison of the theoretical model and the experimental structure proved that there is a high degree of similarity of their geometries in the polycyclic core.

Moreover, analogous DFT calculations for the complex [Pd(**6a**)Cl_2_]*^calc^* and bis(phosphane) dioxides **4a** and **4q** were carried out to investigate the influence of the substitutes on the skeleton geometry. All geometries (experimentally determined and optimised using DFT calculations) were compared with each other. Selected bond lengths and angles are summarised in Table 6.

We found a regularity regarding the calculated bonds lengths of the molecules: they are slightly larger than the measured ones. This is a normal phenomenon, considering the fact that the calculation concerns an isolated molecule [36]. The key bond lengths and other geometrical parameters in [Pd(**6q**)Cl_2_]*^calc^* and [Pd(**6a**)Cl_2_]*^calc^* and the **4q** and **4a** molecules are the same (within 3σ). The presence of a phenyl substituent in [Pd(**6q**)Cl_2_]*^calc^* does not have any influence on the geometry of the hydrocarbon polycyclic fragment. In the crystal structure [Pd(**6q**)Cl_2_]*^xray^,* the phenyl ring E is nearly coplanar with the skeleton moiety (ring F, torsion 14.6°), while in the optimised model of [Pd(**6q**)Cl_2_]*^calc^,* the corresponding planes are twisted by 30°. The coplanarity of these fragments is stabilised in the crystal by the C-H⋅⋅⋅Cl bonds in which the carbon atoms of the phenyl ring are involved, and, therefore, it could not have been observed in the cases of isolated single molecules nor in the solution.

Some minor differences in the torsion angle P1-C11-C12-P2 were found in the geometries of molecules **4q** and **4a** in comparison to those of the complexes [Pd(**6q**)Cl_2_]*^calc^* and [Pd(**6a**)Cl_2_]*^calc^*. A smaller value of this parameter in the complex results from the necessity of flattening this molecular fragment in the palladium complexation process, but not from introducing a substituent in position C4 of the polycyclic core of the ligand. Differences in the molecular structures related to the position of the phenyl rings, which are caused by a free rotation around the P-1C*_Ph_* σ-bonds, are not essential for the catalytic efficiency of the ligand-derived palladium complexes.

To summarise, different substitutions did not affect the stereochemistry of the polycyclic hydrocarbon moiety of the molecules. The main structural differences were observed in the orientation of the phenyl substituents attached to the phosphorus atoms that were involved in the thermal movements around single P-C bonds. Even the complexation of the ligand with palladium did not significantly change the rigid geometry of the ligands. Thus, such unique stereometrical properties of the ligands allow us to extrapolate, via computer modelling, the geometry of the selected measured structures to the stereometry of other ligands in this group.

### 2.7. The Evaluation of the Efficiency of the Ligand ***6q*** Palladium Complexes in the Asymmetric Catalysis

The potential of bis(phosphane) ligand **6q** was examined in a model Pd-catalysed allylic alkylation reaction between *rac-(E*)-1,3-diphenylallyl acetate and dimethyl malonate using BSA/KOAc and K_2_CO_3_/Cs_2_CO_3_ as bases in THF at ambient temperature (Table 7). The catalyst was generated in situ by pre-mixing allylpalladium(II) chloride dimer with **6q** in THF at ambient temperature for 30 min. The palladium-to-ligand ratios were 2/1 and 1/2. To our delight, bis(phosphane) **6q** proved to be suitable for the asymmetric allylic alkylation reaction, giving the alkylated product in excellent yields and with very good enantioselectivity (up to 90% ee). The results of the optimisation study show that the phosphine palladium complex obtained in the reaction with stoichiometry P/Pd = 1/1 was more beneficial in terms of the stereoselectivity of the reaction (Table 7, entry 1). Thus, it was proven that, in a benchmark asymmetric allylic alkylation reaction, the catalyst derived from the new *R,R-***6q** phosphane was slightly more efficient than parent NORPHOS.

The enantiomeric composition and *S-* absolute configuration of the product of the model asymmetric reaction were determined by the peak integration and elution order from chiral HPLC using a Chiralcel OD-H column [37].

## 3. Materials and Methods

### 3.1. General Information

Unless specified otherwise, all starting materials and solvents were used as obtained from commercial suppliers without further purification. Organic solvents used in this study were dried over appropriate drying agents and distilled prior to use. Thin layer chromatography (TLC) was carried out using Merck silica gel 60 F_254_ plates (Merck, Kenilworth, NJ, USA). Visualisation of the TLC plates was performed by UV light, either KMnO_4_ or I_2_ stains. The NMR spectra were recorded on a Bruker Avance 500 MHz spectrometer, while chemical shifts are reported in ppm and calibrated to the residual solvent peaks at 7.27 ppm and 77.00 ppm for ^1^H and ^13^C, respectively, in CDCl_3_ or internal reference compounds. A similar technique was applied recently for the structural analysis of many norbornene systems [39,40]. The following abbreviations are used in reporting NMR data: s (singlet), d (doublet), t (triplet), q (quartet), m (multiplet), br (broad). Coupling constants (J) are given in Hz. The spectra are reported as follows: chemical shift (δ, ppm), multiplicity, integration, coupling constants (Hz). The IR spectra were recorded on a Nicolet 8700A FTIR-ATR spectrometer; wave numbers are in cm^-1^. Products were purified by flash chromatography on silica gel 60 (230–400 mesh) using a BUCHI chromatograph. The X-ray diffraction intensities were collected at room temperature on a SuperNova X-ray diffractometer equipped with an Atlas S2 CCD detector using mirror-monochromatised CuKα radiation (λ = 1.54184 Å). Low- and high-resolution mass spectra were obtained with a Shimadzu LC-MS (Kinetex^®^ 2.6 µm Biphenyl 100 Å 50 × 2.1 mm LC-column, acetonitrile/water with the HCO_2_H additive mobile phase) IT-TOF spectrometer. Commercially unavailable substrates were obtained by known literature procedures. The physical properties and spectra of the obtained products are available free of charge in the Supplementary Data.

### 3.2. Synthesis and Spectral Data

#### 3.2.1. Typical Procedure for the Coupling Reaction Leading to the Formation of [(1*R*,2*R*,9*S*,10*S*,11*R*,12*R*)-4-phenyltetracyclo[8.2.1.0^2,9^.0^3,8^]trideca-3,5,7-triene-11,12-diyl]bis(diphenylphosphane) dioxide (*R,R-***4q**)

*(R,R)*-**1** (1.0g, 1.54 mmol), **2q** (1.84 mmol), Cs_2_CO_3_ (4.63 mmol) and Pd(PPh_3_)_4_ (0.07 mmol) were placed in a Schlenk tube under argon. Dry DMF was added (15 mL) and the mixture was stirred at 105 °C for 18–24 h. Then, the mixture was cooled down to root temperature (RT). The solvent was evaporated under reduced pressure, and 50 mL of water was added. The products were extracted in DCM (3 × 30 mL). The combined organic phase was dried over MgSO_4_, filtered and the solvent was evaporated. The product was purified by silica-gel column chromatography (Hexane/*i*PrOH 50/1) and finally crystallised from the mixture of hexane/DCM. The compound was isolated in 98% yield as a white solid, m.p.: 255–258 °C, [α${]}_{D}^{20}$ = +79.4 (*c* = 0.45, CHCl_3_), ^31^P-NMR (202 MHz, CDCl_3_): δ = 32.5 (d, *J* = 10.1), 28.7 (d, *J* = 10.1), ^1^H-NMR (500 MHz, CDCl_3_): δ = 8.68–8.65 (m, 2H, Ar-H), 7.99–7.42 (m, 20H, Ar-H), 7.13–7.06 (m, 5H, Ar-H), 6.83 (d, 1H, *J* = 10.0, Ar-H), 4.29 (s, 1H, CH), 3.93–3.83 (m, 2H, CH), 3.52–3.47 (m, 1H, CH), 2.61 (d, 2H, *J* = 5.0, CH), 1.90 (d, 1H, *J* = 10.0, CH), 1.01–0.85 (m, 1H, CH), ^13^C-NMR (125 MHz, CDCl_3_): δ = 145.3, 141.8, 137.3, 134.1, 133.4, 132.1, 132.0, 131.9, 131.2, 130.9, 130.6, 130.5, 130.4, 130.1, 130.0, 128.8, 128.7, 128.5, 128.5, 128.5, 128.4, 128.3, 128.2, 127.1, 127.0, 126.8, 126.5, 126.1, 125.1, 120.7, 50.0, 49.9, 48.3 (d, *J* = 6.2), 41.8, 40.4, 38.4, 37.5, 32.8 (d, *J* = 12.5), LCMS (ESI) [M + H]^+^ = 647 Da, HRMS calcd. for C_43_H_36_O_2_P_2_ 647.2259, found 647.2263 (diff. 1.18 ppm).

#### 3.2.2. 1,2,3,4,4a,12b-Hexahydro-1,4-methanotriphenylene-2,3-diylbis(diphenylphosphane) dioxide (*rac-***3a**)

The compound was isolated in 69% yield as a white solid. The analysis of the product spectra confirmed its identity with literature data [12].^31^P-NMR (202 MHz, CDCl_3_): δ = 31.60 (d, *J* = 10.1), 28.78 (d, *J* = 10.1), ^1^H-NMR (500 MHz, CDCl_3_): δ = 7.98–7.96 (m, 2H, Ar-H), 7.86–7.85 (m, 2H, Ar-H), 7.76–7.72 (m, 2H, Ar-H), 7.64–7.50 (m, 10H, Ar-H), 7.17–7.05 (m, 10H, Ar-H), 6.81–6.78 (m, 2H, Ar-H), 5.22 (d, 1H, *J* = 2.0, Ar-H), 4.00 (d, 1H, *J* = 4.0, CH), 3.95–3.87 (m, 1H, CH-P), 3.86–3.80 (m, 1H, CH-P), 3.71 (d, 1H, *J* = 4.0, CH), 2.64 (s, 1H, CH), 2.45 (d, 1H, *J* = 4.0, CH_2_), 1.98 (d, 1H, *J* = 4.0, CH_2_), 1.45–1.44 (m, 1H, CH).

#### 3.2.3. (5-Methyltetracyclo[8.2.1.0^2,9^.0^3,8^]trideca-3,5,7-triene-11,12-diyl)bis(diphenylphosphane) dioxide (*rac*-**4b**)

The compound was isolated in 83% yield as a white solid, m.p.: 208–213 °C. ^31^P-NMR (202 MHz, CDCl_3_): δ = 32.4 (d, *J* = 8.08), 28.7 (d, *J* = 6.06), ^1^H-NMR (500 MHz, CDCl_3_): δ = 7.87–7.76 (m, 15H, Ar-H), 7.11–6.55 (m, 8H, Ar-H), 3.93 (s, 1H, CH), 3.87–3.83 (m, 1H, CH), 3.56 (s, 1H, CH), 3.31–3.27 (m, 1H, CH), 2.34 (d, 1H, *J* = 10.0, CH_2_), 2.24 (s, 3H, CH_3_), 2.03 (s, 1H, CH), 1.84 (d, 1H, *J* = 10.0, CH_2_), 1.00–0.95 (m, 1H, CH), ^13^C-NMR (125 MHz, CDCl_3_): δ = 144.0 (d, *J* = 3.7), 142.0, 137.1, 134.3, 135.1, 134.4, 134.0, 133.6, 133.3, 132.6, 131.8, 131.2, 131.0, 130.8, 130.5, 130.4, 130.2, 130.1, 129.8, 128.7, 128.6, 128.5, 128.4, 128.3, 128.2, 128.2, 122.6, 121.4, 50.0 (d, *J* = 15.0), 45.2 (d, *J* = 6.2), 41.6 (d, *J* = 6.2), 38.3, 37.8, 32.5 (d, *J* = 12.5), 21.9 LCMS (ESI) [M + H]^+^ = 585 Da, HRMS calcd. for C_38_H_34_O_2_P_2_ 585.2119, found 585.2108 (diff. 1.18 ppm).

#### 3.2.4. (4-Methoxytetracyclo[8.2.1.0^2,9^.0^3,8^]trideca-3,5,7-triene-11,12-diyl)bis(diphenylphosphane) dioxide (*rac*-**4c**)

The compound was isolated in 76% yield as a white solid, m.p.: 276–278 °C. ^31^P-NMR (202 MHz, CDCl_3_): δ = 32,4 (s), 28.3 (s), ^1^H-NMR (500 MHz, CDCl_3_): δ = 7.87–7.79 (m, 4H, Ar-H), 7.55–7.42 (m, 11H, Ar-H), 7.07–7.00 (m, 7H, Ar-H), 6.56 (d, 1H, *J* = 5.0, Ar-H), 6.45 (d, 1H, *J* = 5.0, Ar-H), 4.08 (s, 1H, CH), 3.92–3.78 (m, 1H, CH), 3.59 (s, 1H, CH), 3.38–3.30 (m, 1H, CH), 2.93 (s, 3H, OCH_3_), 2.58 (s, 1H, CH), 2.42 (s, 1H, CH), 1.99–1.97 (m, 1H, CH), 2.31 (s, 3H, CH_3_), 2.27 (s, 3H, CH_3_), 2.11–2.09 (m, 1H, CH), 1.15 (s, 1H, CH), ^13^C-NMR (125 MHz, CDCl_3_): δ = 153.2, 146.6, 131.4, 131.3, 131.0, 130.8, 130.5, 130.0, 1329., 128.8, 128.5, 128.4, 128.3, 126.6, 115.6, 114.5, 55.6, 53.4, 45.9, 43.4, 41.5 LCMS (ESI) [M + H]^+^ = 601 Da, HRMS calcd. for C_38_H_34_ O_3_P_2_ 601.2056, found 601.2067 (diff. 1.18 ppm).

#### 3.2.5. (4-Methyltetracyclo[8.2.1.0^2,9^.0^3,8^]trideca-3,5,7-triene-11,12-diyl)bis(diphenylphosphane) dioxide (*rac*-**4f**)

The compound was isolated in 85% yield as a white solid, m.p.: 270–275 °C. ^31^P-NMR (202 MHz, CDCl_3_): δ 32.3 (d, *J* = 8.08), 28.5 (d, *J* = 8.08), ^1^H-NMR (500 MHz, CDCl_3_): δ = 7.87–7.77 (m, 4H, Ar-H), 7.65–7.45 (m, 11H, Ar-H), 7.17–7.00 (m, 7H, Ar-H), 6.86 (d, 1H, *J* = 5.0, Ar-H), 6.65 (d, 1H, *J* = 5.0, Ar-H), 3.93 (s, 1H, CH), 3.81–3.75 (m, 1H, CH), 3.54 (s, 1H, CH), 3.34–3.30 (m, 1H, CH), 2.43 (s, 2H, CH_2_), 1.90 (d, 1H, *J* = 10.0, CH), 1.65 (s, 1H, CH_3_), 1.01–0.97 (m, 1H, CH), ^13^C-NMR (125 MHz, CDCl_3_): δ = 144.1 (d, *J* = 3.7), 143.7, 135.4, 134.7, 134.1, 133.6, 133.4, 132.4, 131.7, 131.4, 131.3, 130.9, 130.7, 130.6, 130.5, 130.1, 130.0, 128.7, 128.6, 128.5, 128.3, 128.3, 128.3, 128.2, 128.2, 127.9, 49.9 (d, *J* = 3.7), 45.5, 41.3, 40.9, 38.4, 37.8, 32.6 (d, *J* = 13.7), 15.7 LCMS (ESI) [M + H]^+^ = 585 Da, HRMS calcd. for C_38_H_34_ O_2_P_2_ 585.2119, found 585.2107 (diff. 1.18 ppm).

#### 3.2.6. (4-Chlorotetracyclo[8.2.1.0^2,9^.0^3,8^]trideca-3,5,7-triene-11,12-diyl)bis(diphenylphosphane) dioxide (*rac*-**4g**)

The compound was isolated in 54% yield as a white solid, m.p.: 250–254 °C. ^31^P-NMR (202 MHz, CDCl_3_): δ = 32.2 (d, *J* = 6.06), 28.7 (d, *J* = 8.08), ^1^H-NMR (500 MHz, CDCl_3_): δ = 7.87–7.73 (m, 4H, Ar-H), 7.67–7.46 (m, 11H, Ar-H), 7.17–7.03 (m, 7H, Ar-H), 6.77 (d, 1H, *J* = 10.0, Ar-H), 4.03 (s, 1H, CH), 3.83–3.77 (m, 1H, CH), 3.58 (s, 1H, CH), 3.33–3.29 (m, 1H, CH), 2.41 (s, 2H, CH_2_), 1.93 (d, 1H, *J* = 10.0, CH), 1.65 (s, 1H, CH), 0.98–0.95 (m, 1H, CH), ^13^C-NMR (125 MHz, CDCl_3_): δ = 145.1 (d, *J* = 3.7), 142.6, 134.7, 134.3, 133.9, 133.7, 133.5, 133.0, 132.2, 131.4 (d, *J* = 2.5), 131.3 (d, *J* = 2.5), 131.2 (d, *J* = 2.5), 130.9 (d, *J* = 2.5), 130.6, 130.5, 130.4, 130.4, 129.4, 129.3, 128.7, 128.7, 128.6, 128.5, 128.3, 127.5, 127.4, 126.9, 126.8, 120.5, 50.0 (d, *J* = 2.5), 49.3(d, *J* = 15), 45.7 (d, *J* = 6.2), 41.3(d, *J* = 3.7), 40.4, 38.2, 37.6, 32.6 (d, *J* = 12.5) LCMS (ESI) [M + H]^+^ = 605 Da, HRMS calcd. for C_37_H_31_O_2_P_2_Cl 605.1561, found 605.1561 (diff. 1.18 ppm).

#### 3.2.7. (4,6-Dimethyl-5-nitrotetracyclo[8.2.1.0^2,9^.0^3,8^]trideca-3,5,7-triene-11,12-diyl)bis(diphenylphosphane) dioxide (*rac*-**4h**)

The compound was isolated in 86% yield as a white solid, m.p.: 302–304 °C. ^31^P-NMR (202 MHz, CDCl_3_): δ = 32.3 (d, *J* = 8.08), 28.6 (d, *J* = 10.0), ^1^H-NMR (500 MHz, CDCl_3_): δ = 7.88–7.76 (m, 4H, Ar-H), 7.64–7.47 (m, 11H, Ar-H), 7.16–7.01 (m, 5H, Ar-H), 6.65 (s, 1H, Ar-H), 3.94 (d, 1H, *J* = 5.0, CH), 3.84–3.78 (m, 1H, CH), 3.54 (d, 1H, *J* = 5.0, CH), 3.34–3.30 (m, 1H, CH), 2.41 (d, 2H, *J* = 10.0, CH_2_), 2.10 (s, 3H, CH_3_), 1.97 (d, 1H, *J* = 10.0, CH), 1.60 (s, 1H, CH_3_), 1.00–0.95 (m, 1H, CH), ^13^C-NMR (125 MHz, CDCl_3_): δ = 151.5, 145.0 (d, *J* = 3.7), 142.4, 135.1, 134.3, 134.1, 133.6, 133.5, 132.8, 132.0, 131.5 (d, *J* = 2.5), 131.4 (d, *J* = 2.5), 131.2 (d, *J* = 2.5), 130.9 (d, *J* = 2.5), 130.6, 130.5, 130.4, 130.0, 129.8, 128.7, 128.7, 128.5, 128.4, 128.3, 128.3, 128.2, 123.8, 122.7, 49.3 (d, *J* = 15.0), 45.2(d, *J* = 6.2), 45.7 (d, *J* = 6.2), 41.3, 40.4, 38.3, 37.7, 32.6(d, *J* = 12.5) LCMS (ESI) [M + H]^+^ = 644 Da, HRMS calcd. for C_39_H_35_NO_4_P_2_ 644.2120, found 644.2114 (diff. 1.18 ppm).

#### 3.2.8. (5-Methoxytetracyclo[8.2.1.0^2,9^.0^3,8^]trideca-3,5,7-triene-11,12-diyl)bis(diphenylphosphane) dioxide (*rac*-**4i**)

The compound was isolated in 18% yield as a white solid, m.p.: 279–285 °C. ^31^P-NMR (202 MHz, CDCl_3_): δ = 32.4(d, *J* = 8.08), 28.8 (d, *J* = 10.0), ^1^H-NMR (500 MHz, CDCl_3_): δ = 7.87–7.76 (m, 15H, Ar-H), 7.12–6.47 (m, 8H, Ar-H), 4.03 (d, 1H, *J* = 35.0, CH), 3.88–3.83 (m, 1H, CH), 3.70 (s, 1H, OCH_3_), 3.59 (d, 1H, *J* = 35.0, CH), 3.31–3.27 (m, 1H, CH), 2.49–2.33 (m, 2H, CH_2_), 1.00–0.95 (m, 1H, CH), ^13^C-NMR (125 MHz, CDCl_3_): δ = 159.7, 145.3, 134.3, 133.9, 133.5, 133.2, 132.9, 132.5, 132.1, 132.0, 131.9 (d, *J* = 6.2), 131.1, 131.6, 130.5, 130.4, 130.2, 130.1, 128.7, 128.6, 128.5, 128.4, 128.3 (d, *J* = 2.5), 128.2 (d, *J* = 2.5), 127.6, 127.4, 122.8, 122.01, 114.2, 55.3, 50.4 (d, *J* = 13.5), 49.8 (d, *J* = 13.5), 46.7, 45.7, 38.5, 37.8, 31.4, 29.7 LCMS (ESI) [M + H]^+^ = 601 Da, HRMS calcd. for C_38_H_34_O_3_P_2_ 601.2067, found 601.2056 (diff. 1.18 ppm).

#### 3.2.9. [Methyl 11,12-bis(diphenylphosphoryl)tetracyclo[8.2.1.0^2,9^.0^3,8^]trideca-3,5,7-triene-5-carboxylate (*rac*-**4j**)

The compound was isolated in 84% yield as a white solid, m.p.: 169–171 °C. ^31^P-NMR (202 MHz, CDCl_3_): δ = 32.7 (d, *J* = 8.08), 29.1 (d, *J* = 8.08), ^1^H-NMR (500 MHz, CDCl_3_): δ = 7.87–7.76 (m, 15H, Ar-H), 7.12–6.47 (m, 8H, Ar-H), 4.03 (d, 1H, *J* = 35.0, CH), 3.88–3.83 (m, 1H, CH), 3.70 (s, 1H, OCH_3_), 3.59 (d, 1H, *J* = 35.0, CH), 3.31–3.27 (m, 1H, CH), 2.49–2.33 (m, 2H, CH_2_), 1.00–0.95 (m, 1H, CH), ^13^C-NMR (125 MHz, CDCl_3_): δ = 167.3, 151.0, 144.0 (d, *J* = 3.7), 135.0, 134.2, 134.1, 133.6, 133.3, 133.3, 132.9, 132.4, 131.7, 130.5, 130.4, 130.4, 130.3, 130.2, 130.1, 129.7, 129.6, 129.9, 129.5, 128.7, 128.6, 128.5, 128.5, 128.3, 128.3, 128.3, 128.2, 123.1, 122.9, 122.0, 121.8, 51.9, 50.0 (d, *J* = 13.7), 47.9, 41.1, 38.2, 32.4 LCMS (ESI) [M + H]^+^ = 629 Da, HRMS calcd. for C_39_H_34_O_4_P_2_ 629.2022, found 629.2005 (diff. 1.7 ppm).

##### Dimethyl 2,3-bis(diphenylphosphoryl)-1,2,3,4,4a,12b-hexahydro-1,4-methanotriphenylene-7,10-dicarboxylate (*rac*-**3j**)

The compound was isolated in 51% yield as a white solid. The analysis of the product spectra confirmed its identity with literature data [12]. ^31^P-NMR (202 MHz, CDCl_3_): δ = 31.10 (d, *J* = 10.1), 28.81 (d, *J* = 8.1), ^1^H-NMR (500 MHz, CDCl_3_): δ = 8.50 (d, 1H, *J* = 10.0, Ar-H), 7.97–7.93 (m, 2H, Ar-H), 7.88–7.85 (m, 2H, Ar-H), 7.74 (d, 1H, *J* = 5.0, Ar-H), 7.74–7.48 (m, 11H, Ar-H), 7.17–7.04 (m, 6H, Ar-H), 6.86 (d, 1H, *J* = 10.0, Ar-H), 5.26 (d, 1H, *J* = 10.0, Ar-H), 4.12 (d, 1H, *J* = 10.0, CH), 3.95–3.90 (m, 1H, CH-P), 3.87–3.81 (m, 1H, CH-P), 3.93 (s, 3H, CH_3_), 3.92 (s, 3H, CH_3_), 3.75 (d, 1H, *J* = 10.0, CH), 2.64 (s, 1H, CH), 2.42 (d, 1H, *J* = 10.0, CH_2_), 2.02 (d, 1H, *J* = 10.0, CH_2_), 1.94 (s, 1H, CH). The HNMR spectrum is in agreement with the literature [12].

#### 3.2.10. [7,10-Bis(trifluoromethyl)-1,2,3,4,4a,12b-hexahydro-1,4-methanotriphenylene-2,3-diyl]bis(diphenylphosphane) dioxide (*rac*-**3k**)

The compound was isolated in a yield of up to 67% as a white solid. The analysis of the product spectra confirmed its identity with literature data [12]. ^31^P-NMR (202 MHz, CDCl_3_): δ = 31.04 (d, *J* = 8.1), 28.84 (d, *J* = 8.1), ^1^H-NMR (500 MHz, CDCl_3_): δ = 7.89–7.70 (m, 6H, Ar-H), 7.58–7.41 (m, 10H, Ar-H), 7.26 (d, 1H, *J* = 10.0, Ar-H), 7.10–6.97 (m, 7H, Ar-H), 6.81 (d, 1H, *J* = 10.0, Ar-H), 5.22 (d, 1H, *J* = 10.0, Ar-H), 4.02 (d, 1H, *J* = 10.0, CH), 3.83–3.76 (m, 1H, CH-P), 3.76–3.70 (m, 1H, CH-P), 3.67 (d, 1H, *J* = 10.0, CH), 2.54 (s, 1H, CH), 2.32 (d, 1H, *J* = 10.0, CH_2_), 1.97 (d, 1H, *J* = 10.0, CH_2_), 1.25–1.19 (m, 1H, CH). The HNMR spectrum is in agreement with the literature [12].

#### 3.2.11. [7,10-Bis(trimethylsilyl)-1,2,3,4,4a,12b-hexahydro-1,4-methanotriphenylene-2,3-diyl]bis(diphenylphosphane) dioxide (*rac*-**3l**)

The compound was isolated in a yield of 63% as a white solid. The analysis of the product spectra confirmed its identity with literature data [12]. ^31^P-NMR (202 MHz, CDCl_3_): δ = 31.12 (d, *J* = 6.1), 28.60 (d, *J* = 10.1), ^1^H-NMR (500 MHz, CDCl_3_): δ = 7.98–7.93 (m, 4H, Ar-H), 7.67–7.51 (m, 10H, Ar-H), 7.21–7.05 (m, 6H, Ar-H), 6.93 (d, 1H, *J* = 5.0, Ar-H), 6.78 (d, 1H, *J* = 10.0, Ar-H), 5.22 (d, 1H, *J* = 10.0, Ar-H), 4.03 (d, 1H, *J* = 10.0, CH), 3.94–3.89 (m, 1H, CH-P), 3.85–3.81 (m, 1H, CH-P), 3.68 (d, 1H, *J* = 10.0, CH), 2.64 (s, 1H, CH), 2.48 (d, 1H, *J* = 10.0, CH_2_), 1.96 (d, 1H, *J* = 10.0, CH_2_), 1.66 (s, 1H, CH), 0.28 (s, 9H, CH_3_), 0.26 (s, 9H, CH_3_). The HNMR spectrum is in agreement with the literature [12].

#### 3.2.12. 6b,7,8,9,10,10a-Hexahydro-7,10-methanobenzo[a]biphenylene-8,9-diylbis(diphenylphosphane) dioxide (*rac*-**4m**)

The compound was isolated in 68% yield as a white solid, m.p.: 225–227 °C. ^31^P-NMR (202 MHz, CDCl_3_): δ = 32.4 (d, *J* = 8.08), 28.7 (d, *J* = 6.06), ^1^H-NMR (500 MHz, CDCl_3_): δ = 7.96–7.48 (m, 16H, Ar-H), 7.36–6.28 (m, 4H, Ar-H), 7.19–7.03 (m, 6H, Ar-H), 6.83 (d, 1H, *J* = 10.0, Ar-H), 4.21 (d, 1H, *J* = 5.0, CH), 3.87–3.81 (m, 1H, CH), 3.59 (d, 1H, *J* = 5.0, CH), 3.42–3.39 (m, 1H, CH), 2.57 (s, 1H, CH), 2.46 (d, 1H, *J* = 15.0, CH_2_), 1.88 (d, 1H, *J* = 15.0, CH_2_), 0.90–0.85 (m, 1H, CH), ^13^C-NMR (125 MHz, CDCl_3_): δ = 145.9, 145.1, 142.6, 134.6, 134.2, 133.9, 133.7, 133.4, 133.3, 132.9, 132.2, 131.4, 131.3, 131.2, 130.9, 130.6, 130.5, 130.4, 130.4, 130.2, 130.1, 129.3, 128.8, 128.7, 128.6, 128.5, 128.3, 128.2, 127.5, 126.8, 120.5, 120.3, 49.8 (d, *J* = 15.0), 45.7 (d, *J* = 5.0), 41.3, 41.0, 40.4, 38.2, 37.7, 32.9 LCMS (ESI) [M + H]^+^ = 621 Da, HRMS calcd. for C_41_H_38_O_2_P_2_ 621.2139, found 621.2166 (diff. 1.18 ppm).

##### [5-(naphthalen-1-yl)bicyclo[2.2.1]heptane-2,3-diyl]bis(diphenylphosphane) dioxide (*rac*-**5m**)

This minor reaction product was detected in trace amounts in the reaction mixtures by means of ESI-LC HRMS. HRMS calcd. for C_41_H_36_O_2_P_2_ 623.2312, found 623.2263 (diff. 7.8 ppm).

#### 3.2.13. (7-Methoxy-1,2,3,4,4a,10b-hexahydro-1,4-methanobenzo[b]biphenylene-2,3-diyl)bis(diphenylphosphane) dioxide (*rac*-**4n**)

The compound was isolated in 53% yield as a white solid, m.p.: 209–214 °C. ^31^P-NMR (202 MHz, CDCl_3_): δ = 32.4 (d, *J* = 8.08), 28.8 (d, *J* = 8.08), ^1^H-NMR (500 MHz, CDCl_3_): δ = 7.91–7.42 (m, 16H, Ar-H), 7.28–7.02 (m, 10H, Ar-H), 4.21 (d, 1H, *J* = 5.0, CH), 3.92–3.85 (m, 1H, CH), 3.88 (s, 1H, OCH_3_), 3.75 (d, 1H, *J* = 5.0, CH), 3.38–3.35 (m, 1H, CH), 2.60 (s, 1H, CH), 2.46 (d, 1H, *J* = 15.0, CH_2_), 1.90 (d, 1H, *J* = 15.0, CH_2_), 1.08–1.05 (m, 1H, CH), ^13^C-NMR (125 MHz, CDCl_3_): δ = 156.7, 144.1, 141.8, 135.2, 135.1, 134.3, 133.9, 133.5, 133.2, 132.5, 131.7, 131.3, 131.1, 130.9, 130.6, 130.5, 130.4, 130.2, 130.1, 129.5, 129.4, 129.3, 128.7, 128.6, 128.5, 128.3, 128.2, 128.2, 120.0, 119.9, 119.0, 117.1, 106.6, 55.2, 50.3, 46.6, 42.9, 42.2, 39.9, 37.8, 32.9 LCMS (ESI) [M + H]^+^ = 651 Da, HRMS calcd. for C_42_H_36_O_3_P_2_ 651.2206, found 651.2212 (diff. 1.18 ppm).

#### 3.2.14. 8c,9,10,11,12,12a-Hexahydro-9,12-methanobenzo[3,4]cyclobuta[1,2-*l*]phenanthrene-10,11-diylbis(diphenylphosphane) dioxide (*rac*-**4p**)

The compound was isolated in 54% yield as a white solid, m.p.: 295–298 °C. ^31^P-NMR (202 MHz, CDCl_3_): δ = 32.3 (d, *J* = 8.08), 28.8 (d, *J* = 8.08), ^1^H-NMR (500 MHz, CDCl_3_): δ = 8.68–8.65 (m, 3H, Ar-H), 7.99–7.42 (m, 20H, Ar-H), 7.13–7.06 (m, 6H, Ar-H), 6.83 (d, 1H, *J* = 10.0, Ar-H), 4.29 (s, 1H, CH), 3.93–3.83 (m, 2H, CH), 3.52–3.47 (m, 1H, CH), 2.61 (d, 2H, *J* = 5.0, CH), 1.90 (d, 1H, *J* = 10.0, CH), 1.01–0.85 (m, 1H, CH), ^13^C-NMR (125 MHz, CDCl_3_): δ = 139.3, 138.3 (d, *J* = 3.7), 135.5, 134.7, 134.4, 133.9, 133.6, 133.1, 132.3, 132.1, 132.0, 131.9 (d, *J* = 3.7), 131.4, 131.1, 130.9, 130.8, 130.7, 130.5, 130.2, 130.1, 128.7, 128.6, 128.5, 128.4, 128.3, 128.3, 127.8, 127.8, 126.6 (d, *J* = 3.7), 123.7 (d, *J* = 8.7), 122.6, 122.3, 49.2 (d, *J* = 15.0), 45.1 (d, *J* = 6.2), 40.4, 41.1, 38.7, 32.5 (d, *J* = 12.5), LCMS (ESI) [M + H]^+^ = 671 Da, HRMS calcd. for C_45_H_38_O_2_P_2_ 671.2281, found 671.2263 (diff. 1.18 ppm).

##### [5-(phenanthren-9-yl)bicyclo[2.2.1]heptane-2,3-diyl]bis(diphenylphosphane) dioxide (*rac*-**5p**)

This minor reaction product was detected in trace amounts in the reaction mixtures by means of ESI-LC HRMS. HRMS calcd. for C_45_H_38_O_2_P_2_ 673.2422, found 673.2420 (diff. 0.3 ppm).

#### 3.2.15. Synthesis of [(1*R*,2*R*,9*S*,10*S*,11*R*,12*R*)-4-Phenyltetracyclo[8.2.1.0^2,9^.0^3,8^]trideca-3,5,7-triene-11,12-diyl]bis(diphenylphosphane) ((*R,R*)-**6q**)

One gram (1.0 g) of *(R,R)-***6q** (1.54 mmol) and 10 mL of dry toluene were added to a Schlenk flask and placed under a nitrogen atmosphere. Subsequently, 6 equiv. of SiHCl_3_ and then 18 equiv. of Bu_3_N were added and the mixture was heated at 110 °C. After the reaction had been completed, the mixture was cooled down and 30% NaOH solution was added drop-wise. The organic layer was dried over Na_2_SO_4_ and the crude was subjected to column chromatography with the ethyl acetate/hexane system. The phosphine was isolated as a white solid in 85% yield. [α${]}_{D}^{20}$ = −60 (*c* = 0.4; CHCl_3_); ^31^P-NMR (202 MHz, CDCl_3_): δ = 2.53, 1.10, −11.09, −13.67; ^1^H-NMR (500 MHz, CDCl_3_): δ = 7.94–7.42 (m, 16H, Ar-H), 7.23–7.02 (m, 11H, Ar-H), 6.83 (d, 1H, *J* = 5.0, Ar-H), 4.37 (s, 1H, CH), 3.99–3.93 (m, 1H, 2×CH), 3.69 (s, 1H, CH), 3.44–3.39 (m, 1H, CH), 2.70 (s, 1H, CH), 2.38 (d, 1H, *J* = 10.0, CH_2_), 1.90 (d, 1H, *J* = 10.0, CH_2_), 1.05–1.03 (m, 1H, CH), ^13^C-NMR (125 MHz, CDCl_3_): δ = 145.8, 141.8, 137.3, 134.3, 133.1, 131.3, 131.3, 130.9, 130.6, 130.5, 130.4, 130.1, 130.0, 128.6, 128.5, 128.3, 128.2, 127.2, 126.8, 126.5, 126.0, 125.1, 124.8, 120.8, 51.4 (d, *J* = 13.7), 50.0 (d, *J* = 13.7), 48.3 (d, *J* = 6.2), 45.3 (d, *J* = 6.2), 41.8, 41.6, 40.4, 38.4, 38.1, 37.8, 37.5.

#### 3.2.16. Synthesis of [Pd(**6q**)Cl_2_]

A solution of **6q** (0.1 g) in benzene (2 mL) was added to a stirred solution of [Pd(^t-^BuCN)_2_Cl_2_] (0.056 g) in 2 mL of THF. The reaction mixture was stirred overnight at ambient temperature, the volume of the solvents was reduced under reduced pressure down to about 2 mL and the precipitated complex was filtered off. The compound was isolated in 82% yield as a yellow solid. The samples for the X-ray measurements were grown from a warm ethanol/benzene mixture. [α${]}_{D}^{20}$ = −40.8 (c = 0.25, CH_2_Cl_2_); ^31^P-NMR (202 MHz, CDCl_3_): δ = 31.3, 31.2, 28.0, 27.9, 27.9, 27.8, ^1^H-NMR (500 MHz, CDCl_3_): δ = 8.08–8.04 (m, 2H, Ar-H), 8.02–7.98 (m, 2H, Ar-H), 7.93–7.90 (m, 2H, Ar-H), 7.88–7.84 (m, 2H, Ar-H), 7.68–7.65 (m, 1H, Ar-H), 7.59–7.58 (m, 2H, Ar-H), 7.54–7.48 (m, 22H, Ar-H), 7.33–7.27 (m, 3H, Ar-H), 7.21–7.12 (m, 8H, Ar-H), 7.07–7.01 (m, 13H, Ar-H), 6.85 (d, 1H, *J* = 7.3, CH), 6.55 (d, 1H, *J* = 7.3, CH), 4.12–4.11 (m, 1H, CH), 4.00–3.99 (m, 1H, CH), 3.96–3.87 (m, 1H, CH), 3.48–3.43 (m, 2H, CH), 2.66–2.66 (m, 1H, CH), 2.55–2.55 (m, 1H, CH), 2.36–2.34 (m, 1H, CH), 2.27–2.25 (m, 1H, CH), 1.77–1.75 (m, 1H, CH), 1.68–1.66 (m, 1H, CH), 0.75–0.74 (m, 2H, CH). ^13^C-NMR (dept 135, 125 MHz, CDCl_3_): δ = 132.96, 131.81, 131.69, 131.63, 131.54, 131.53, 131.48, 131.46, 131.20, 131.14, 130.90, 130.84, 130.76, 130.70, 130.53, 130.46, 130.40, 130.25, 130.21, 130.18, 130.14, 129.49, 129/42, 129.34, 129.30, 129.28, 129.18, 129.09, 129.07, 128.98, 128.64, 128.62, 128.55, 128.53, 128.02, 127.63, 126.48, 125.96, 125.34, 124.75, 121.32, 121.10, 51.52, 51.41, 49.85, 49.73, 48.35, 48.31, 46.39, 46.35, 41.86, 41.61, 41.13, 41.10, 40.36, 40.18, 40.00, 39.84, 38.77, 38.74, 38.41, 38.36, 38.33, 38.19, 38.16, 37.87, 37.81, 37.77, 37.75, 32.26, 32.23, 32.17, 32.13. LCMS (ESI) [M − Cl]^+^ = HRMS calcd. for C43H36P2ClPd 755.1010 Da, found 755.1034 Da (diff. 3.18 ppm).

#### 3.2.17. General Procedure for Allylic Alkylation Reaction

A solution of ligand **6q** (2.0 mol % or 4.0 mol %) and [Pd(η^3^-C_3_H_5_)Cl]_2_ (1.0 mol % or 2.0 mol %) was stirred in 1.0 mL of dry THF for 30 min. Then, *rac-(E*)-1,3-diphenylallyl acetate (0.5 mmol) in 1.0 mL of THF was added, followed by dimethyl malonate (1.5 mmol) and base (1.5 mmol) of BSA with 2 mol % of KOAc or 1.5 mmol of K_2_CO_3_/Cs_2_CO_3_ (1:1). After stirring for 12 h at room temperature, the solution was concentrated and purified by chromatography on silica gel eluted with hexane/EtOAc (10:1). The product was isolated as a colorless oil. ^1^H-NMR (500 MHz, CDCl_3_): δ = 7.34–7.22 (m, 10H, Ar-H), 6.50 (d, 1H, *J* = 15.0, -CH=), 6.33 (dd, 1H, *J* = 10.0, 15.0, -CH=), 4.31–4.27 (m, 1H, CH), 3.98 (d, 1H, *J* = 10.0, CH), 3.77 (s, 3H, OCH_3_), 3.54 (s, 3H, OCH_3_), HPLC analysis (Chiralcel OD-H, hexane/*iso*-PrOH, 98:2, 1.0 mL/min, 254 nm): tr(minor) = 10.9 min, tr(major) = 14.5 min.

### 3.3. Computational Studies

All DFT calculations were performed using the “Prometheus” cluster in the “Cyfronet” computational centre in Cracow. A new generation M062x [27] functional, implemented in the Gaussian 09 package (Gaussian Inc.: Wallingford, UK) [28], was used. All stationary structures have been optimised using the LANL2DZ basis set with one f function for Pd and without pseudopotential. All structures were characterised by only positive eigenvalues in their diagonalised Hessian matrices. For the optimised structures, thermochemical data for the temperature T = 298 K and pressure p = 1 atm were computed using vibrational analysis data.

### 3.4. Crystallographic Studies

An X-ray diffraction experiment for the complex [Pd(**6q**)Cl_2_] was conducted at room temperature on a SuperNova X-ray diffractometer equipped with an Atlas S2 CCD detector using mirror-monochromatized CuK*α* radiation (λ = 1.54184 Å). Multiscan absorption correction procedures were applied to the data [41]. The structure was solved by direct methods using the ShelXT [42] structure solution program with intrinsic phasing and refined with the Olex2.refine refinement package using Gauss–Newton minimisation [43]. Non-hydrogen atoms were refined anisotropically. The C-bound H atoms were positioned geometrically and refined with the ‘riding’ model. A summary of the experimental details and crystal structure refinement parameters are given in Table 2, Table 3, Table 4 and Table 5 and ESI. The experimental details and final atomic parameters have been deposited with the Cambridge Crystallographic Data Centre as supplementary material (CCDC: 1885894). Copies of the data can be obtained free of charge on request via www.ccdc.cam.ac.uk/data_request/cif, or by emailing data_request@ccdc.cam.ac.uk.

## 4. Conclusions

Herein, we have demonstrated the design and straightforward synthesis of novel highly rigid polycyclic bis(phosphanes). The X-ray analysis of the coordination compound obtained with a new ligand and dichloropalladium complex confirmed the cis, exo configuration of the bis(phosphane) ligand molecule. The five-membered metallacycle has an envelope conformation with a puckering amplitude q2 of 1.142(5) Å and a phase angle Φ2 of 153.8(1)°. The CSD search showed that the most frequent conformation for the five-membered metallacycles of transition metals and bis(phosphane) ligands was an envelope with one of the carbon atoms positioned at the flap regardless of the transition metal and phosphine substituent type, just as in the presented complex [Pd(**6q**)Cl_2_]. This stereochemical asymmetry observed in the analysed metallacycles is probably an important factor for efficient transition-metal-mediated asymmetric transformations. The comparison of the measured and calculated structures of the palladium complex and corresponding bis(phosphane) dioxides confirmed that, due to a structural rigidity, the conformation of the ligands depends only marginally on the substituents that those ligands bear in their polycyclic core. Notable efficiency, activity and selectivity of the new ligand in a model asymmetric allylic alkylation reaction have also been confirmed.

## Figures and Tables

**Table 1:**
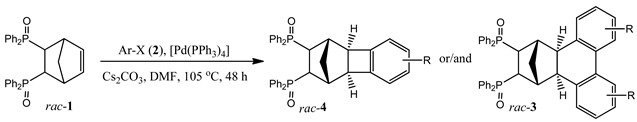
Reaction of the NORPHOS oxide with aryl halides (**2**) in the presence of [Pd(PPh_3_)_4_].

Entry	Aryl Halide	Equiv. of Aryl Halide	Product 3	Product 3 (Yield%)	Product 4	Product 4 (Yield%)
1	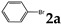	3.0	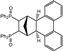	**3a** (69) ^a^	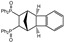	**4a** (no) ^b^
2	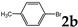	1.2 3.0	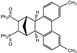	**3b** (no)	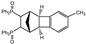	**4b** (59) **4b** (83)
3	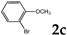	1.2	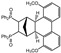	**3c** (no)	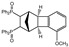	**4c** (76)
4	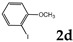	1.2 3.0	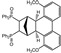	**3c** (no)	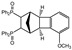	**4c** (35) **4c** (45)
5	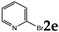	3.0	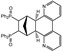	**3e** (no)	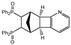	**4e** (no)
6	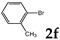	1.2 1.2 3.0	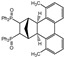	**3f** (no)	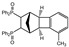	**4f** (25) ^a^ **4f** (78) **4f** (85)
7	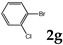	1.2 3.0	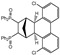	**3g** (no)	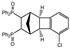	**4g** (48) **4g** (54)
8	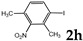	1.2	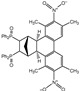	**3h** (no)	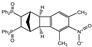	**4h** (86)
9	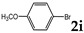	3.0	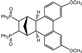	**3i** (no)	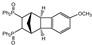	**4i** (18)
10	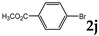	1.2 3.0	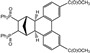	**3j** (<2) **3j** (51)	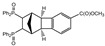	**4j** (84) **4j** (24)
11	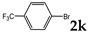	1.2 3.0	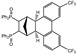	**3k** (32) **3k** (67)	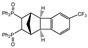	**4k** (no)
12	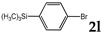	3.0	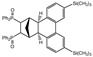	**3l** (63)	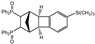	**4l** (no)
13	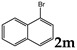	1.2	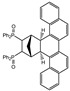	**3m** (no)	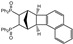	**4m** (68)
14	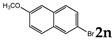	1.2	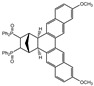	**3n** (no)	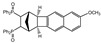	**4n** (53)
15	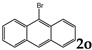	1.2	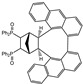	**3o** (no)	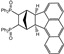	**4o** (no)
16	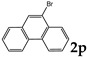	1.2	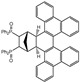	**3p** (no)	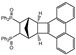	**4p** (54)
17	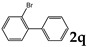	1.2	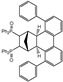	**3q** (no)	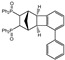	**4q** (98)

Conditions: NORPHOS oxide (0.2 mmol), aryl halide (3.0 equiv.), Cs_2_CO_3_ (3.0 equiv.), [Pd(PPh_3_)_4_] (5 mol%) and DMF (4 mL) at 105 °C for 48 h. ^a^ The reaction time was 18 h. ^b^ Product was not observed.

**Table 2:** Bond lengths (Å) and valence angles (°) for [Pd(**6q**)Cl_2_].

Bond	Length	Angle	Value
Pd1-Cl1	2.3452(12)	P1-Pd1-P1′	88.62(3)
Pd1-Cl2	2.3337(10)	P1-Pd1-Cl1	175.85(4)
Pd1-P1′	2.2463(8)	P1-Pd1-Cl2	89.30(4)
Pd1-P1	2.2722(9)	C1A-P1-Pd1′	120.58(13)
P1′-C1A	1.812(4)	C1A-P1′-C12	107.45(17)
P1′-C1B	1.802(4)	C1B-P1′-Pd1	113.58(12)
P1‘-C12	1.824(3)	C1B-P1′-C1A	105.59(18)
P1-C1C	1.820(4)	C1B-P1′-C12	108.17(16)
P1-C1D	1.811(4)	C12-P1′-Pd1	100.75(11)
P1-C11	1.836(3)	C1C-P1-Pd1	118.19(13)
C1-C2	1.537(5)	C1C-P1-C11	105.81(16)
C1-C12	1.563(4)	C1D-P1-Pd1	114.56(14)
C1-C13	1.539(4)	C1D-P1-C1C	103.96(18)
C1E-C4	1.477(6)	C1D-P1-C11	110.03(16)
C2-C3	1.525(4)	C11-P1-Pd1	103.92(11)
C2-C9	1.602(5)		
C3-C4	1.388(5)	The valence angles of the four-membered ring	
C3-C8	1.386(5)	C2-C3-C8	93.9(3)
C4-C5	1.406(5)	C3-C8-C9	94.2(3)
C5-C6	1.389(6)	C8-C9-C2	85.8(3)
C6-C7	1.394(6)	C9-C2-C3	86.1(3)
C7-C8	1.374(5)		
C8-C9	1.525(5)		
C9-C10	1.540(5)		
C10-C11	1.545(4)		
C10-C13	1.550(5)		
C11-C12	1.555(5)		

**Table 3:** Torsion angles (°) for [Pd(**6q**)Cl_2_].

Torsion	Value
Pd1-P1’-C12-C11	56.1(2)
P1’-C12-C11-P1	−61.2(2)
C12-C11-P1-Pd1	36.3(2)
C11-P1-Pd1-P1’-	−1.8(1)
P1-Pd1-P1’-C12	−26.5(1)
H11-C11-C12-H12	161
C3-C4-C1E-C2E	14.6(6)

**Table 4:** The geometry of intermolecular interactions in the [Pd(**6q**)Cl_2_] (Å,°) crystal.

D–H…A	D–H	D...A	H...A	<D–H...A
C2C-H2C…Cl2	0.93	3.482(4)	2.73	139
C6-H6…Cl2*^i^*	0.93	3.557(4)	2.87	132
C6E-H6E…Cl1*^i^*	0.93	3.623(4)	2.76	154
C7-H7…Cl1*^ii^*	0.93	3.756(4)	2.99	141
C3B-H3B…*Cg*1*^ii^*	0.93	3.689(5)	2.77	169
C5B-H5B…*Cg*2*^iii^*	0.93	3.521(4)	2.71	146

Symmetry codes: *^i^* −1 + x, y, z; *^ii^* ^−^½ + y, ½ − x, ¼ + z; *^iii^* −1 + y, 1 + x, 1 − z. *Cg*1 ring centroid C1E-C6E; *Cg*2 ring centroid C1A-C6A.

**Table 5:** Absolute values of the endo- and exocyclic torsions within the five-membered metallacycles found in the Cambridge Structural Database (CSD).

Code	Torsion	Minimum	Maximum	Mean
abs(T1 or T3)	Tr–P–C–C	0.733	60.136	36.141
abs(T2)	P–C–C–P	0.353	69.677	44.108
abs(T4 or T5)	P–Tr–P–C	0.921	44.906	17.174
abs(T6)	H–C–C–H	0.164	179.96	113.52

T1–T5, endocyclic torsion angles defined in Figure 4; Tr, transition metal.

**Table 6:**
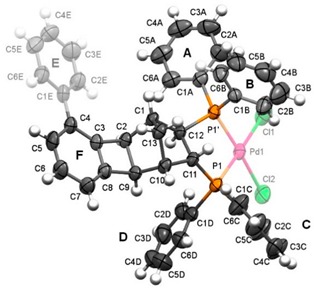
Selected geometrical parameters of experimental and calculated [Pd(**6**)Cl_2_] and **4.**

	[Pd(6q)Cl_2_]*^xray^*	[Pd(6q)Cl_2_]*^calc^*	[Pd(6a)Cl_2_]*^calc^*	4q^*calc*^	4a^*calc*^
Angle, (°)					
A/B	73.3	81.5	81.3	68.9	70.0
C/D	74.3	67.4	67.6	66.7	65.5
B/C	39.6	39.3	43.7	4.2	8.2
A/D	39.3	45.1	39.9	45.4	44.5
Torsion angle, (°)					
C3-C4-C1E-C2E	14.6	30.4	na	29.3	na
P1-C11-C12-P2	−61.2	−67.2	−66.9	−101.5	−103.8
Bond lengths, (Å)					
C1-C2	1.537(5)	1.551	1.552	1.550	1.550
C2-C3	1.525(4)	1.529	1.530	1.532	1.532
C3-C4	1.388(5)	1.397	1.390	1.397	1.391
C4-C5	1.406(5)	1.417	1.415	1.418	1.415
C5-C6	1.389(6)	1.406	1.408	1.406	1.407
C6-C7	1.394(6)	1.414	1.413	1.414	1.414
C7-C8	1.374(5)	1.390	1.392	1.391	1.391
C8-C3	1.386(5)	1.403	1.406	1.405	1.407
C8-C9	1.525(5)	1.533	1.532	1.530	1.531
C9-C2	1.602(5)	1.606	1.608	1.600	1.602
C9-C10	1.540(5)	1.549	1.550	1.547	1.546
C10-C11	1.545(4)	1.550	1.549	1.552	1.551
C11-C12	1.555(5)	1.561	1.561	1.564	1.566
C12-C1	1.563(4)	1.560	1.563	1.555	1.555
C1-C13	1.539(5)	1.552	1.552	1.549	1.550
C13-C10	1.550(5)	1.562	1.562	1.555	1.554
C12-P1	1.823(5)	1.881	1.881	1.872	1.878
C11-P2	1.836(5)	1.883	1.884	1.859	1.850
Pd-Cl1	2.345 (1)	2.423	2.425	na	na
Pd-Cl2	2.334(1)	2.420	2.419	na	na
Pd-P1	2.246 (1)	2.381	2.379	na	na
Pd-P2	2.272(1)	2.380	2.379	na	na

**Table 7:**
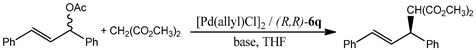
The model asymmetric allylic substitution reaction.

Entry	Ligand, mol%	[Pd(allyl)Cl]_2_, mol%	Base	Yield, %	ee, %
1	2.0 (*R,R-*6q)	2.0	BSA */KOAc	97	90 **
2	4.0 (*R,R-*6q)	1.0	BSA/KOAc	98	67
3	2.0 (*R,R-*6q)	2.0	Cs_2_CO_3_/K_2_CO_3_	97	81
4	2.0 (*R,R-*6q)	2.0	Cs_2_CO_3_	96	83
5	2.0 (*R,R-*1)	2.0	BSA/KOAc	95	87
6	2.0 (*R,R-*1)	2.0	Cs_2_CO_3_	92	85
7	1.2 (*S,S-*1)	0.5	NaH	80	81 ***

**6q** (2.0 or 4.0 mol%), [Pd(allyl)Cl]_2_ (1.0 or 2.0 mol%), THF (1.0 mL), 30 min; next 1,3-diphenylallyl acetate (0.5 mmol), THF (1.0 mL), dimethyl malonate (1.5 mmol), base (1.5 mmol), 12 h, ambient temperature. * BSA = bis(trimethylsilyl)acetamide. ** [α${]}_{D}^{20}$ = −14.8 (c = 1.3, CHCl_3_). *** Reported previously [38].

## Supplementary Materials

The NMR spectra of the obtained compounds and some additional experimental details are available online at http://www.mdpi.com/1420-3049/24/3/571/s1.
