# Supplementary material for: New Rigid Polycyclic Bis(phosphane) for Asymmetric Catalysis
Source: Molecules. 2019 Feb 5;24(3):571. doi: 10.3390/molecules24030571 (PMC6385021; doi:10.3390/molecules24030571)
Supplement: Supplementary file 1 [file molecules-24-00571-s001.pdf]

New rigid polycyclic bis(phosphane) for asymmetric catalysis

(ESI)

K. Michał Pietrusiewicz, [1] Katarzyna Szwaczko, [1*] Barbara Mirosław, [2*] Izabela Dybała, [3] Radomir Jasiński, [4] and Oleg M. Demchuk [1,5]

[1]*Department of Organic Chemistry, Faculty of Chemistry, Maria Curie-Sklodowska University, 33-Gliniana St., 20-031 Lublin, Poland; katarzyna.szwaczko@poczta.umcs.lublin.pl;*
[2]*Department of Crystallography, Faculty of Chemistry, Maria Curie-Sklodowska University, 3-Maria Curie-Sklodowska St., 20-031 Lublin, Poland; barbara.miroslaw@poczta.umcs.lublin.pl*
[3]*Faculty of Pharmacy, Medical University of Lublin, 4A-Chodźki St., 20-093 Lublin, Poland; izalis1@gazeta.pl*
[4]*Department of Organic Chemistry, Cracow University of Technology, 24-Warszawska St., 31-155 Cracow, Poland; radomir@indy.chemia.pk.edu.pl*
[5]*Pharmaceutical Research Institute, 8-Rydygiera St., 01-793 Warsaw, Poland; O.Demchuk@IFarm.eu*

Table of Contents:

1. Chemistry. General information typical procedure **s2**

2. Coupling products, synthesis and physical properties **s4**

3. Synthesis of catalyst and model catalytic reaction **s9**

4. Single-crystal X-ray analysis **s11**

5. Computational studies **s12**

6. NMR Spectra **s13**

7. References **s28**

1. CHEMISTRY. GENERAL INFORMATION AND TYPICAL PROCEDURE

General. Unless noted otherwise, all starting materials and solvents were used as obtained from commercial suppliers without further purification. Organic solvents used in this study were dried over appropriate drying agents and distilled prior to use. Thin layer chromatography was carried out using Merck silica gel 60 F_{254} plates (Merck, Kenilworth, NJ, USA). Visualisation of TLC plates was performed by UV light either $KMnO_4$ or I_2 stains. NMR spectra were recorded on Bruker Avance 500 MHz spectrometer, and chemical shifts are reported in ppm, and calibrated to residual solvent peaks at 7.27 ppm and 77.00 ppm for ^1H and ^{13}C in CDCl3 or internal reference compounds. Similar technique was applied recently for structural analysis of many norbornene systems.[1], [2] The following abbreviations are used in reporting NMR data: s (singlet), d (doublet), t (triplet), q (quartet), m (multiplet), br (broad). Coupling constants (J) are in Hz. Spectra are reported as follows: chemical shift (δ, ppm), multiplicity, integration, coupling constants (Hz). Products were purified by flash chromatography on silica gel 60 (230-400 mesh) using BUCHI chromatograph. The X-ray diffraction intensities were collected on SuperNova X-ray diffractometer equipped with Atlas S2 CCD detector using mirror-monochromatised CuKα radiation (λ = 1.54184 Å). Low- and high-resolution mass spectra were obtained with Shimadzu LC-MS (Kinetex® 2.6 µm Biphenyl 100 Å 50 x 2.1 mm LC-column, acetonitrile/water with HCO_2H additive mobile phase) IT-TOF spectrometer. Not available commercially substrates were obtained by known literature procedures. The physical properties and spectra of obtained products are available free of charge in Supplementary data.

General procedure for the coupling reaction. 1 (0.2 mmol), **2** (0.24 mmol), Cs_2CO_3 (0.6 mmol), $Pd(PPh_3)_4$ (0.01 mmol) were placed in a Schlenk tube under argon. Dry DMF (2 mL) was added and the mixture was stirred at 105 °C for 18-24 h. Then, the mixture was cooled down to RT. The DMF was evaporated under reduced pressure and 20 mL of water was added. The products were extracted with DCM (3 x 20 mL). The combined organic phase was dried over $MgSO_4$, drying agent was filtered off and DCM was evaporated. The product was purified by silica-gel column chromatography (Hexane/*i*PrOH 50/1) and finally crystallised and finally recrystallized from appropriate solvent mixture.

Table S1. Evaluation of bases suitable for the catalytic cyclization reaction.

Entry	Base	Equiv. of *p*$BrC_6H_4CH_3$ (2b)	Yield of 4b, %
1	K_3PO_4	1.2	54
2	KOAc	1.2	50
3	KO*t*Bu	1.2	39
4	K_2CO_3	1.2	43
5	Cs_2CO_3	1.2	59
		3.0	83

Conditions: NORPHOS oxide (0.2 mmol), **2b** (0.24 mmol), base (0.6 mmol), $[Pd(PPh_3)_4]$ (5 mol%), DMF (4 ml) at 105 °C for 48 h.

Table S2. Evaluation of solvent suitable for the catalytic cyclization reaction.

Entry	Base	Equiv. of *p*$BrC_6H_4CH_3$ (2b)	Yield of 4b, %
1	DMF	1.2	83
2	Toluene	1.2	79
3	THF	1.2	72
4	CH3CN	1.2	65

Conditions: NORPHOS oxide (0.2 mmol), **2b** (0.24 mmol), base (0.6 mmol), [Pd(PPh3)4] (5 mol%), solvent (4 ml) at 105 °C for 48 h.

Table S3. Effect of catalyst loading on the model catalytic cyclization reaction.

Entry	Mol % of $[Pd(PPh_3)_4]$	Base	Equiv. of *p*$BrC_6H_4CH_3$ (2b)	Yield of 4b, %
1	5.0	Cs_2CO_3	1.2	83
2	1.0	Cs_2CO_3	1.2	45
3	0.1	Cs_2CO_3	1.2	21

Conditions: NORPHOS oxide (0.2 mmol), **2b** (0.24 mmol), base (0.6 mmol), [Pd(PPh3)4], DMF (4 ml) at 105 °C for 48 h.

2. COUPLING PRODUCTS, SYNTHESIS AND PHYSICAL PROPERTIES

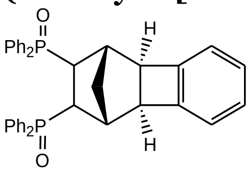

(Tetracyclo[8.2.1.0^{2,9}.0^{3,8}]trideca-3,5,7-triene-11,12-diyl)bis(diphenylphosphane) dioxide (*rac*-**4a**): This minor reaction product was detected in trace amount in the reaction mixtures by means of ESI-LC HRMS. HRMS calcd. for $C_{37}H_{32}O_2P_2$ 571.1951, found 571.1950 (diff. 0.18 ppm).

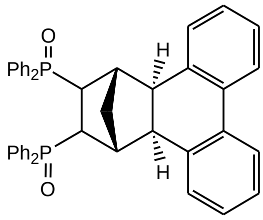

1,2,3,4,4a,12b-Hexahydro-1,4-methanotriphenylene-2,3-diylbis(diphenylphosphane) dioxide (*rac*-**3a**): The compound was isolated in 69% yield as a white solid. An analysis of spectra of the product confirms it indentity to the literature data [3]. ^{31}P NMR (202 MHz, $CDCl_3$): δ = 31.60 (d, J = 10.1), 28.78 (d, J = 10.1), ^{1}H NMR (500 MHz, $CDCl_3$): δ = 7.98-7.96 (m, 2H, Ar-H), 7.86-7.85 (m, 2H, Ar-H), 7.76-7.72 (m, 2H, Ar-H), 7.64-7.50 (m, 10H, Ar-H), 7.17-7.05 (m, 10H, Ar-H), 6.81-6.78 (m, 2H, Ar-H), 5.22 (d, 1H, J = 2.0, Ar-H), 4.00 (d, 1H, J = 4.0, CH), 3.95-3.87 (m, 1H, CH-P), 3.86-3.80 (m, 1H, CH-P), 3.71 (d, 1H, J = 4.0, CH), 2.64 (s, 1H, CH), 2.45 (d, 1H, J = 4.0, CH_2), 1.98 (d, 1H, J = 4.0, CH_2), 1.45-1.44 (m, 1H, CH).

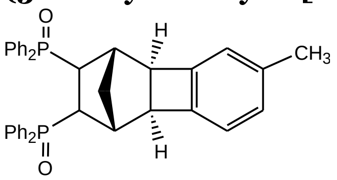

(5-Methyltetracyclo[8.2.1.0^{2,9}.0^{3,8}]trideca-3,5,7-triene-11,12-diyl)bis(diphenylphosphane) dioxide (*rac*-**4b**): The compound was isolated in 83% yield as a white solid, mp: 208-213 °C. ^{31}P NMR (202 MHz, $CDCl_3$): δ = 32.4 (d, J = 8.08), 28.7 (d, J = 6.06), ^{1}H NMR (500 MHz, $CDCl_3$): δ = 7.87-7.76 (m, 15H, Ar-H), 7.11-6.55 (m, 8H, Ar-H), 3.93 (s, 1H, CH), 3.87-3.83 (m, 1H, CH), 3.56 (s, 1H, CH), 3.31-3.27 (m, 1H, CH), 2.34 (d, 1H, J = 10.0, CH_2), 2.24 (s, 3H, CH_3), 2.03 (s, 1H, CH), 1.84 (d, 1H, J = 10.0, CH_2), 1.00-0.95 (m, 1H, CH), ^{13}C NMR (125 MHz, $CDCl_3$): δ = 144.0 (d, J = 3.7), 142.0, 137.1, 134.3, 135.1, 134.4, 134.0, 133.6, 133.3, 132.6, 131.8, 131.2, 131.0, 130.8, 130.5, 130.4, 130.2, 130.1, 129.8, 128.7, 128.6, 128.5, 128.4, 128.3, 128.2, 128.2, 122.6, 121.4, 50.0 (d, J = 15.0), 45.2 (d, J = 6.2), 41.6 (d, J = 6.2), 38.3, 37.8, 32.5 (d, J = 12.5), 21.9 LCMS (ESI) $[M+H]^+$ = 585 Da, HRMS calcd. for $C_{38}H_{34}O_2P_2$ 585.2119, found 585.2108 (diff. 1.18 ppm).

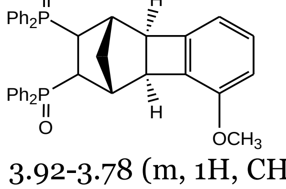

(4-Methoxytetracyclo[8.2.1.0^{2,9}.0^{3,8}]trideca-3,5,7-triene-11,12-diyl)bis(diphenylphosphane) dioxide (*rac*-**4c**): The compound was isolated in 76% yield as a white solid, mp: 276-278 °C. ^{31}P NMR (202 MHz, $CDCl_3$): δ = 32,4 (s), 28.3 (s), ^{1}H NMR (500 MHz, $CDCl_3$): δ = 7.87-7.79 (m, 4H, Ar-H), 7.55-7.42 (m, 11H, Ar-H), 7.07-7.00 (m, 7H, Ar-H), 6.56 (d, 1H, J = 5.0, Ar-H), 6.45 (d, 1H, J = 5.0, Ar-H), 4.08 (s, 1H, CH), 3.92-3.78 (m, 1H, CH), 3.59 (s, 1H, CH), 3.38-3.30 (m, 1H, CH), 2.93 (s, 3H, OCH_3), 2.58 (s, 1H, CH), 2.42 (s, 1H, CH), 1.99-1.97 (m, 1H, CH), 2.31 (s, 3H, CH_3), 2.27 (s, 3H, CH_3), 2.11-2.09 (m, 1H, CH), 1.15 (s, 1H, CH), ^{13}C NMR (125 MHz, $CDCl_3$): δ = 153.2, 146.6, 131.4, 131.3, 131.0, 130.8, 130.5, 130.0, 1329., 128.8, 128.5, 128.4, 128.3, 126.6, 115.6, 114.5, 55.6, 53.4, 45.9, 43.4, 41.5 LCMS (ESI) $[M+H]^+$ = 601 Da, HRMS calcd. for $C_{38}H_{34}\ O_3P_2$ 601.2056, found 601.2067 (diff. 1.18 ppm).

(4-Methyltetracyclo[8.2.1.0^{2,9}.0^{3,8}]trideca-3,5,7-triene-11,12-diyl)bis(diphenylphosphane) dioxide (*rac*-**4f**): The compound was isolated in 85% yield as a white solid, mp: 270-275 °C. ^{31}P NMR (202 MHz, CDCl$_3$): δ 32.3 (d, *J* = 8.08), 28.5 (d, *J* = 8.08), ^{1}H NMR (500 MHz, CDCl$_3$): δ = 7.87-7.77 (m, 4H, Ar-H), 7.65-7.45 (m, 11H, Ar-H), 7.17-7.00 (m, 7H, Ar-H), 6.86 (d, 1H, *J* = 5.0, Ar-H), 6.65 (d, 1H, *J* = 5.0, Ar-H), 3.93 (s, 1H, CH), 3.81-3.75 (m, 1H, CH), 3.54 (s, 1H, CH), 3.34-3.30 (m, 1H, CH), 2.43 (s, 2H, CH$_2$), 1.90 (d, 1H, *J* = 10.0, CH), 1.65 (s, 1H, CH$_3$), 1.01-0.97 (m, 1H, CH), ^{13}C NMR (125 MHz, CDCl$_3$): δ = 144.1 (d, *J* = 3.7), 143.7, 135.4, 134.7, 134.1, 133.6, 133.4, 132.4, 131.7, 131.4, 131.3, 130.9, 130.7, 130.6, 130.5, 130.1, 130.0, 128.7, 128.6, 128.5, 128.3, 128.3, 128.3, 128.2, 128.2, 127.9, 49.9 (d, *J* = 3.7), 45.5, 41.3, 40.9, 38.4, 37.8, 32.6 (d, *J* = 13.7), 15.7 LCMS (ESI) [M+H]$^+$ = 585 Da, HRMS calcd. for $C_{38}H_{34}O_2P_2$ 585.2119, found 585.2107 (diff. 1.18 ppm).

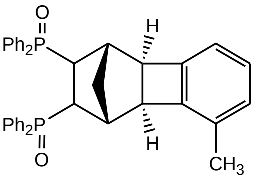

(4-Chlorotetracyclo[8.2.1.0^{2,9}.0^{3,8}]trideca-3,5,7-triene-11,12-diyl)bis(diphenylphosphane) dioxide (*rac*-**4g**): The compound was isolated in 54% yield as a white solid, mp: 250-254 °C. ^{31}P NMR (202 MHz, CDCl$_3$): δ = 32.2 (d, *J* = 6.06), 28.7 (d, *J* = 8.08), ^{1}H NMR (500 MHz, CDCl$_3$): δ = 7.87-7.73 (m, 4H, Ar-H), 7.67-7.46 (m, 11H, Ar-H), 7.17-7.03 (m, 7H, Ar-H), 6.77 (d, 1H, *J* = 10.0, Ar-H), 4.03 (s, 1H, CH), 3.83-3.77 (m, 1H, CH), 3.58 (s, 1H, CH), 3.33-3.29 (m, 1H, CH), 2.41 (s, 2H, CH$_2$), 1.93 (d, 1H, *J* = 10.0, CH), 1.65 (s, 1H, CH), 0.98-0.95 (m, 1H, CH), ^{13}C NMR (125 MHz, CDCl$_3$): δ = 145.1 (d, *J* = 3.7), 142.6, 134.7, 134.3, 133.9, 133.7, 133.5, 133.0, 132.2, 131.4 (d, *J* = 2.5), 131.3 (d, *J* = 2.5), 131.2 (d, *J* = 2.5), 130.9 (d, *J* = 2.5), 130.6, 130.5, 130.4, 130.4, 129.4, 129.3, 128.7, 128.7, 128.6, 128.5, 128.3, 127.5, 127.4, 126.9, 126.8, 120.5, 50.0 (d, *J* = 2.5), 49.3(d, *J* = 15), 45.7 (d, *J* = 6.2), 41.3(d, *J* = 3.7), 40.4, 38.2, 37.6, 32.6(d, *J* = 12.5) LCMS (ESI) [M+H]$^+$ = 605 Da, HRMS calcd. for $C_{37}H_{31}O_2P_2Cl$ 605.1561, found 605.1561 (diff. 1.18 ppm).

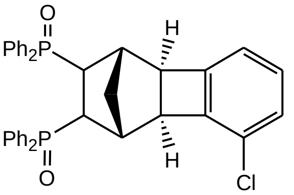

(4,6-Dimethyl-5-nitrotetracyclo[8.2.1.0^{2,9}.0^{3,8}]trideca-3,5,7-triene-11,12-diyl)bis(diphenylphosphane) dioxide (*rac*-**4h**): The compound was isolated in 86% yield as a white solid, mp: 302-304 °C. ^{31}P NMR (202 MHz, CDCl$_3$): δ = 32.3 (d, *J* = 8.08), 28.6 (d, *J* = 10.0), ^{1}H NMR (500 MHz, CDCl$_3$): δ = 7.88-7.76 (m, 4H, Ar-H), 7.64-7.47 (m, 11H, Ar-H), 7.16-7.01 (m, 5H, Ar-H), 6.65 (s, 1H, Ar-H), 3.94 (d, 1H, *J* = 5.0, CH), 3.84-3.78 (m, 1H, CH), 3.54 (d, 1H, *J* = 5.0, CH), 3.34-3.30 (m, 1H, CH), 2.41 (d, 2H, *J* = 10.0, CH$_2$), 2.10 (s, 3H, CH$_3$), 1.97 (d, 1H, *J* = 10.0, CH), 1.60 (s, 1H, CH$_3$), 1.00-0.95 (m, 1H, CH), ^{13}C NMR (125 MHz, CDCl$_3$): δ = 151.5, 145.0 (d, *J* = 3.7), 142.4, 135.1, 134.3, 134.1, 133.6, 133.5, 132.8, 132.0, 131.5 (d, *J* = 2.5), 131.4 (d, *J* = 2.5), 131.2 (d, *J* = 2.5), 130.9 (d, *J* = 2.5), 130.6, 130.5, 130.4, 130.0, 129.8, 128.7, 128.7, 128.5, 128.4, 128.3, 128.3, 128.2, 123.8, 122.7, 49.3 (d, *J* = 15.0), 45.2(d, *J* = 6.2), 45.7 (d, *J* = 6.2), 41.3, 40.4, 38.3, 37.7, 32.6(d, *J* = 12.5) LCMS (ESI) [M+H]$^+$ = 644 Da, HRMS calcd. for $C_{39}H_{35}NO_4P_2$ 644.2120, found 644.2114 (diff. 1.18 ppm).

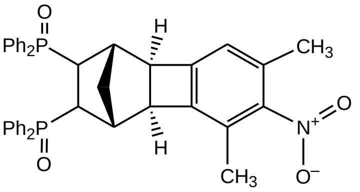

(5-Methoxytetracyclo[8.2.1.0^{2,9}.0^{3,8}]trideca-3,5,7-triene-11,12-diyl)bis(diphenylphosphane) dioxide (*rac*-**4i**): The compound was isolated in 18%yield as a white solid, mp: 279-285 °C. ^{31}P NMR (202 MHz, CDCl$_3$): δ = 32.4(d, *J* = 8.08), 28.8 (d, *J* = 10.0), ^{1}H NMR (500 MHz, CDCl$_3$): δ = 7.87-7.76 (m, 15H, Ar-H), 7.12-6.47 (m, 8H, Ar-H), 4.03 (d, 1H, *J* = 35.0, CH), 3.88-3.83 (m, 1H, CH), 3.70 (s, 1H, OCH$_3$), 3.59 (d, 1H, *J* = 35.0, CH), 3.31-3.27 (m, 1H, CH), 2.49-2.33 (m, 2H, CH$_2$), 1.00-0.95 (m, 1H, CH), ^{13}C NMR (125 MHz, CDCl$_3$): δ = 159.7, 145.3, 134.3, 133.9, 133.5, 133.2, 132.9, 132.5, 132.1, 132.0, 131.9 (d, *J* = 6.2), 131.1, 131.6, 130.5, 130.4, 130.2,

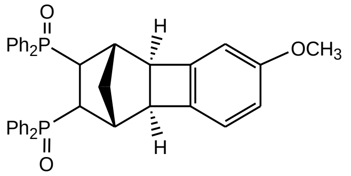

130.1, 128.7, 128.6, 128.5, 128.4, 128.3 (d, J = 2.5), 128.2 (d, J = 2.5), 127.6, 127.4, 122.8, 122.01, 114.2, 55.3, 50.4 (d, J = 13.5), 49.8 (d, J = 13.5), 46.7, 45.7, 38.5, 37.8, 31.4, 29.7 LCMS (ESI) $[M+H]^+$ = 601 Da, HRMS calcd. for $C_{38}H_{34}O_3P_2$ 601.2067, found 601.2056 (diff. 1.18 ppm).

[Methyl 11,12-bis(diphenylphosphoryl)tetracyclo[8.2.1.0^{2,9}.0^{3,8}]trideca-3,5,7-triene-5-carboxylate (*rac*-**4j**): The compound was isolated in 84% yield as a white solid, mp: 169-171 °C. ^{31}P NMR (202 MHz, $CDCl_3$): δ = 32.7 (d, J = 8.08), 29.1 (d, J = 8.08), ^{1}H NMR (500 MHz, $CDCl_3$): δ = 7.87-7.76 (m, 15H, Ar-H), 7.12-6.47 (m, 8H, Ar-H), 4.03 (d, 1H, J = 35.0, CH), 3.88-3.83 (m, 1H, CH), 3.70 (s, 1H, OCH_3), 3.59 (d, 1H, J = 35.0, CH), 3.31-3.27 (m, 1H, CH), 2.49-2.33 (m, 2H, CH_2), 1.00-0.95 (m, 1H, CH), ^{13}C NMR (125 MHz, $CDCl_3$): δ = 167.3, 151.0, 144.0 (d, J = 3.7), 135.0, 134.2, 134.1, 133.6, 133.3, 133.3, 132.9, 132.4, 131.7, 130.5, 130.4, 130.4, 130.3, 130.2, 130.1, 129.7, 129.6, 129.9, 129.5, 128.7, 128.6, 128.5, 128.5, 128.3, 128.3, 128.3, 128.2, 123.1, 122.9, 122.0, 121.8, 51.9, 50.0 (d, J = 13.7), 47.9, 41.1, 38.2, 32.4 LCMS (ESI) $[M+H]^+$ = 629 Da, HRMS calcd. for $C_{39}H_{34}O_4P_2$ 629.2022, found 629.2005(diff. 1.7 ppm).

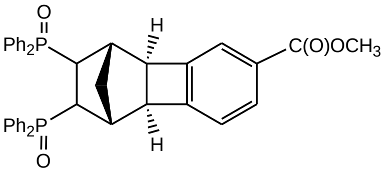

Dimethyl 2,3-bis(diphenylphosphoryl)-1,2,3,4,4a,12b-hexahydro-1,4-methanotriphenylene-7,10-dicarboxylate (*rac*-**3j**): The compound was isolated in 51% yield as a white solid. An analysis of spectra of the product confirms it indentity to the literature data [3]. ^{31}P NMR (202 MHz, $CDCl_3$): δ = 31.10 (d, J = 10.1), 28.81 (d, J = 8.1), ^{1}H NMR (500 MHz, $CDCl_3$): δ = 8.50 (d, 1H, J = 10.0, Ar-H), 7.97-7.93 (m, 2H, Ar-H), 7.88-7.85 (m, 2H, Ar-H), 7.74 (d, 1H, J = 5.0, Ar-H), 7.74-7.48 (m, 11H, Ar-H), 7.17-7.04 (m, 6H, Ar-H), 6.86 (d, 1H, J = 10.0, Ar-H), 5.26 (d, 1H, J = 10.0, Ar-H), 4.12 (d, 1H, J = 10.0, CH), 3.95-3.90 (m, 1H, CH-P), 3.87-3.81 (m, 1H, CH-P), 3.93 (s, 3H, CH_3), 3.92 (s, 3H, CH_3), 3.75 (d, 1H, J = 10.0, CH), 2.64 (s, 1H, CH), 2.42 (d, 1H, J = 10.0, CH_2), 2.02 (d, 1H, J = 10.0, CH_2), 1.94 (s, 1H, CH).

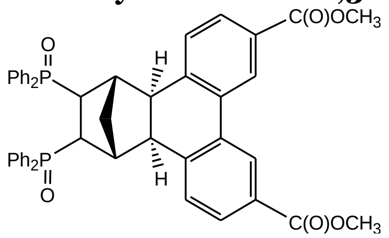

[7,10-Bis(trifluoromethyl)-1,2,3,4,4a,12b-hexahydro-1,4-methanotriphenylene-2,3-diyl]bis(diphenylphosphane) dioxide (*rac*-**3k**): The compound was isolated with yield up to 67% as a white solid. An analysis of spectra of the product confirms it indentity to the literature data [3]. ^{31}P NMR (202 MHz, $CDCl_3$): δ = 31.04 (d, J = 8.1), 28.84 (d, J = 8.1), ^{1}H NMR (500 MHz, $CDCl_3$): δ = 7.89-7.70 (m, 6H, Ar-H), 7.58-7.41 (m, 10H, Ar-H), 7.26 (d, 1H, J = 10.0, Ar-H), 7.10-6.97 (m, 7H, Ar-H), 6.81 (d, 1H, J = 10.0, Ar-H), 5.22 (d, 1H, J = 10.0, Ar-H), 4.02 (d, 1H, J = 10.0, CH), 3.83-3.76 (m, 1H, CH-P), 3.76-3.70 (m, 1H, CH-P), 3.67 (d, 1H, J = 10.0, CH), 2.54 (s, 1H, CH), 2.32 (d, 1H, J = 10.0, CH_2), 1.97 (d, 1H, J = 10.0, CH_2), 1.25-1.19 (m, 1H, CH).

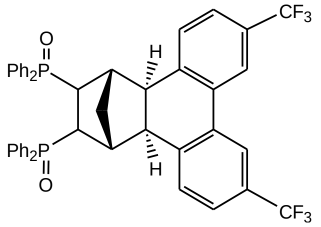

[7,10-Bis(trimethylsilyl)-1,2,3,4,4a,12b-hexahydro-1,4-methanotriphenylene-2,3-diyl]bis(diphenylphosphane) dioxide (*rac*-**3l**): The compound was isolated with yield 63% as a white solid. An analysis of spectra of the product confirms it indentity to the literature data [3]. ^{31}P NMR (202 MHz, $CDCl_3$): δ = 31.12 (d, J = 6.1), 28.60 (d, J = 10.1), ^{1}H NMR (500 MHz, $CDCl_3$): δ = 7.98-7.93 (m, 4H, Ar-H), 7.67-7.51 (m, 10H, Ar-H), 7.21-7.05 (m, 6H, Ar-H), 6.93 (d, 1H, J = 5.0, Ar-H), 6.78 (d, 1H, J = 10.0, Ar-H),

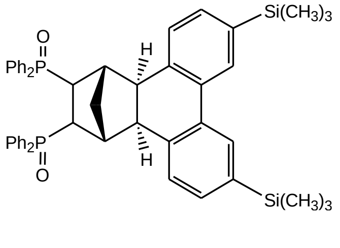

5.22 (d, 1H, J = 10.0, Ar-H), 4.03 (d, 1H, J = 10.0, CH), 3.94-3.89 (m, 1H, CH-P), 3.85-3.81 (m, 1H, CH-P), 3.68 (d, 1H, J = 10.0, CH), 2.64 (s, 1H, CH), 2.48 (d, 1H, J = 10.0, CH_2), 1.96 (d, 1H, J = 10.0, CH_2), 1.66 (s, 1H, CH), 0.28 (s, 9H, CH_3), 0.26 (s, 9H, CH_3).

6b,7,8,9,10,10a-Hexahydro-7,10-methanobenzo[a]biphenylene-8,9-diylbis(diphenylphosphane) dioxide (*rac*-**4m**): The compound was isolated in 68% yield as a white solid, mp: 225-227 °C. ^{31}P NMR (202 MHz, $CDCl_3$): δ = 32.4 (d, J = 8.08), 28.7 (d, J = 6.06), ^1H NMR (500 MHz, $CDCl_3$): δ = 7.96-7.48 (m, 16H, Ar-H), 7.36-6.28 (m, 4H, Ar-H), 7.19-7.03 (m, 6H, Ar-H), 6.83 (d, 1H, J = 10.0, Ar-H), 4.21 (d, 1H, J = 5.0, CH), 3.87-3.81 (m, 1H, CH), 3.59 (d, 1H, J = 5.0, CH), 3.42-3.39 (m, 1H, CH), 2.57 (s, 1H, CH), 2.46 (d, 1H, J = 15.0, CH_2), 1.88 (d, 1H, J = 15.0, CH_2), 0.90-0.85 (m, 1H, CH), ^{13}C NMR (125 MHz, $CDCl_3$): δ = 145.9, 145.1, 142.6, 134.6, 134.2, 133.9, 133.7, 133.4, 133.3, 132.9, 132.2, 131.4, 131.3, 131.2, 130.9, 130.6, 130.5, 130.4, 130.4, 130.2, 130.1, 129.3, 128.8, 128.7, 128.6, 128.5, 128.3, 128.2, 127.5, 126.8, 120.5, 120.3, 49.8 (d, J = 15.0), 45.7 (d, J = 5.0), 41.3, 41.0, 40.4, 38.2, 37.7, 32.9 LCMS (ESI) $[M+H]^+$ = 621 Da, HRMS calcd. for $C_{41}H_{38}O_2P_2$ 621.2139, found 621.2166 (diff. 1.18 ppm).

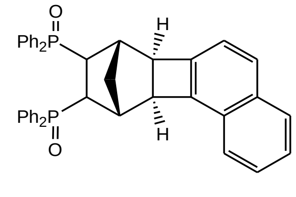

[5-(Naphthalen-1-yl)bicyclo[2.2.1]heptane-2,3-diyl]bis(diphenylphosphane) dioxide (*rac*-**5m**): This minor reaction product was detected in trace amount in the reaction mixtures by means of ESI-LC HRMS. HRMS calcd. for $C_{41}H_{36}O_2P_2$ 623.2312, found 623.2263 (diff. 7.8 ppm).

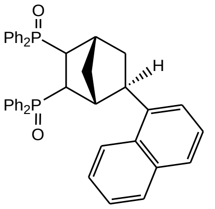

(7-Methoxy-1,2,3,4,4a,10b-hexahydro-1,4-methanobenzo[b]biphenylene-2,3-diyl)bis(diphenylphosphane) dioxide (*rac*-**4n**): The compound was isolated in 53% yield as a white solid, mp: 209-214 °C. ^{31}P NMR (202 MHz, $CDCl_3$): δ = 32.4 (d, J = 8.08), 28.8 (d, J = 8.08), ^1H NMR (500 MHz, $CDCl_3$): δ = 7.91-7.42 (m, 16H, Ar-H), 7.28-7.02 (m, 10H, Ar-H), 4.21 (d, 1H, J = 5.0, CH), 3.92-3.85 (m, 1H, CH), 3.88 (s, 1H, OCH_3), 3.75 (d, 1H, J = 5.0, CH), 3.38-3.35 (m, 1H, CH), 2.60 (s, 1H, CH), 2.46 (d, 1H, J = 15.0, CH_2), 1.90 (d, 1H, J = 15.0, CH_2), 1.08-1.05 (m, 1H, CH), ^{13}C NMR (125 MHz, $CDCl_3$): δ = 156.7, 144.1, 141.8, 135.2, 135.1, 134.3, 133.9, 133.5, 133.2, 132.5, 131.7, 131.3, 131.1, 130.9, 130.6, 130.5, 130.4, 130.2, 130.1, 129.5, 129.4, 129.3, 128.7, 128.6, 128.5, 128.3, 128.2, 128.2, 120.0, 119.9, 119.0, 117.1, 106.6, 55.2, 50.3, 46.6, 42.9, 42.2, 39.9, 37.8, 32.9 LCMS (ESI) $[M+H]^+$ = 651 Da, HRMS calcd. for $C_{42}H_{36}O_3P_2$ 651.2206, found 651.2212 (diff. 1.18 ppm).

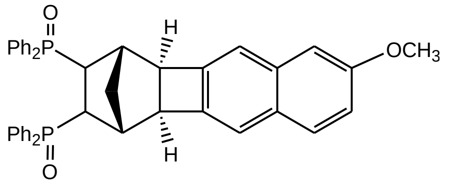

8c,9,10,11,12,12a-Hexahydro-9,12-methanobenzo[3,4]cyclobuta[1,2-*l*]phenanthrene-10,11-diylbis(diphenylphosphane) dioxide (*rac*-**4p**): The compound was isolated in 54% yield as a white solid, mp: 295-298 °C. ^{31}P NMR (202 MHz, $CDCl_3$): δ = 32.3 (d, J = 8.08), 28.8 (d, J = 8.08), ^1H NMR (500 MHz, $CDCl_3$): δ = 8.68-8.65 (m, 3H, Ar-H), 7.99-7.42 (m, 20H, Ar-H), 7.13-7.06 (m, 6H, Ar-H), 6.83 (d, 1H, J = 10.0, Ar-H), 4.29 (s, 1H, CH), 3.93-3.83 (m, 2H, 2xCH), 3.52-3.47 (m, 1H, CH), 2.61 (d, 2H, J = 5.0, CH), 1.90 (d, 1H, J = 10.0, CH), 1.01-0.85 (m, 1H, CH), ^{13}C NMR (125 MHz, $CDCl_3$): δ = 139.3, 138.3 (d, J = 3.7), 135.5, 134.7, 134.4, 133.9, 133.6, 133.1, 132.3, 132.1, 132.0, 131.9 (d, J = 3.7), 131.4, 131.1, 130.9, 130.8, 130.7, 130.5,

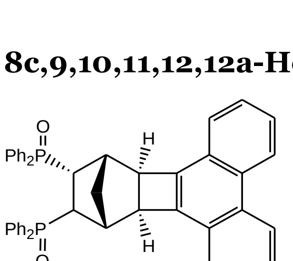

130.2, 130.1, 128.7, 128.6, 128.5, 128.4, 128.3, 128.3, 127.8, 127.8, 126.6 (d, J = 3.7), 123.7 (d, J = 8.7), 122.6, 122.3, 49.2 (d, J = 15.0), 45.1 (d, J = 6.2), 40.4, 41.1, 38.7, 32.5 (d, J = 12.5). LCMS (ESI) $[M+H]^+$ = 671 Da, HRMS calcd. for $C_{45}H_{38}O_2P_2$ 671.2281, found 671.2263 (diff. 1.18 ppm).

[5-(Phenanthren-9-yl)bicyclo[2.2.1]heptane-2,3-diyl]bis(diphenylphosphane) dioxide (*rac*-**5p**): This minor reaction product was detected in trace amount in the reaction mixtures by means of ESI-LC HRMS. HRMS calcd. for $C_{45}H_{38}O_2P_2$ 673.2422, found 673.2420 (diff. 0.3 ppm).

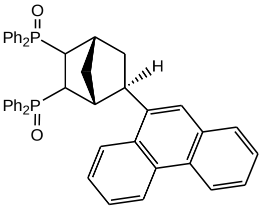

[(1*R*,2*R*,9*S*,10*S*,11*R*,12*R*)-4-Phenyltetracyclo[8.2.1.0^2,9^.0^3,8^]trideca-3,5,7-triene-11,12-diyl]bis(diphenylphosphane) dioxide (*R,R*-4q): Crystallised from a mixture of hexane/DCM. The compound was isolated in 98% yield as a white solid, mp: 255-258 °C, $[\alpha]^{20}_D$ = +79.4 (c=0.45, $CHCl_3$), ^{31}P NMR (202 MHz, $CDCl_3$): δ = 32.5 (d, J = 10.1), 28.7 (d, J = 10.1), ^1H NMR (500 MHz, $CDCl_3$): δ = 8.68-8.65 (m, 2H, Ar-H), 7.99-7.42 (m, 20H, Ar-H), 7.13-7.06 (m, 5H, Ar-H), 6.83 (d, 1H, J = 10.0, Ar-H), 4.29 (s, 1H, CH), 3.93-3.83 (m, 2H, 2xCH), 3.52-3.47 (m, 1H, CH), 2.61 (d, 2H, J = 5.0, CH), 1.90 (d, 1H, J = 10.0, CH), 1.01-0.85 (m, 1H, CH), ^{13}C NMR (125 MHz, $CDCl_3$): δ = 145.3, 141.8, 137.3, 134.1, 133.4, 132.1, 132.0, 131.9, 131.2, 130.9, 130.6, 130.5, 130.4, 130.1, 130.0, 128.8, 128.7, 128.5, 128.5, 128.5, 128.4, 128.3, 128.2, 127.1, 127.0, 126.8, 126.5, 126.1, 125.1, 120.7, 50.0, 49.9, 48.3 (d, J = 6.2), 41.8, 40.4, 38.4, 37.5, 32.8 (d, J = 12.5), LCMS (ESI) $[M+H]^+$ = 647 Da, HRMS calcd. for $C_{43}H_{36}O_2P_2$ 647.2259, found 647.2263 (diff. 1.18 ppm).

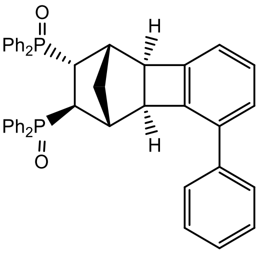

3. SYNTHESIS OF CATALYST AND MODEL CATALYTIC REACTION

Synthesis of **[(1*R*,2*R*,9*S*,10*S*,11*R*,12*R*)-4-Phenyltetracyclo[8.2.1.0^{2,9}.0^{3,8}]trideca-3,5,7-triene-11,12-diyl]bis(diphenylphosphane)** ***(R,R)-6q****:* The amount of 1.0 g of *(R,R)*-**6q** (1.54 mmol) and 10mL of dry toluene were added to a Schlenk flask and placed under a nitrogen atmosphere. Subsequently, 6 equiv. of $SiHCl_3$ and then 18 equiv. of Bu_3N were added, and the mixture was heated at 110°C. After completion of the reaction, the mixture was cooled down, and 30% NaOH solution was dropped. The organic layers were dried over Na_2SO_4 and the crude subjected to column chromatography with ethyl acetate/hexane system. The phosphine was isolated as a white solid in 85% yield. $[\alpha]^{20}_{D}$= -60 (*c*=0.4; $CHCl_3$); ^{31}P NMR (202 MHz, $CDCl_3$): δ = 2.53, 1.10, -11.09, -13.67; ^{1}H NMR (500 MHz, $CDCl_3$): δ = 7.94-7.42 (m, 16H, Ar-H), 7.23-7.02 (m, 11H, Ar-H), 6.83 (d, 1H, *J* = 5.0, Ar-H), 4.37 (s, 1H, CH), 3.99-3.93 (m, 1H, 2xCH), 3.69 (s, 1H, CH), 3.44-3.39 (m, 1H, CH), 2.70 (s, 1H, CH), 2.38 (d, 1H, *J* = 10.0, CH_2), 1.90 (d, 1H, *J* = 10.0, CH_2), 1.05-1.03 (m, 1H, CH), ^{13}C NMR (125 MHz, $CDCl_3$): δ = 145.8, 141.8, 137.3, 134.3, 133.1, 131.3, 131.3, 130.9, 130.6, 130.5, 130.4, 130.1, 130.0, 128.6, 128.5, 128.3, 128.2, 127.2, 126.8, 126.5, 126.0, 125.1, 124.8, 120.8, 51.4 (d, *J* = 13.7), 50.0 (d, *J* = 13.7), 48.3 (d, *J* = 6.2), 45.3 (d, *J* = 6.2), 41.8, 41.6, 40.4, 38.4, 38.1, 37.8, 37.5.

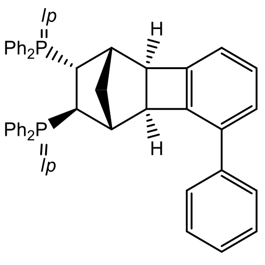

Synthesis of **[Pd(6q)Cl$_2$]**: A solution of 6q (0.1 g) in benzene (2 mL) was added to a stirred solution of $[Pd(t\text{-}BuCN)_2Cl_2]$ (0.056 g) in 2 mL of THF. The reaction mixture was stirred overnight at ambient temperature, the volume of the solvents was reduced under the reduced pressure down to about 2mL, and precipitated complex was filtered off. The compound was isolated in 82% yield as a yellow solid. The sample for the X-ray measurements were growth from warm mixture ethanol/benzene. $[\alpha]^{20}_{D}$= = -40.8 (c=0,25, CH_2Cl_2); ^{31}P NMR (202 MHz, $CDCl_3$): δ = 31.3, 31.2, 28.0, 27.9, 27.9, 27.8, ^{1}H NMR (500 MHz, $CDCl_3$): δ = 8.08-8.04 (m, 2H, Ar-H), 8.02-7.98 (m, 2H, Ar-H), 7.93-7.90 (m, 2H, Ar-H), 7.88-7.84 (m, 2H, Ar-H), 7.68-7.65 (m, 1H, Ar-H), 7.59-7.58 (m, 2H, Ar-H), 7.54-7.48 (m, 22H, Ar-H), 7.33-7.27 (m, 3H, Ar-H), 7.21-7.12 (m, 8H, Ar-H), 7.07-7.01 (m, 13H, Ar-H), 6.85 (d, 1H, *J* = 7.3, CH), 6.55 (d, 1H, *J* = 7.3, CH), 4.12-4.11 (m, 1H, CH), 4.00-3.99 (m, 1H, CH), 3.96-3.87 (m, 1H, CH), 3.48-3.43 (m, 2H, CH), 2.66-2.66 (m, 1H, CH), 2.55-2.55 (m, 1H, CH), 2.36-2.34 (m, 1H, CH), 2.27-2.25 (m, 1H, CH), 1.77-1.75 (m, 1H, CH), 1.68-1.66 (m, 1H, CH), 0.75-0.74 (m, 2H, CH). ^{13}C NMR (dept 135, 125 MHz, $CDCl_3$): δ = 132.96, 131.81, 131.69, 131.63, 131.54, 131.53, 131.48, 131.46, 131.20, 131.14, 130.90, 130.84, 130.76, 130.70, 130.53, 130.46, 130.40, 130.25, 130.21, 130.18, 130.14, 129.49, 129/42, 129.34, 129.30, 129.28, 129.18, 129.09, 129.07, 128.98, 128.64, 128.62, 128.55, 128.53, 128.02, 127.63, 126.48, 125.96, 125.34, 124.75, 121.32, 121.10, 51.52, 51.41, 49.85, 49.73, 48.35, 48.31, 46.39, 46.35, 41.86, 41.61, 41.13, 41.10, 40.36, 40.18, 40.00, 39.84, 38.77, 38.74, 38.41, 38.36, 38.33, 38.19, 38.16, 37.87, 37.81, 37.77, 37.75, 32.26, 32.23, 32.17, 32.13. LCMS (ESI) $[M - Cl]^+$ = HRMS calcd. for C43H36P2ClPd 755.1010 Da, found 755.1034 Da (diff. 3.18 ppm).

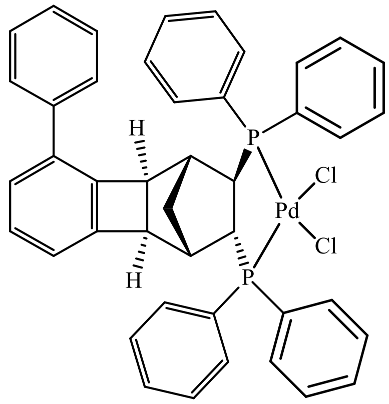

General procedure for allylic alkylation reaction: The solution of ligand **6q** (2.0 mol % or 4.0 mol %) and [Pd(η3-C_3H_5)Cl]$_2$ (1.0 mol % or 2.0 mol %) was stirred in 1.0 mL of dry THF for 30 min. Then *rac-(E)*-1,3-diphenylallyl acetate (0.5 mmol) in 1.0 mL of THF was added, followed by dimethyl malonate (1.5 mmol) and base (1.5 mmol) of BSA with 2 mol % of KOAc or 1.5 mmol of K_2CO_3/Cs_2CO_3 (1:1). After stirring for 12 h at room temperature, the solution was concentrated and purified by chromatography on silica gel eluting with hexane/EtOAc 10:1. The product was isolated as a colorless oil. ^{1}H NMR (500 MHz, $CDCl_3$): δ = 7.34-7.22 (m, 10H, Ar-H), 6.50 (d, 1H, J = 15.0, -CH=), 6.33 (dd, 1H, J = 10.0, 15.0, -CH=), 4.31-4.27 (m, 1H, CH), 3.98 (d, 1H, J = 10.0, CH), 3.77 (s, 3H, OCH_3), 3.54 (s, 3H, OCH_3). The enantiomeric composition and *S*- absolute configuration of the product of model asymmetric reaction were determined by the peak integration and an elution order from chiral HPLC Chiralcel OD-H column [4]: HPLC analysis (Chiralcel OD-H, hexane/*iso*-PrOH, 98:2, 1.0 mL/min, 254 nm): tr(minor) = 10.9 min, tr(major) = 14.5 min.

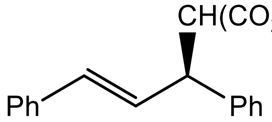

4. SINGLE-CRYSTAL X-RAY ANALYSIS

X-ray crystal structure determination of [Pd(**6q**)Cl_2]**.** The X–ray diffraction quality crystals of [Pd(**6q**)Cl_2] were grown from a solution of dichloromethane/ethanol. X-ray diffraction experiment for complex [Pd(**6q**)Cl_2] was conducted at room temperature on SuperNova X-ray diffractometer equipped with Atlas S2 CCD detector using the mirror-monochromatized Cu*Kα* radiation (λ = 1.54184 Å). Multiscan absorption correction procedures were applied to the data [5]. The structure was solved by direct methods using the ShelXT [6] structure solution program using intrinsic phasing and refined with the Olex2.refine refinement package using Gauss-Newton minimisation [7]. Non-hydrogen atoms were refined anisotropically. The C-bound H atoms were positioned geometrically and refined with the 'riding' model. The absolute structure of [Pd(**6q**)Cl_2] has been determined through the anomalous scattering with the value of the Flack parameter refined to -0.007(3) using 2963 quotients. The summary of experimental details and the crystal structure refinement parameters are given in **Table S2**. The experimental details and final atomic parameters have been deposited with the Cambridge Crystallographic Data Centre as supplementary material (CCDC: 1885894). Copies of the data can be obtained free of charge on request via www.ccdc.cam.ac.uk/data_request/cif, or by emailing data_request@ccdc.cam.ac.uk.

Table S2. Crystal data and structure refinement for [Pd(**6q**)Cl_2].

**Identification code**	[Pd(**6q**)Cl_2]
Empirical formula	$C_{43}H_{36}Cl_2P_2Pd$
Formula weight	791.96
Temperature/K	295.3(2)
Crystal system	tetragonal
Space group	$P4_32_12$
a/Å	14.08143(6)
b/Å	14.08143(6)
c/Å	37.2551(3)
Volume/Å^3	7387.18(8)
Z	8
ρ_{calc}g/cm^3	1.424
μ/mm^{-1}	6.425
F(000)	3232.0
Crystal size/mm^3	0.25 × 0.25 × 0.25
Radiation	Cu*Kα* (λ = 1.54184)
2Θ range for data collection/°	6.71 to 152.338
Index ranges	$-17 \le h \le 16, -17 \le k \le 17, -46 \le l \le 46$
Reflections collected	58744
Independent reflections	7700 [R_{int} = 0.0405, R_{sigma} = 0.0209]
Data/restraints/parameters	7700/0/433
Goodness-of-fit on F^2	1.050
Final R indexes [I>=2σ (I)]	R_1 = 0.0268, wR_2 = 0.0599
Final R indexes [all data]	R_1 = 0.0283, wR_2 = 0.0611
Largest diff. peak/hole / e Å^{-3}	0.32/-0.39
Flack parameter using 2963 quotients	-0.007(3)
CCDC No.	1885894

5. COMPUTATIONAL STUDIES.

All DFT calculations were performed using “Prometheus” cluster in the “Cyfronet” computational centre in Cracow. The new generation M062x [8] functional, implemented in the Gaussian 09 package [9] was used. All stationary structures has been optimized using LANL2DZ basis set with one f function for Pd and without of pseudopotential. All structures are characterized by only positive eigenvalues in their diagonalised Hessian matrices. For optimized structures, thermochemical data for the temperature T = 298 K and pressure p = 1 atm were computed using vibrational analysis data.

6. NMR SPECTRA

(1*R*,2*R*,3*R*,4*S*)-Bicyclo[2.2.1]hept-5-ene-2,3-diylbis(diphenylphosphane) dioxide (1).

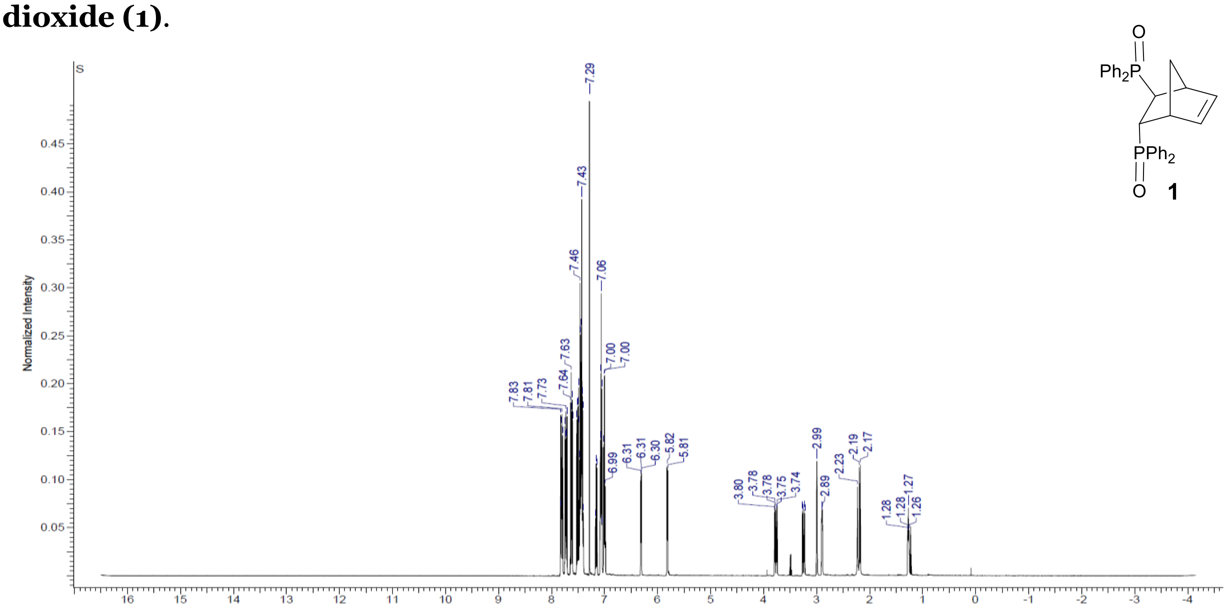

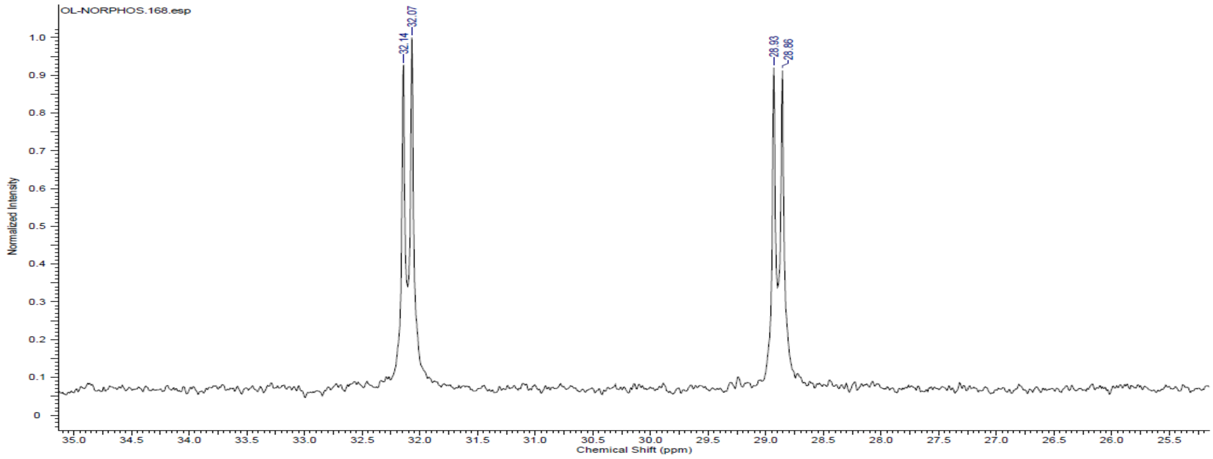

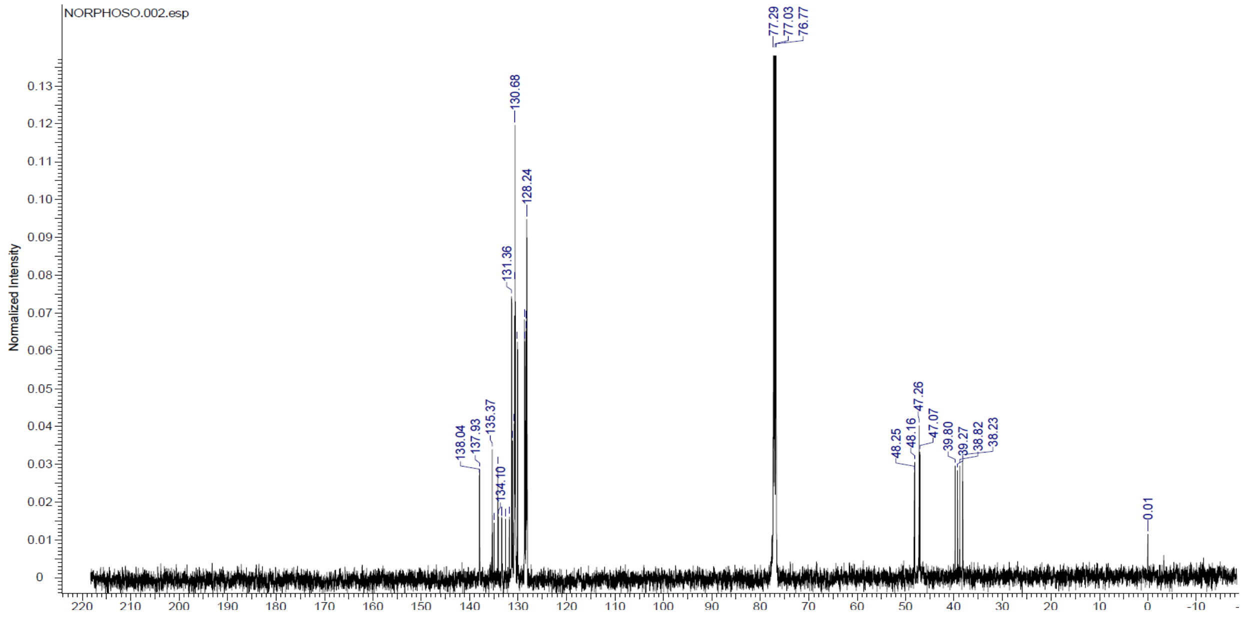

(4-Methoxytetracyclo[8.2.1.0^{2,9}.0^{3,8}]trideca-3,5,7-triene-11,12-diyl)bis(diphenylphosphane) dioxide (*rac*-4c):

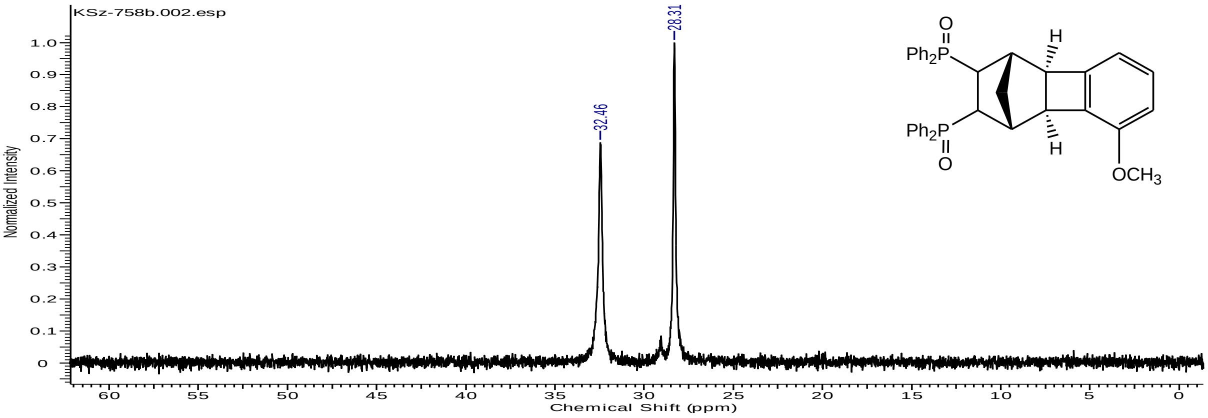

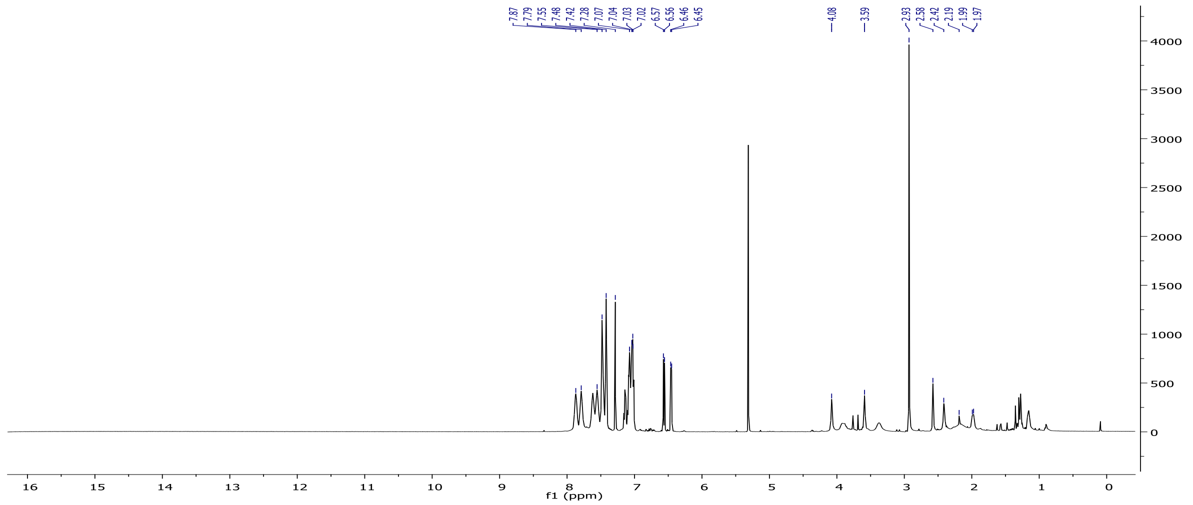

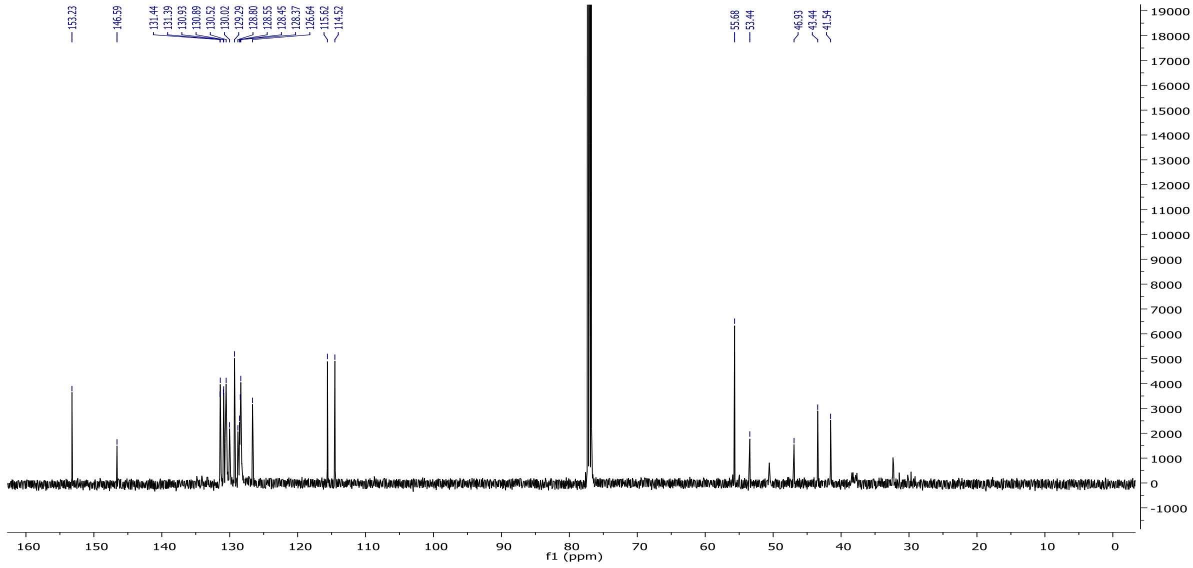

(4-Methyltetracyclo[8.2.1.0^{2,9}.0^{3,8}]trideca-3,5,7-triene-11,12-diyl)bis(diphenylphosphane) dioxide (*rac*-4f):

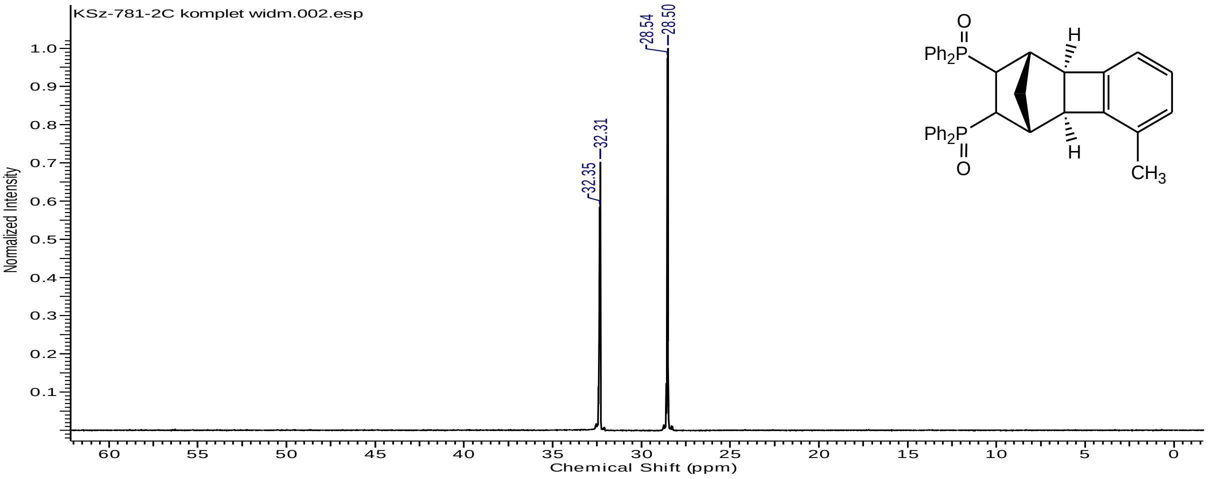

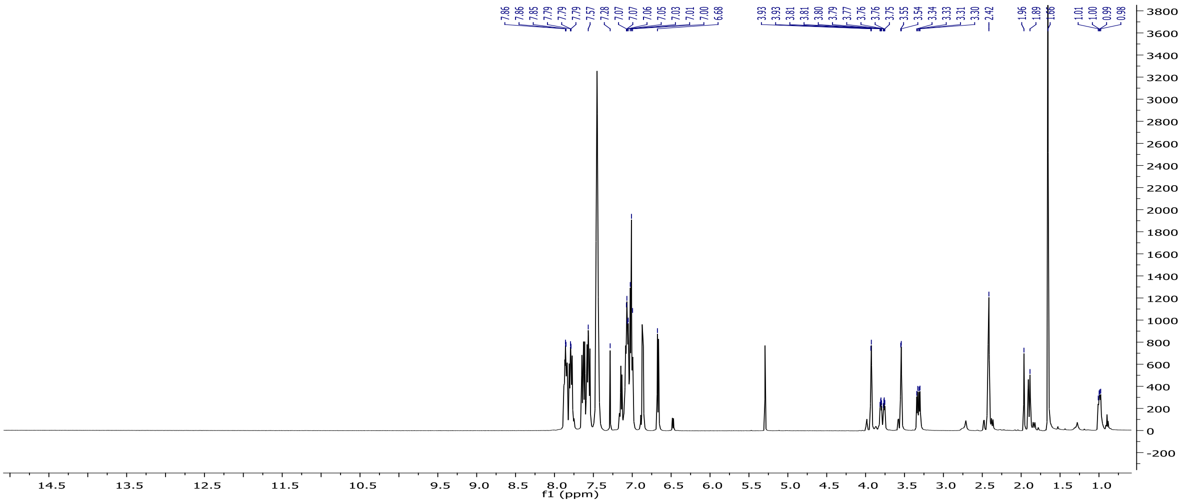

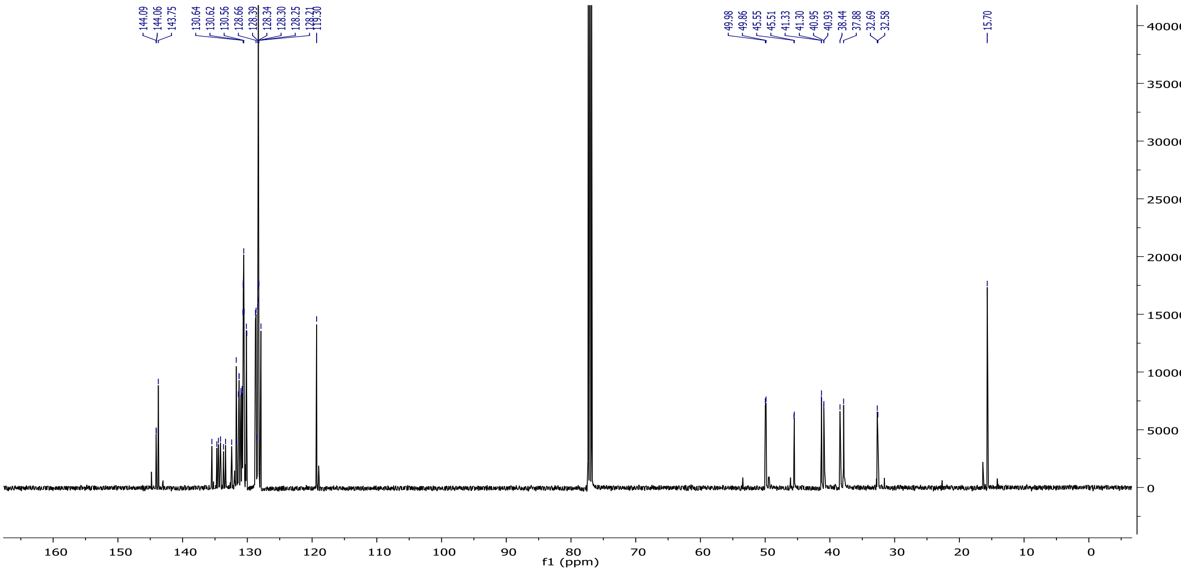

(4-Chlorotetracyclo[8.2.1.0^{2,9}.0^{3,8}]trideca-3,5,7-triene-11,12-diyl)bis(diphenylphosphane) dioxide (*rac*-4g):

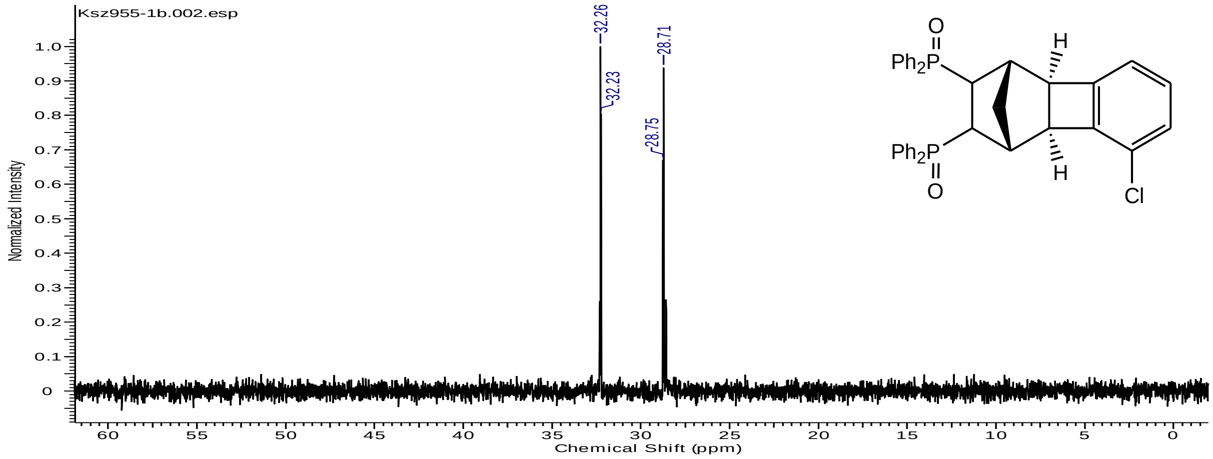

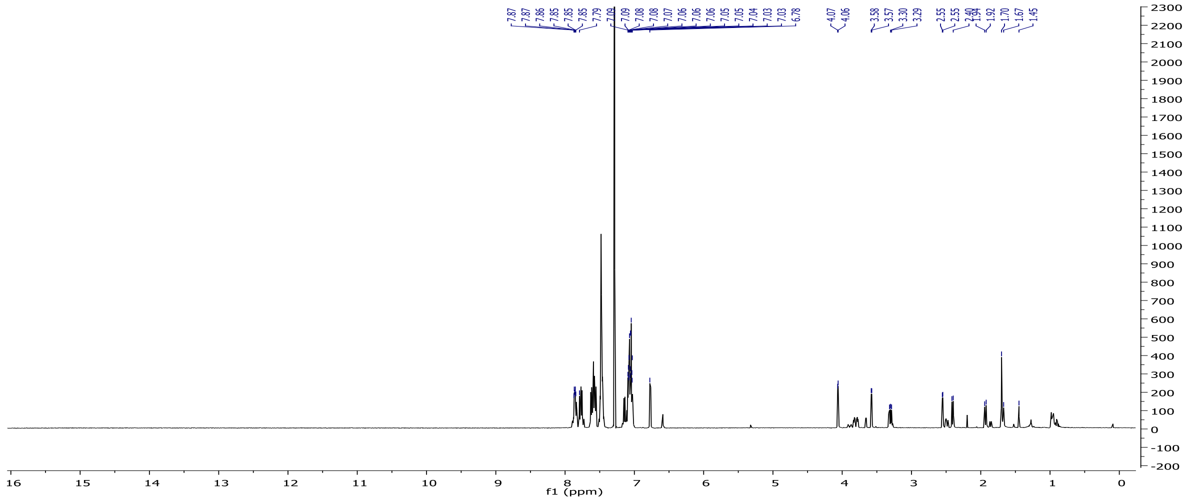

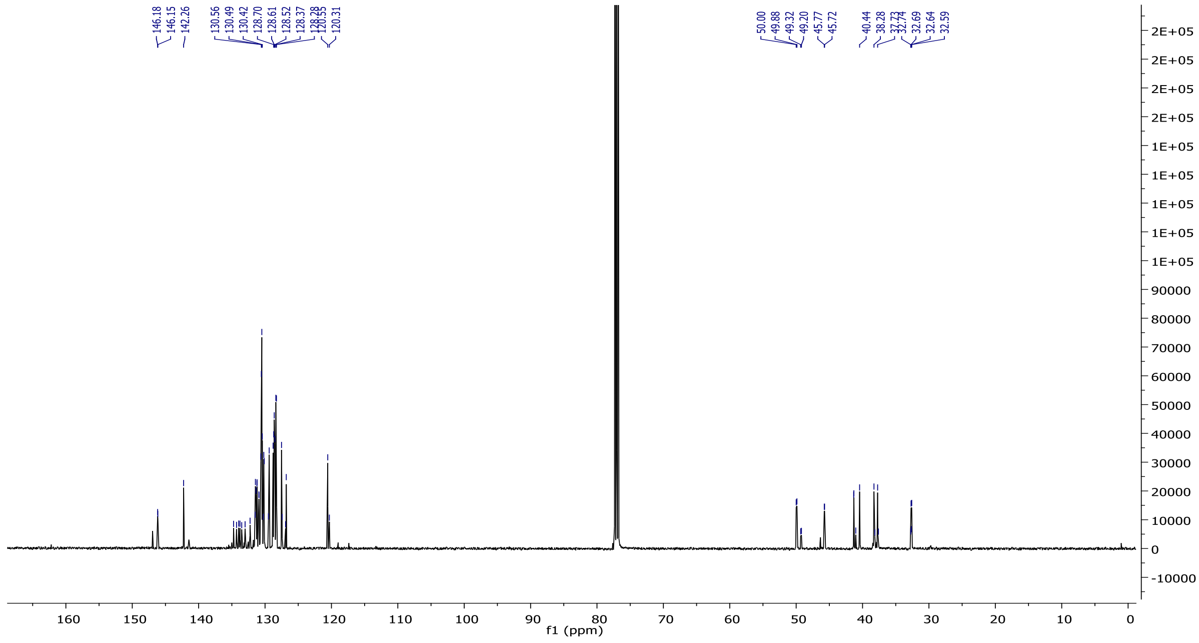

(4,6-Dimethyl-5-nitrotetracyclo[8.2.1.0^{2,9}.0^{3,8}]trideca-3,5,7-triene-11,12-diyl)bis(diphenylphosphane) dioxide (*rac*-4h):

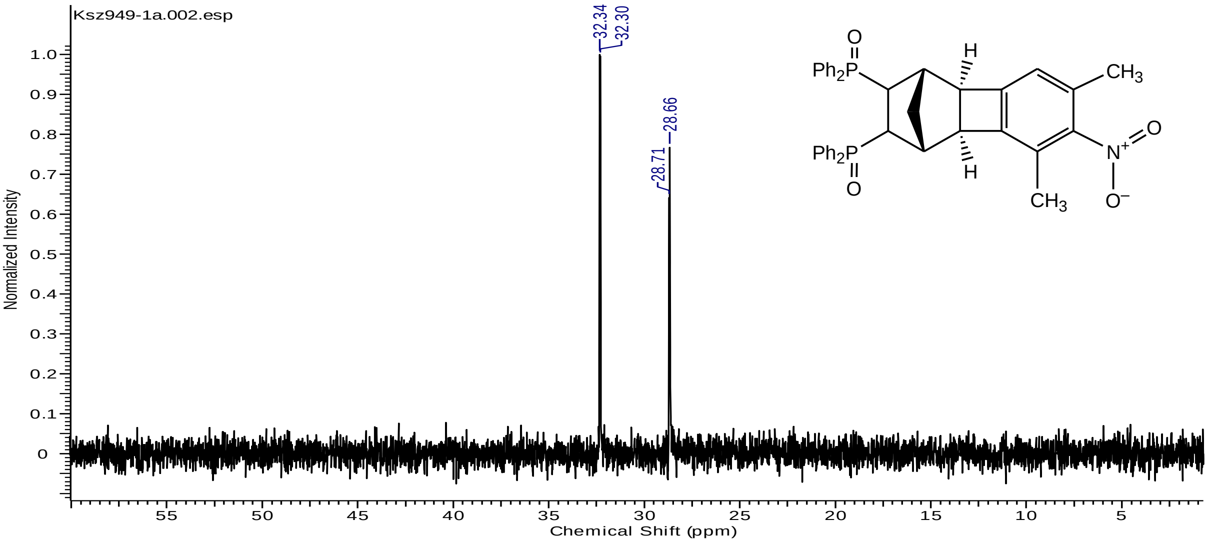

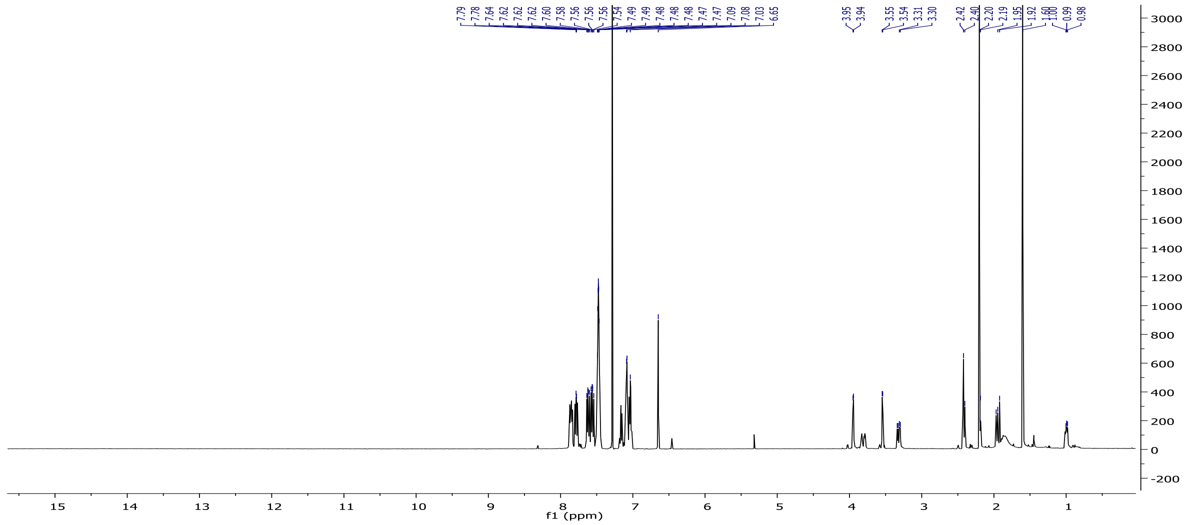

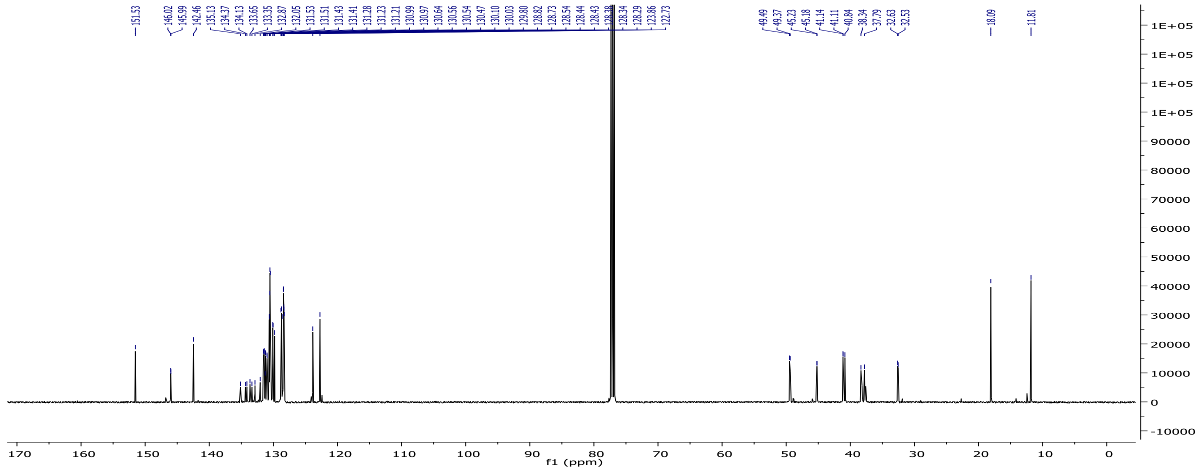

(5-Methyltetracyclo[8.2.1.0^{2,9}.0^{3,8}]trideca-3,5,7-triene-11,12-diyl)bis(diphenylphosphane) dioxide (*rac*-4b):

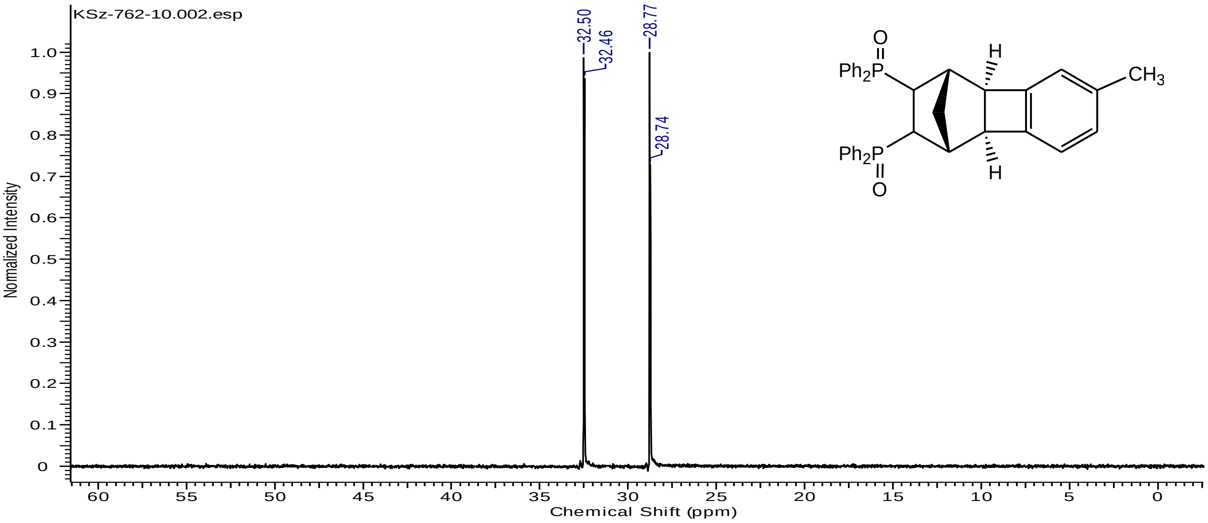

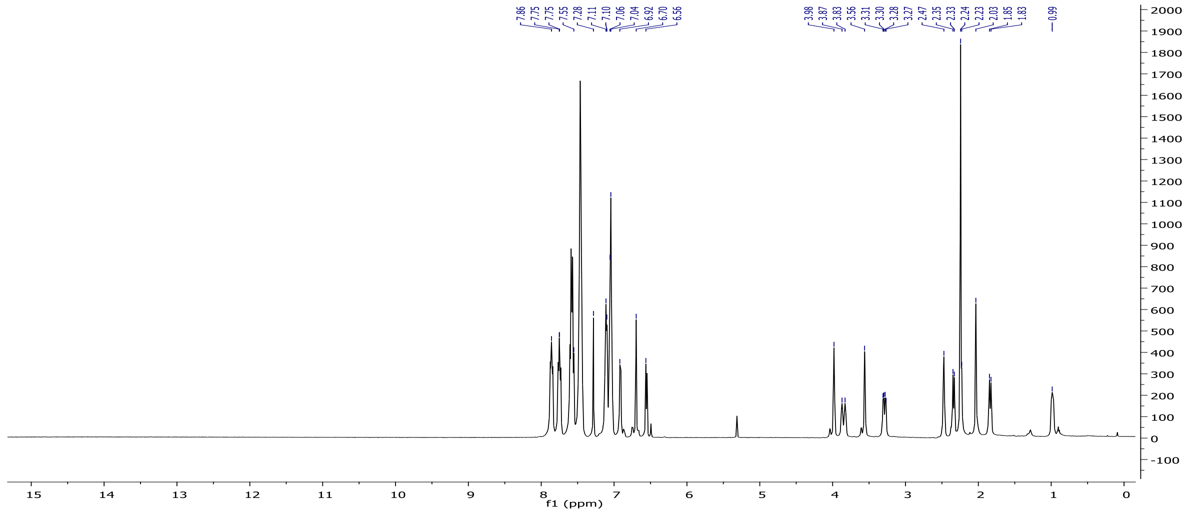

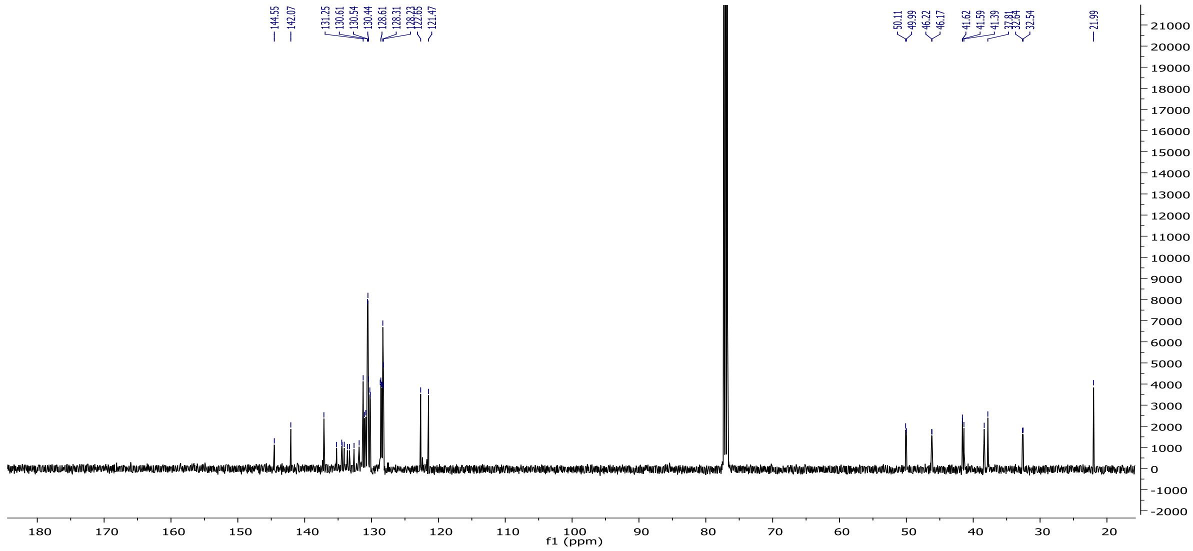

(5-Methoxytetracyclo[8.2.1.0^{2,9}.0^{3,8}]trideca-3,5,7-triene-11,12-diyl)bis(diphenylphosphane) dioxide (*rac*-4i):

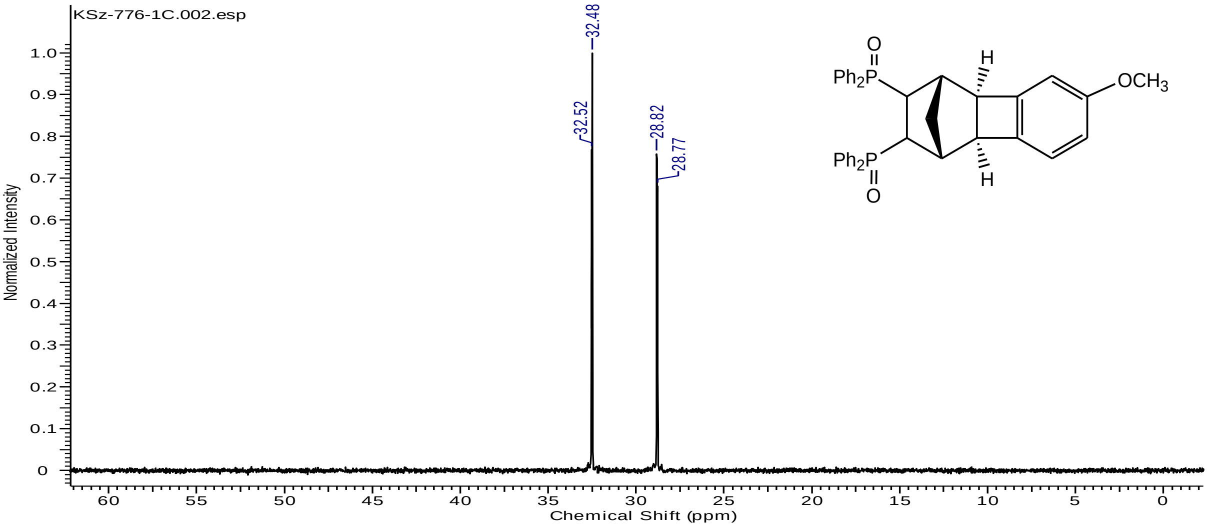

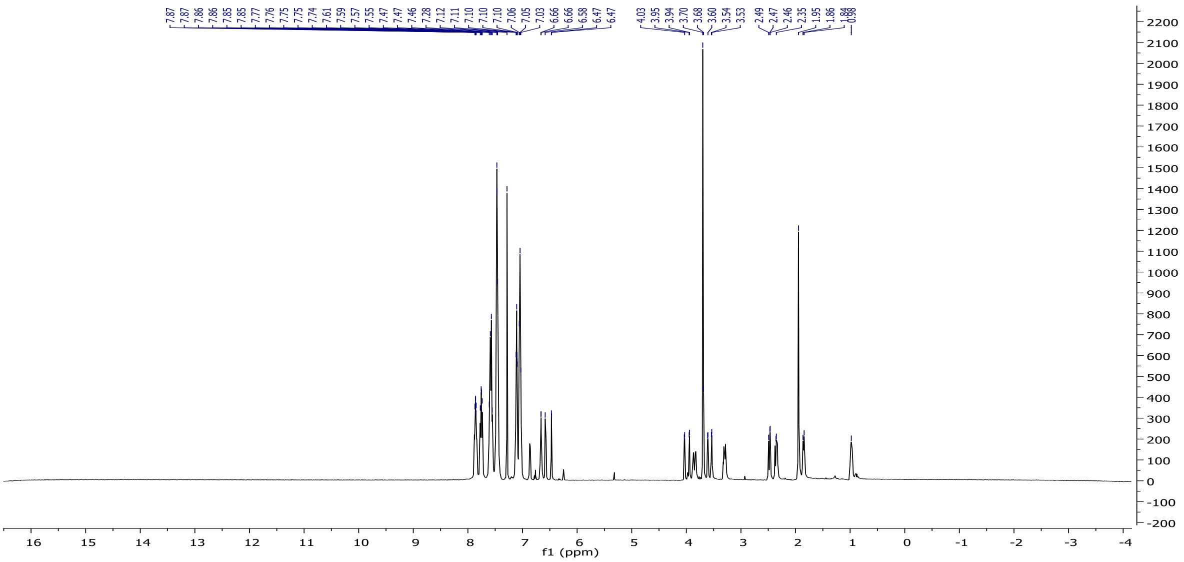

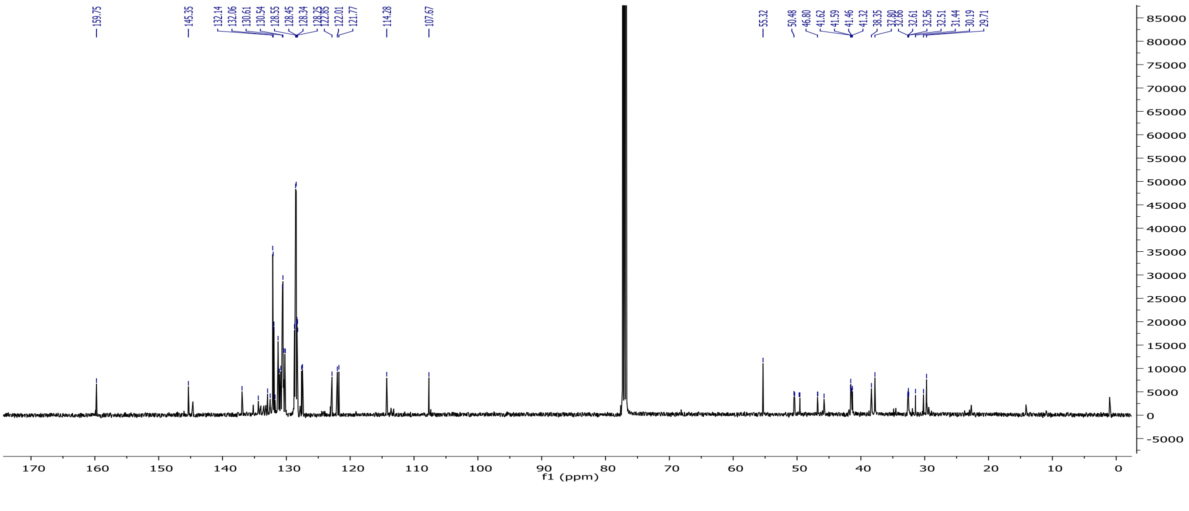

[Methyl 11,12-bis(diphenylphosphoryl)tetracyclo[8.2.1.0^{2,9}.0^{3,8}]trideca-3,5,7-triene-5-carboxylate (*rac*-4j):

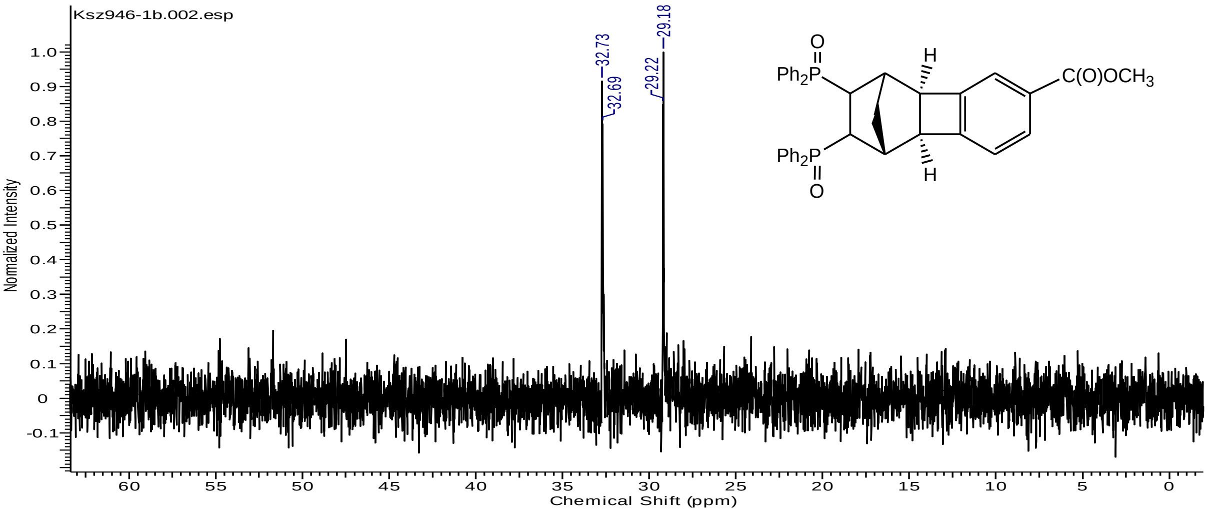

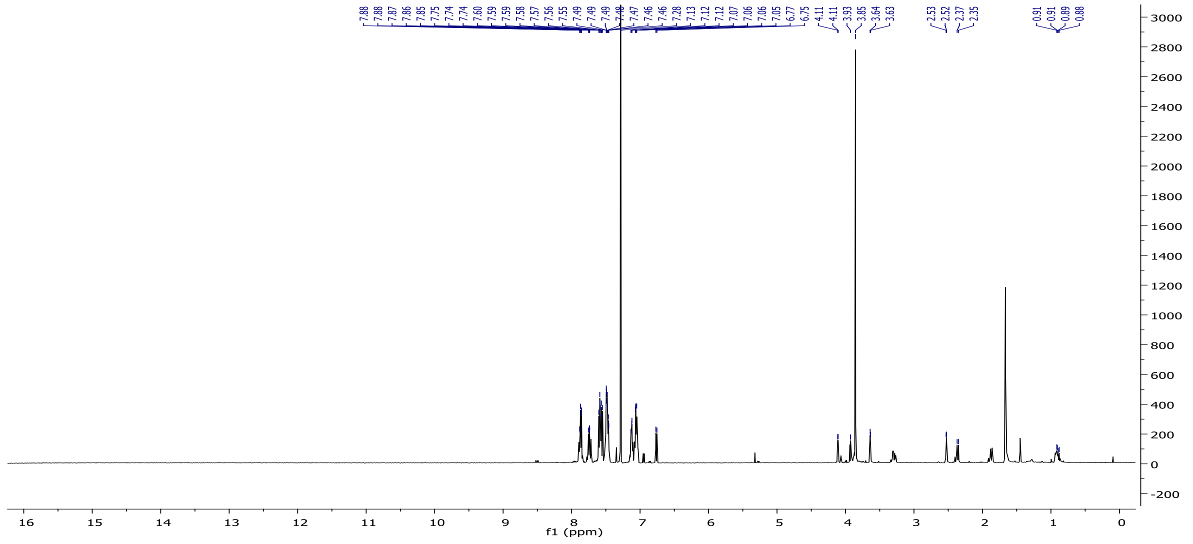

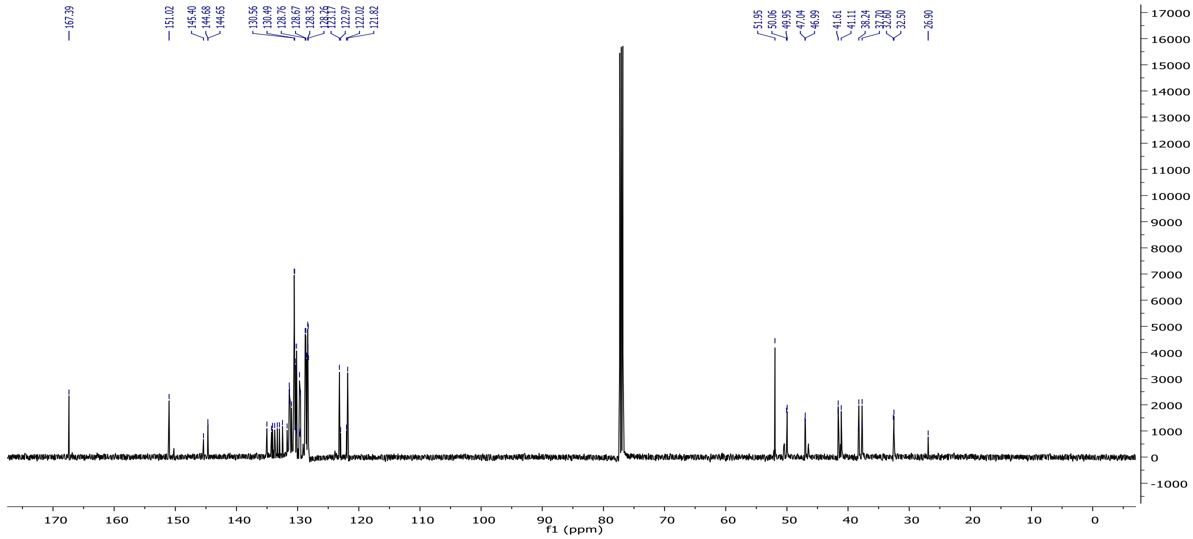

6b,7,8,9,10,10a-Hexahydro-7,10-methanobenzo[a]biphenylene-8,9-diylbis(diphenylphosphane) dioxide (*rac*-4m)

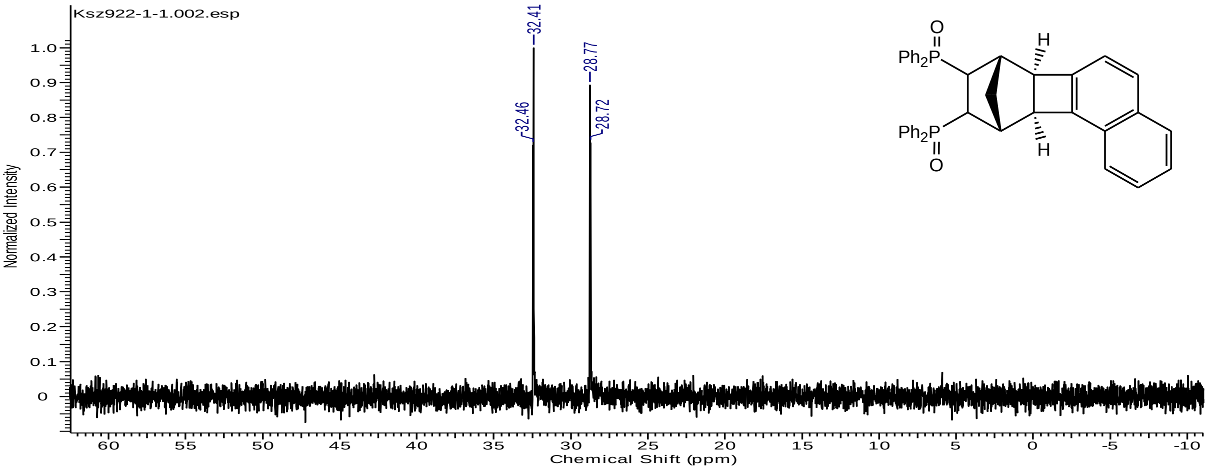

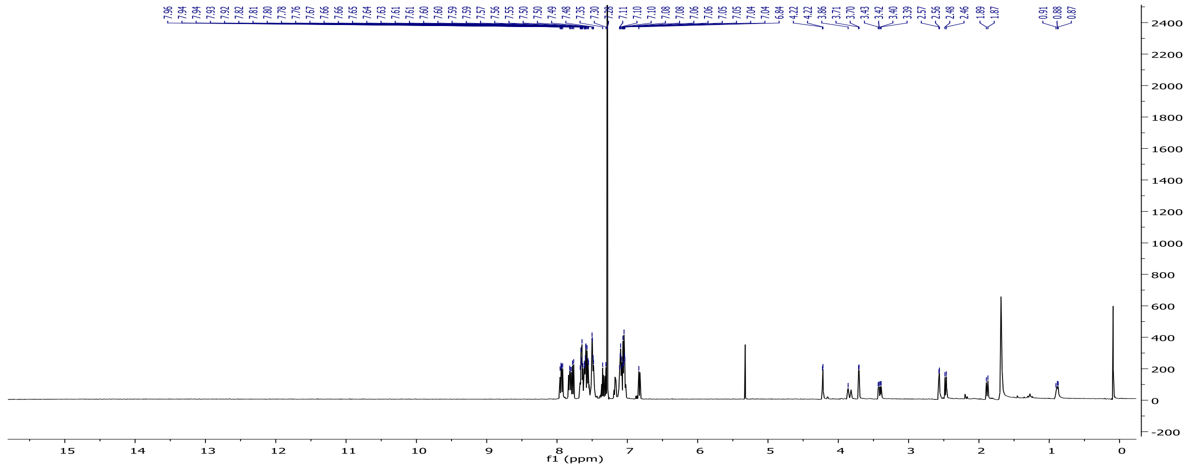

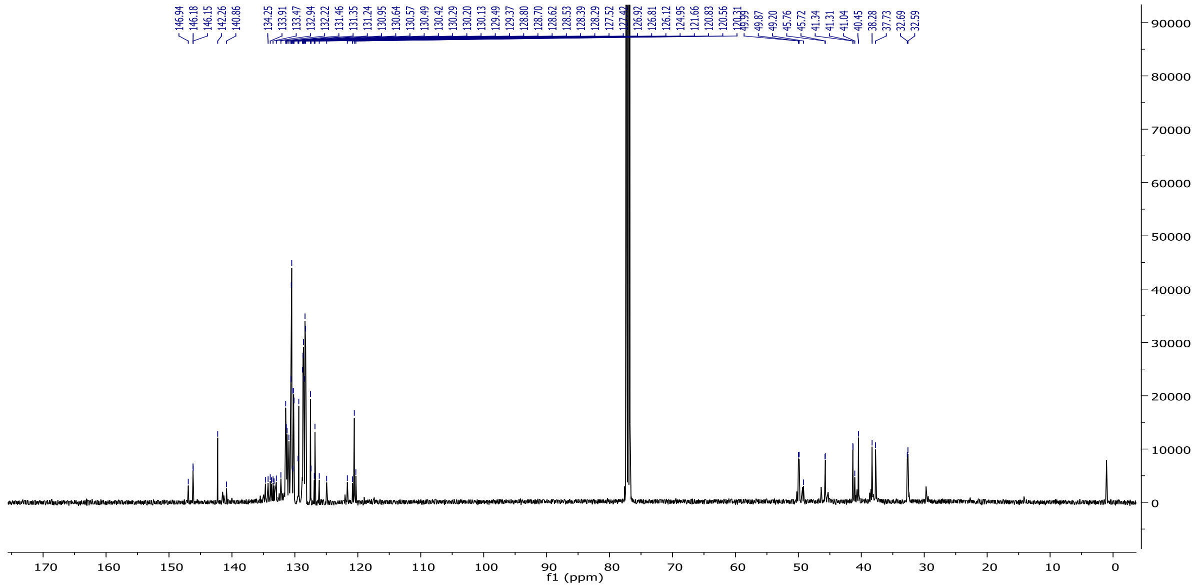

(7-Methoxy-1,2,3,4,4a,10b-hexahydro-1,4-methanobenzo[b]biphenylene-2,3-diyl)bis(diphenylphosphane) dioxide (*rac*-4n)

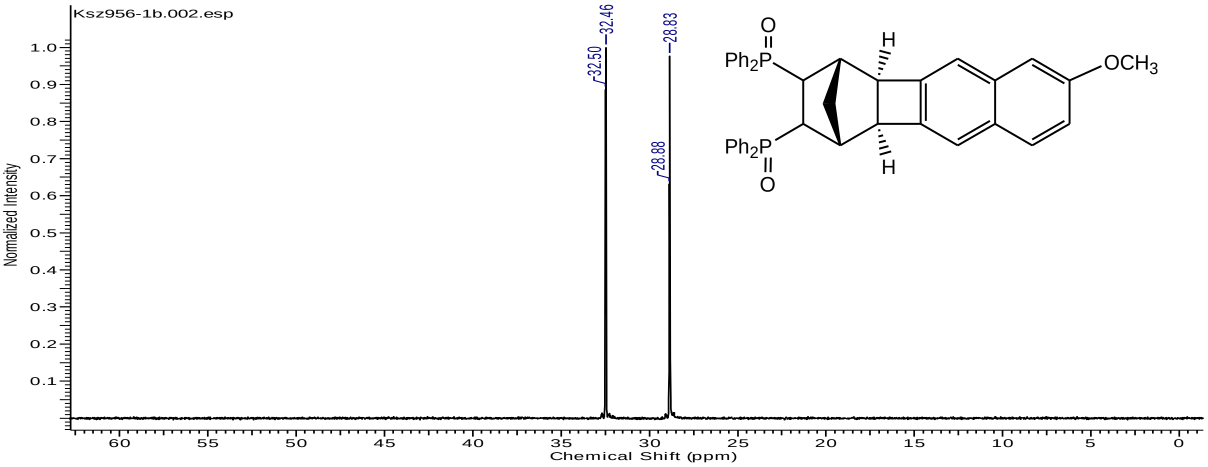

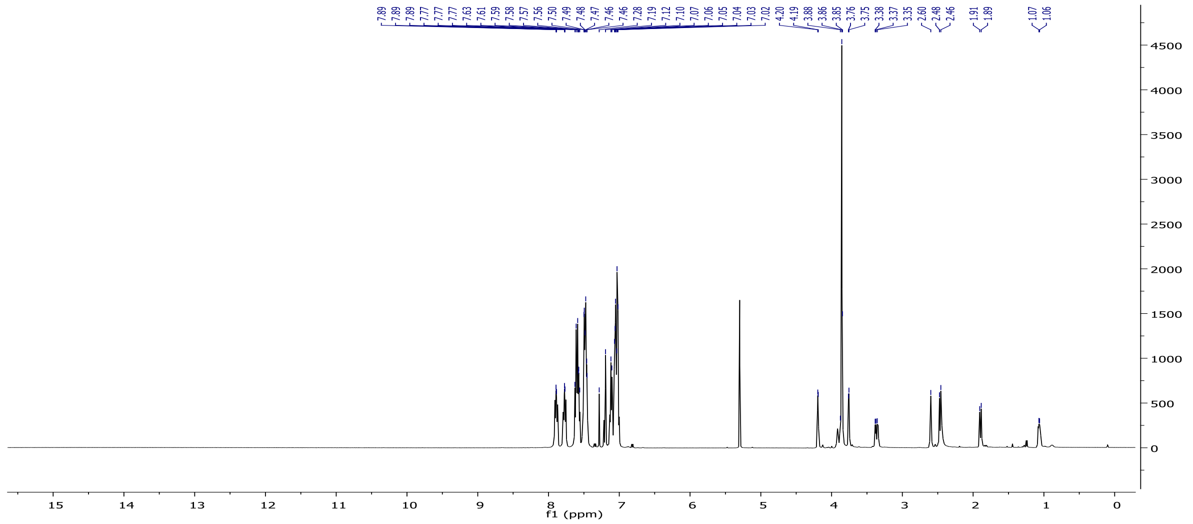

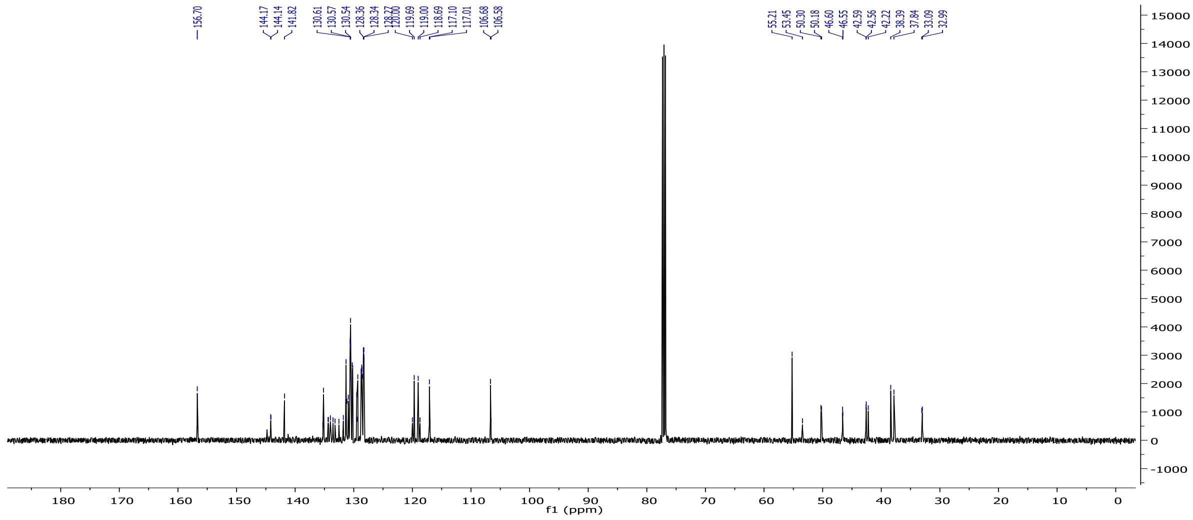

8c,9,10,11,12,12a-Hexahydro-9,12-methanobenzo[3,4]cyclobuta[1,2-*l*]phenanthrene-10,11-diylbis(diphenylphosphane) dioxide (*rac*-4p)

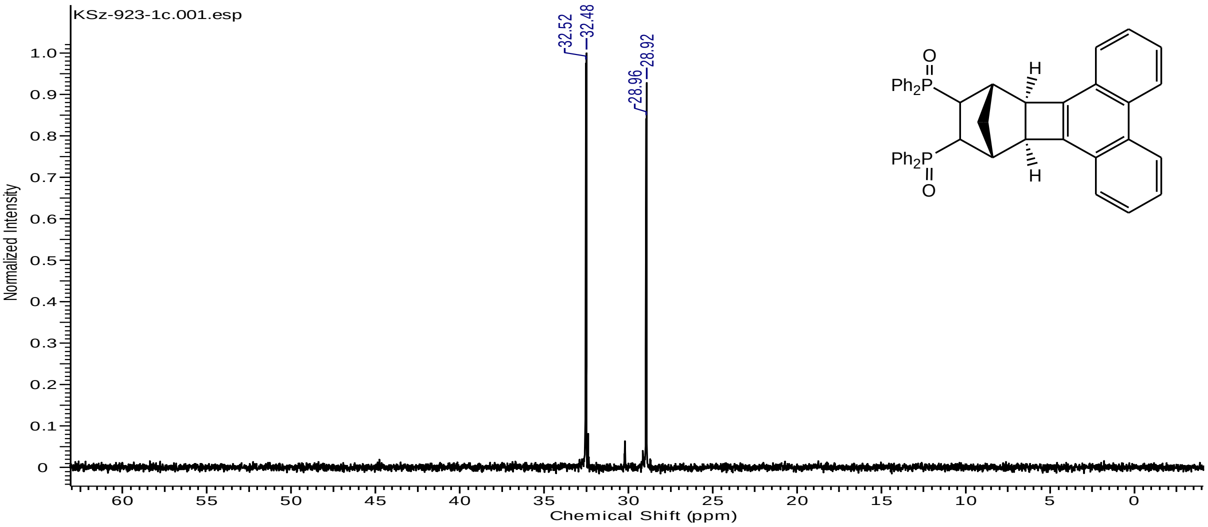

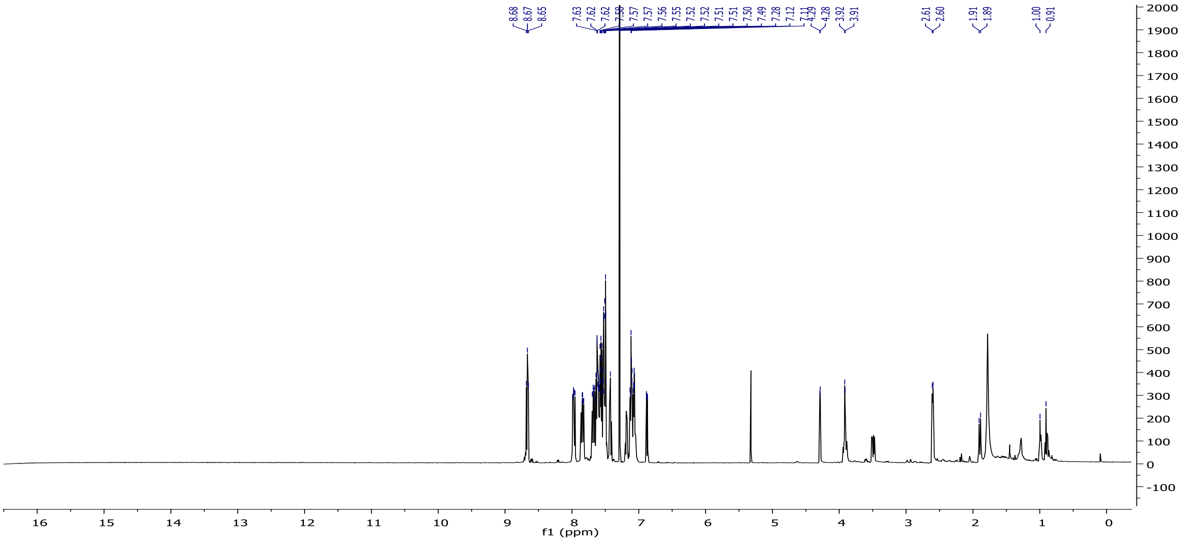

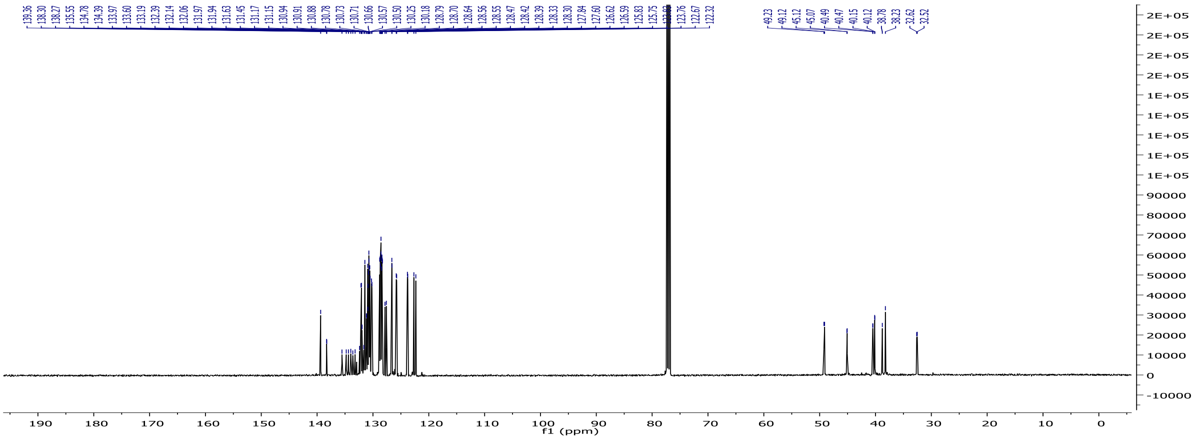

[(1*R*,2*R*,9*S*,10*S*,11*R*,12*R*)-4-Phenyltetracyclo[8.2.1.0^{2,9}.0^{3,8}]trideca-3,5,7-triene-11,12-diyl]bis(diphenylphosphane) dioxide (*R*,*R*-4q)

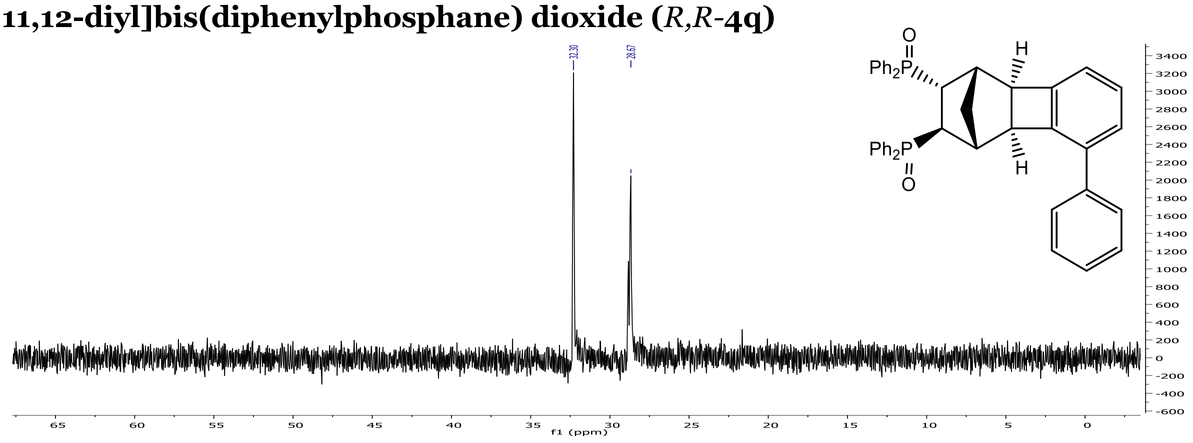

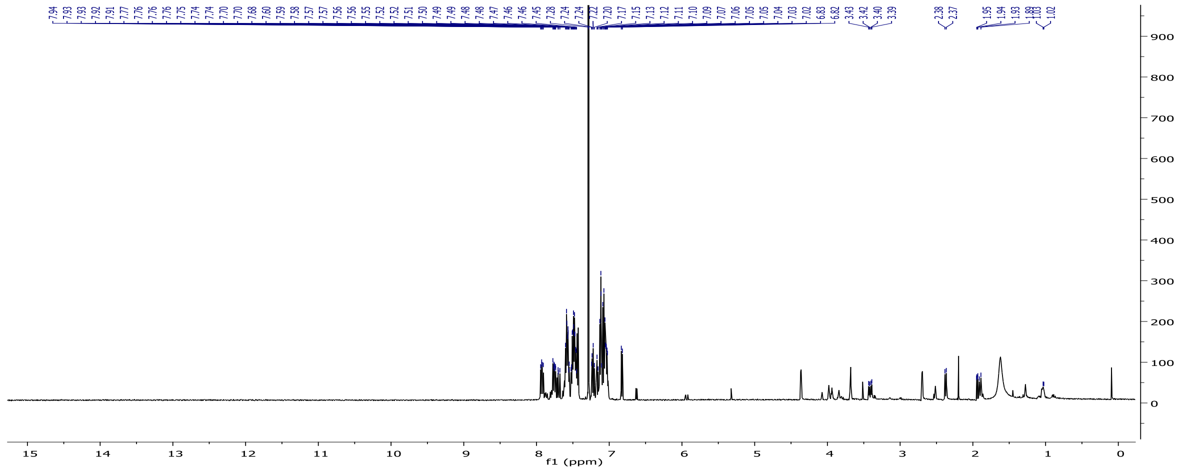

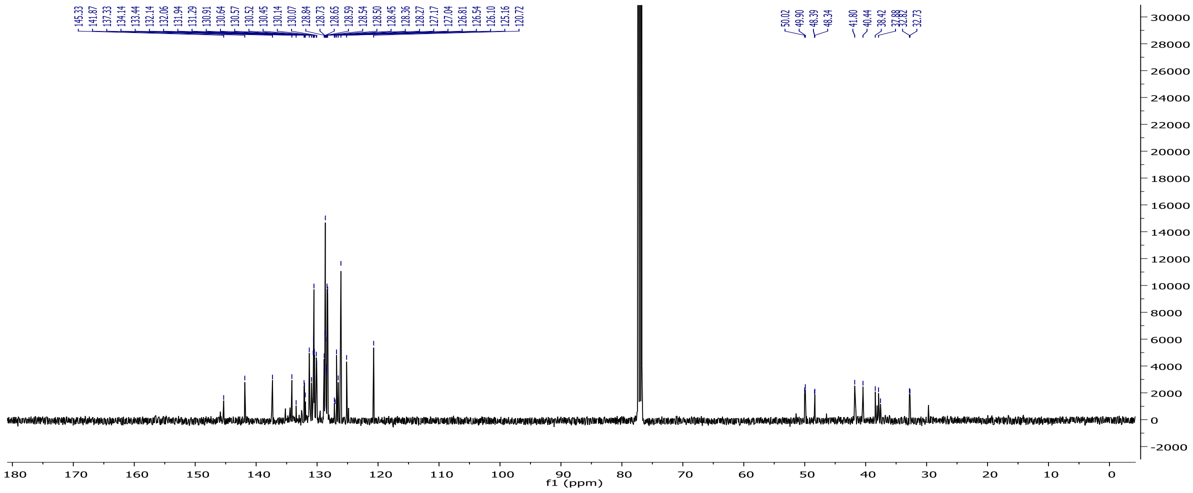

[(1*R*,2*R*,9*S*,10*S*,11*R*,12*R*)-4-Phenyltetracyclo[8.2.1.0^{2,9}.0^{3,8}]trideca-3,5,7-triene-11,12-diyl]bis(diphenylphosphane) *(R,R)*-6q

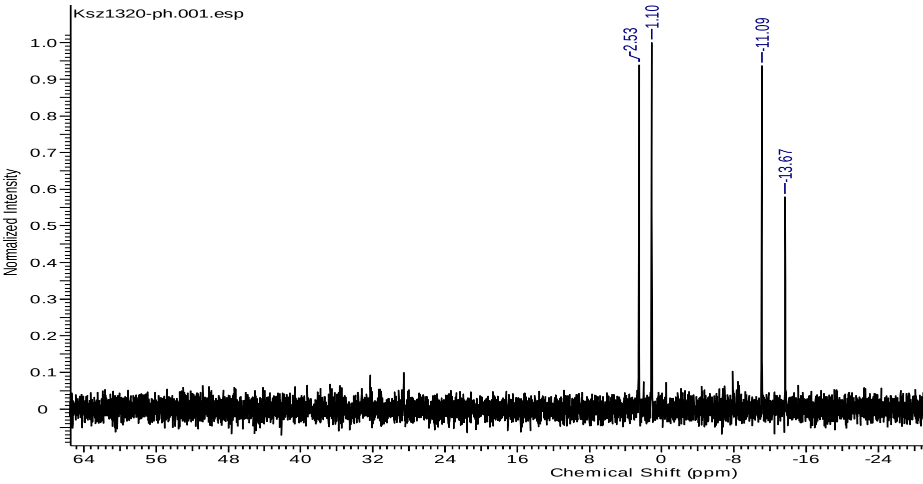

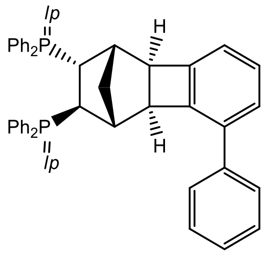

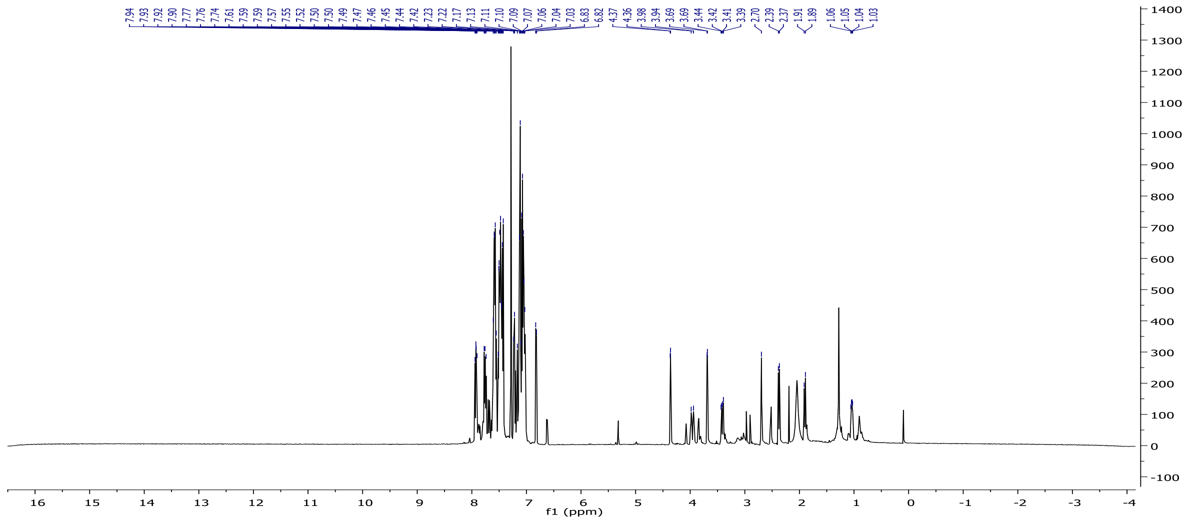

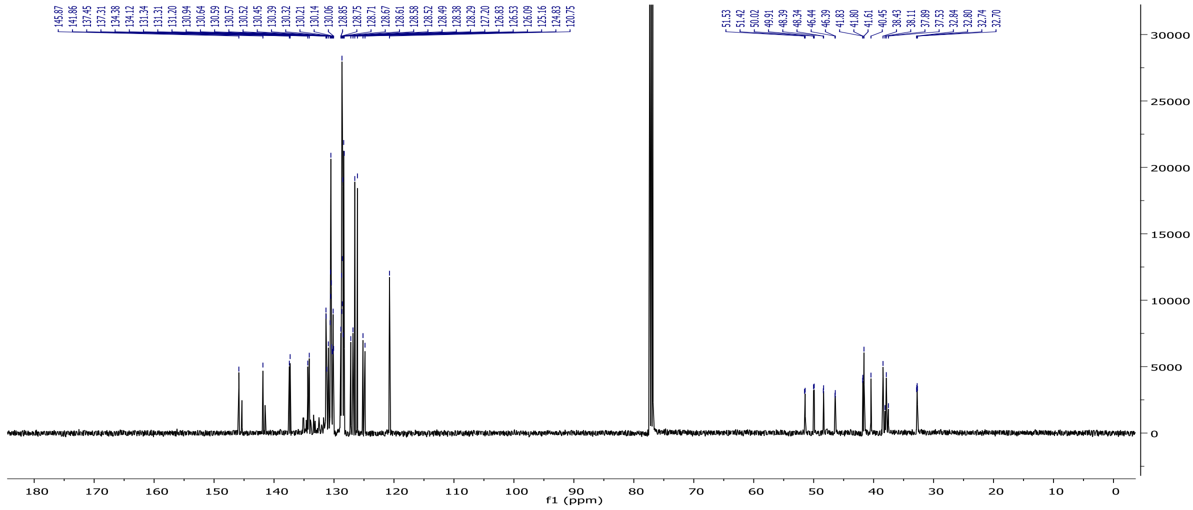

[Pd(**6q**)Cl_2]

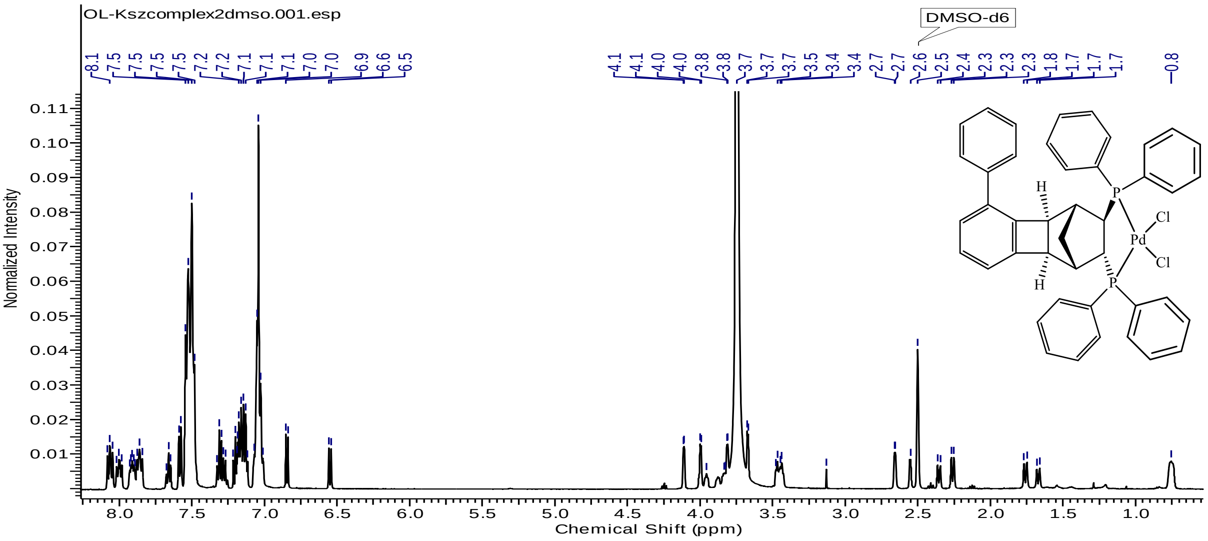
OL-Kszcomplex2dmso.001.esp
DMSO-d6
Normalized Intensity
Chemical Shift (ppm)

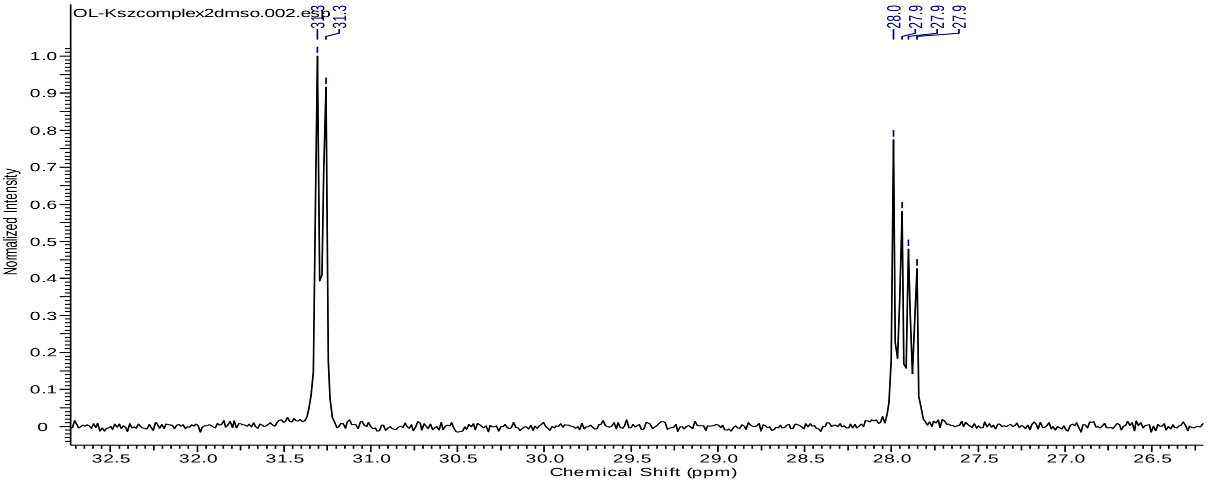
OL-Kszcomplex2dmso.002.esp
Normalized Intensity
Chemical Shift (ppm)

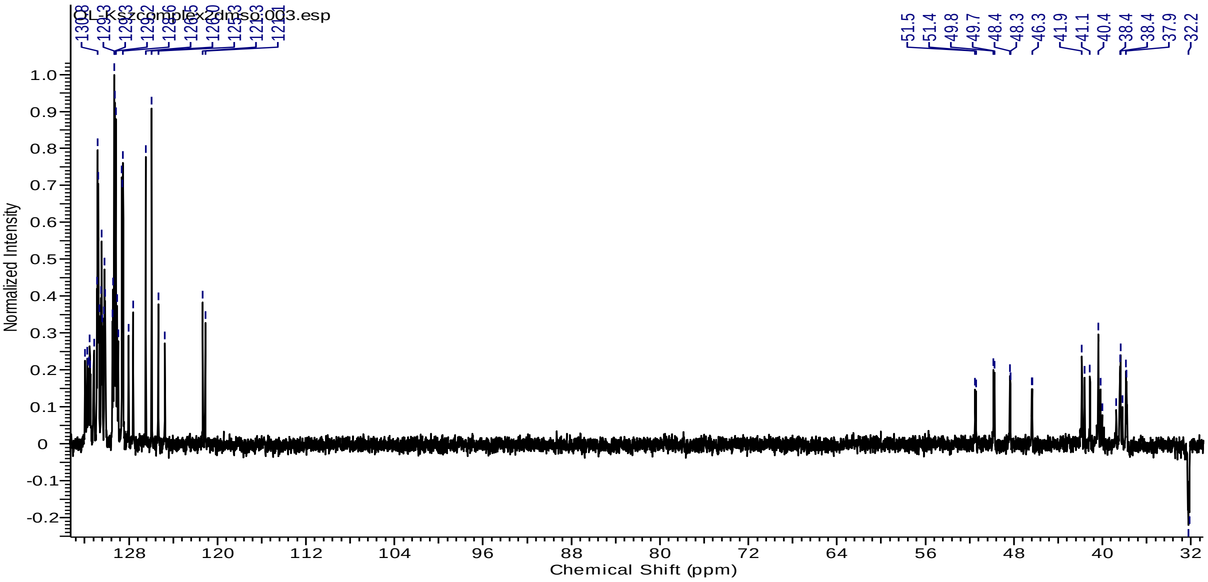
OL-Kszcomplex2dmso.003.esp
Normalized Intensity
Chemical Shift (ppm)

Dimethyl 2-[(E, S)-1-methyl-3-phenylprop-2-en-1-yl]malonate

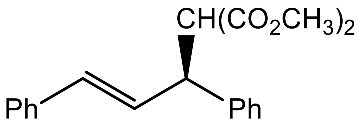

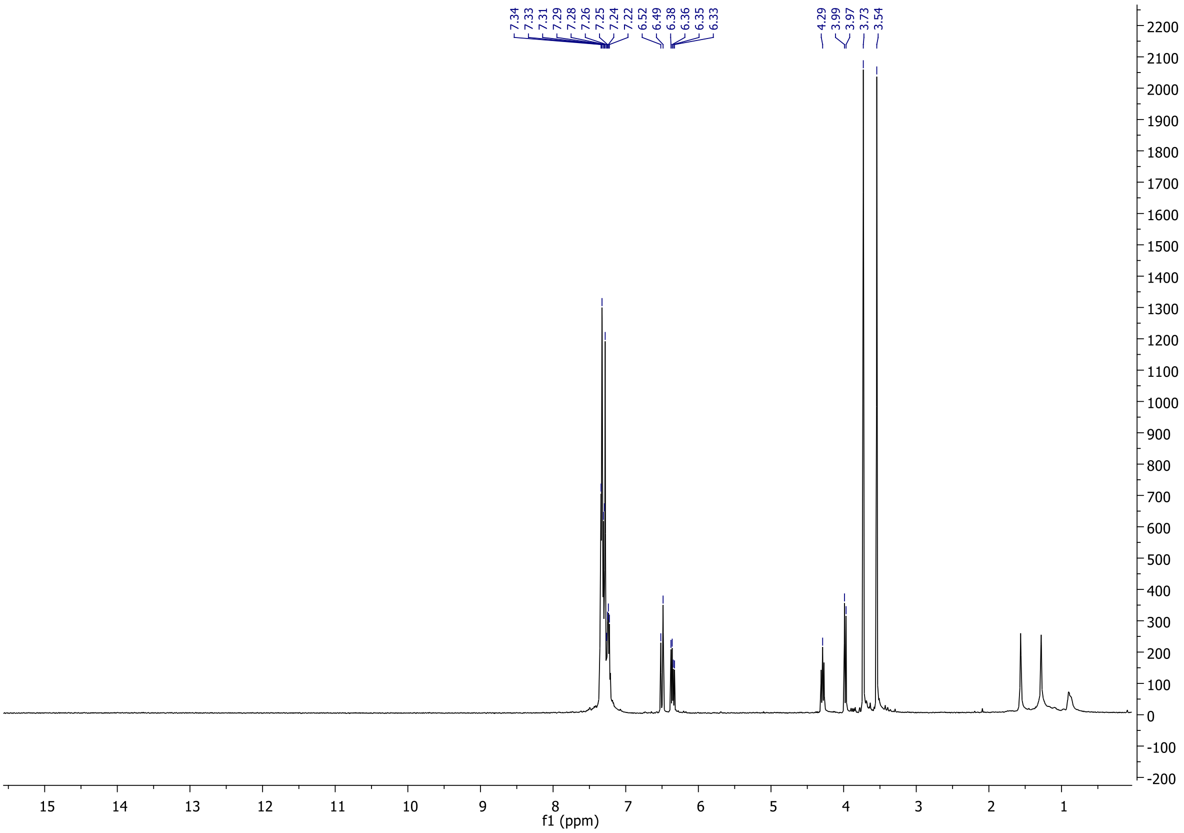

7. REFERENCES

1. Łapczuk-Krygier, A.; Ponikiewski, Ł.; Jasiński, R., The crystal structure of (1RS,4RS,5RS,6SR)-5-cyano-5-nitro-6-phenyl-bicyclo[2.2.1]hept-2-ene. *Crystallography Reports* **2014,** 59, (7), 961-963, DOI 10.1134/s1063774514070128.
2. Jasiński, R.; Kwiatkowska, M.; Sharnin, V.; Barański, A., Experimental and theoretical studies of Diels–Alder reaction between methyl (Z)-2-nitro-3-(4-nitrophenyl)-2-propenoate and cyclopentadiene. *Monatshefte für Chemie - Chemical Monthly* **2013,** 144, (3), 327-335, DOI 10.1007/s00706-012-0885-3.
3. Szwaczko, K.; Demchuk, O. M.; Mirosław, B.; Strzelecka, D.; Pietrusiewicz, K. M., Straightforward approach to norbornene core based chiral ligands by tandem cross dehydrogenative coupling reactions. *Tetrahedron Letters* **2016,** 57, (31), 3491-3495, DOI 10.1016/j.tetlet.2016.06.104.
4. Jin, M.-J.; Takale, V. B.; Sarkar, M. S.; Kim, Y.-M., Highly enantioselective Pd-catalyzed allylic alkylation using new chiral ferrocenylphosphinoimidazolidine ligands. *Chemical Communications* **2006**, (6), 663, DOI 10.1039/b515283f.
5. Rigaku Oxford Diffraction. CrysAlis PRO ver. 1.171.38.46. Rigaku Oxford Diffraction, **2015**, Yarnton, England.
6. Sheldrick, G. M., SHELXT– Integrated space-group and crystal-structure determination. *Acta Crystallographica Section A Foundations and Advances* **2015,** 71, (1), 3-8, DOI 10.1107/S2053273314026370.
7. Dolomanov, O. V.; Bourhis, L. J.; Gildea, R. J.; Howard, J. A. K.; Puschmann, H., OLEX2: a complete structure solution, refinement and analysis program. *Journal of Applied Crystallography* **2009,** 42, (2), 339-341, DOI 10.1107/S0021889808042726.
8. Zhao, Y.; Truhlar, D. G., The M06 suite of density functionals for main group thermochemistry, thermochemical kinetics, noncovalent interactions, excited states, and transition elements: two new functionals and systematic testing of four M06-class functionals and 12 other functionals. *Theoretical Chemistry Accounts* **2007,** 120, (1-3), 215-241, DOI 10.1007/s00214-007-0310-x.
9. Frisch, M.J., Trucks, G.W., Schlegel, H.B., Scuseria, G.E., Robb, M.A., Cheeseman, J.R., Scalmani, G., Barone, V., Mennucci, B., Petersson, G.A., Nakatsuji, H., Caricato, M., Li, X., Hratchian, H.P., Izmaylov, A.F., Bloino, J., Zheng, G., Sonnenberg, J.L., Hada, M., Ehara, M., Toyota, K., Fukuda, R., Hasegawa, J., Ishida, M., Nakajima, T., Honda, Y., Kitao, O., Nakai, H., Vreven, T., Montgomery Jr., J.A., Peralta, J.E., Ogliaro, F., Bearpark, M., Heyd, J.J., Brothers, E., Kudin, K.N., Staroverov, V.N., Kobayashi, R., Normand, J., Raghavachari, K., Rendell, A., Burant, J.C., Iyengar, S.S., Tomasi, J., Cossi, M., Rega, N., Millam, J.M., Klene, M., Knox, J.E., Cross, J.B., Bakken, V., Adamo, C., Jaramillo, J., Gomperts, R., Stratmann, R.E., Yazyev, O., Austin, A.J., Cammi, R., Pomelli, C., Ochterski, J.W., Martin, R.L., Morokuma, K., Zakrzewski, V.G., Voth, G.A., Salvador, P., Dannenberg, J.J., Dapprich, S., Daniels, A.D., Farkas, O., Foresman, J.B., Ortiz, J.V., Cioslowski, J. and Fox, D.J. *Gaussian 09, Revision B.01,* **2010**, Gaussian Inc., Wallingford.
